# Author Correction: Suppression of Transposable Elements in Leukemic Stem Cells

**DOI:** 10.1038/s41598-018-21280-6

**Published:** 2018-03-06

**Authors:** Anthony R. Colombo, Asif Zubair, Devi Thiagarajan, Sergey Nuzhdin, Timothy J. Triche, Giridharan Ramsingh

**Affiliations:** 1 Keck School of Medicine of University of Southern California, Jane Anne Nohl Division of Hematology and Center for the Study of Blood Diseases, Los Angeles, California 90033 USA; 2 University of Southern California, Department of Molecular and Computational Biology, Los Angeles, CA 90089-2910 USA; 3 Langone Medical Center of New York University School of Medicine, Endocrinology Division for the Study of Diabetes, 550 1st Avenue, New York, NY 10016 USA

Correction to: *Scientific Reports* 10.1038/s41598-017-07356-9, published online 01 August 2017

A supplementary dataset containing methods was omitted from the original version of this Article. This has been corrected in the HTML version of the Article; the PDF version was correct at time of publication.

