# Supplementary material for: Suppression of Transposable Elements in Leukemic Stem Cells
Source: Sci Rep. 2017 Aug 1;7:7029. doi: 10.1038/s41598-017-07356-9 (PMC5539300; doi:10.1038/s41598-017-07356-9)
Supplement: Supplementary file 1 — Supplemental figures [file 41598_2017_7356_MOESM1_ESM.pdf]

Suppression of Transposable Elements in Leukemic Stem Cells

Supplementary Figures

Anthony R. Colombo, Asif Zubair, Devi Thiagarajan, Sergey Nuzhdin, Timothy J. Triche Jr., Giridharan Ramsingh

Supplement figure 1

Differential transposable element expression
pairwise comparison LSC-pHSC

a

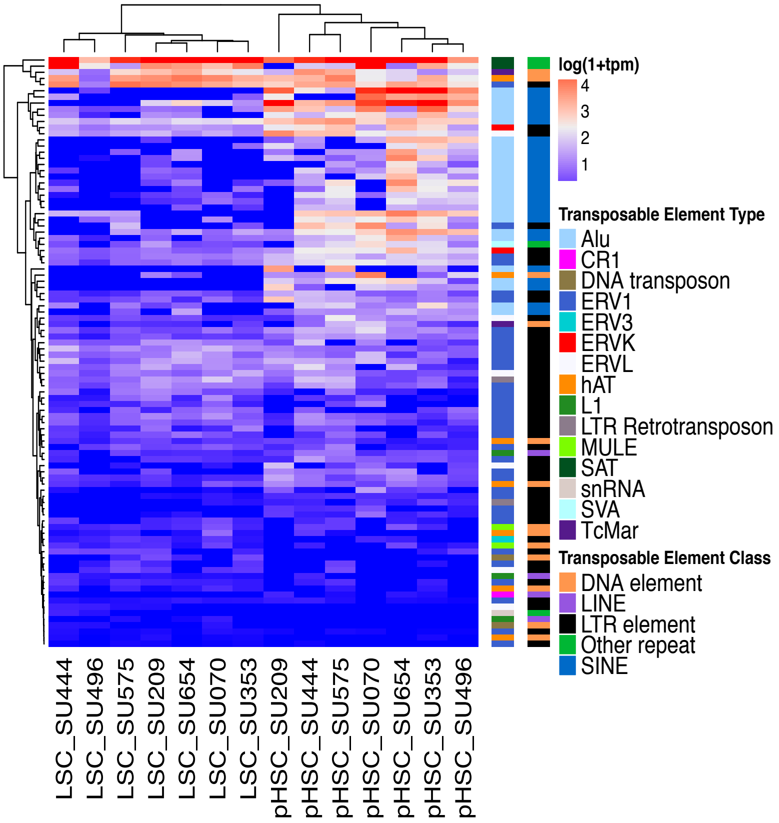

Differential transposable element expression
pairwise comparison LSC-Blast

b

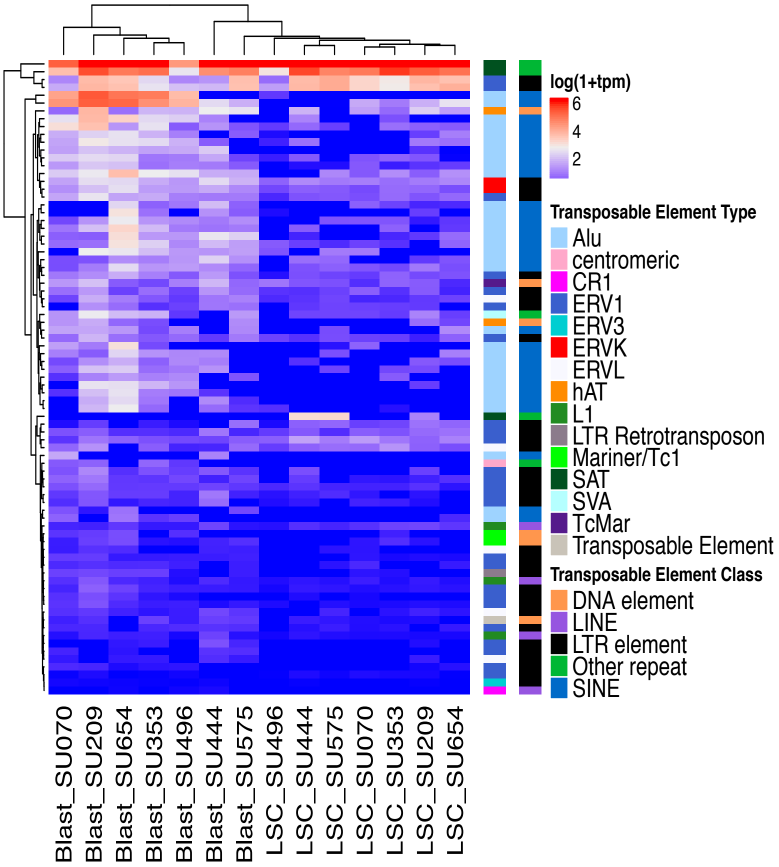

Supplement figure 2

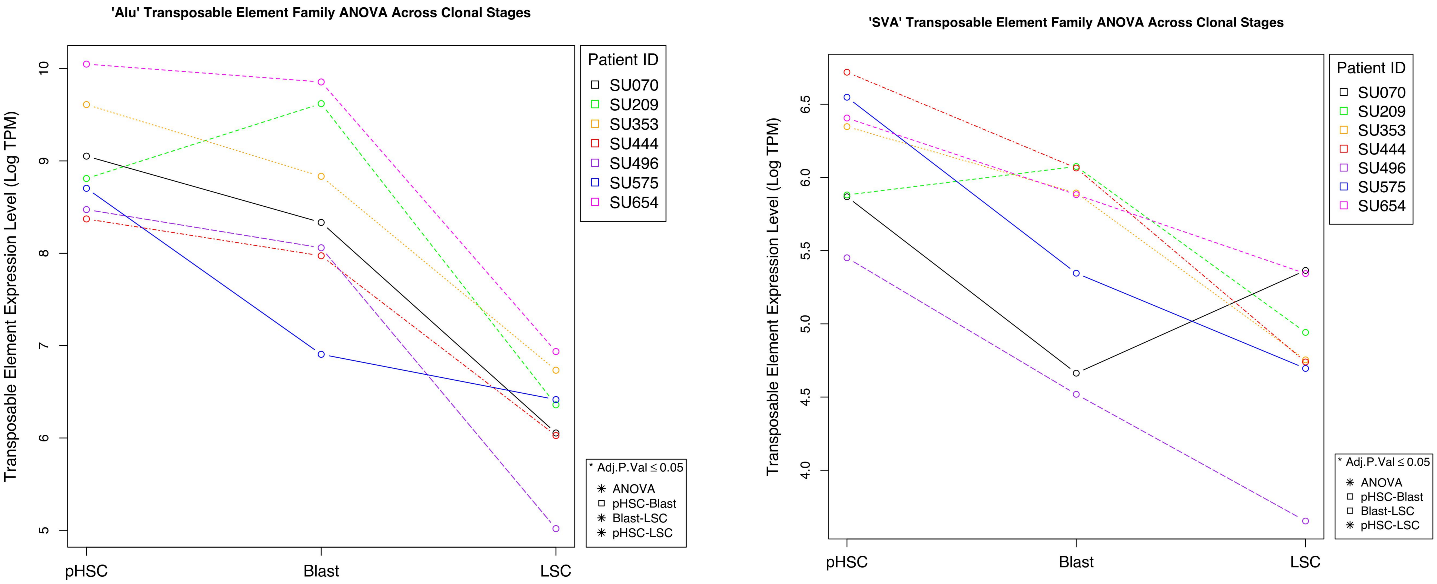

ANOVA on Individual Patient Expression Levels For Specific Transposable Element Types. The axis labeling is similar to Figure 1.C. The results from the ANOVA, which tested for a statistical difference of means across all clonal points, and with pairwise comparisons is located in the bottom right box of each figure. The Bonferroni multiple testing adjusted p-values are denoted 'Adj.P.Val' where an asterisk denotes a significant result after adjusting for multiple testing. For exact p.values see Supplementary Table 1.

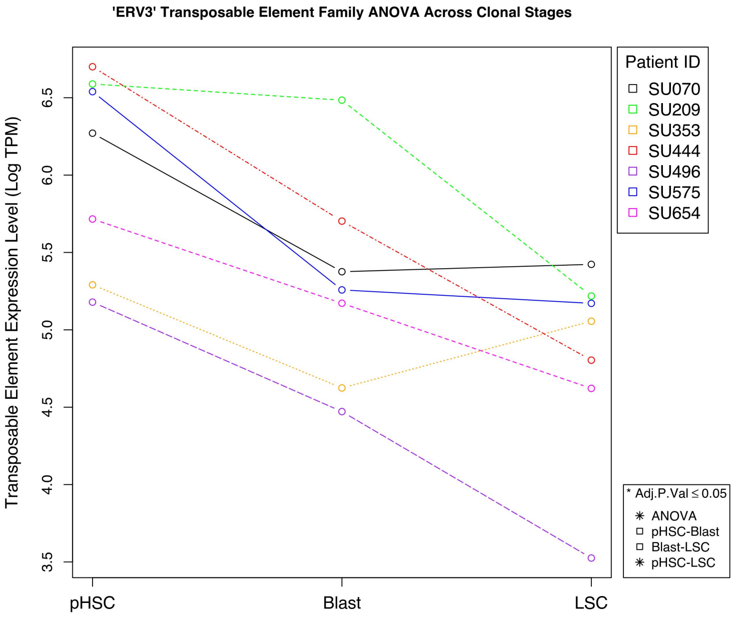
'ERV3' Transposable Element Family ANOVA Across Clonal Stages
Transposable Element Expression Level (Log TPM)
3.5
4.0
4.5
5.0
5.5
6.0
6.5
pHSC
Blast
LSC
Patient ID
SU070
SU209
SU353
SU444
SU496
SU575
SU654
* Adj.P.Val ≤ 0.05
ANOVA
pHSC-Blast
Blast-LSC
pHSC-LSC

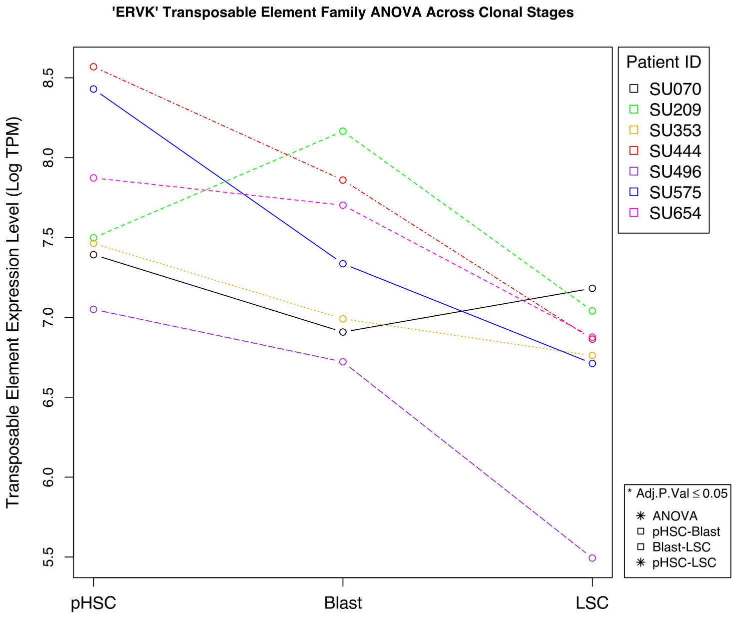
'ERVK' Transposable Element Family ANOVA Across Clonal Stages
Transposable Element Expression Level (Log TPM)
5.5
6.0
6.5
7.0
7.5
8.0
8.5
pHSC
Blast
LSC
Patient ID
SU070
SU209
SU353
SU444
SU496
SU575
SU654
* Adj.P.Val ≤ 0.05
ANOVA
pHSC-Blast
Blast-LSC
pHSC-LSC

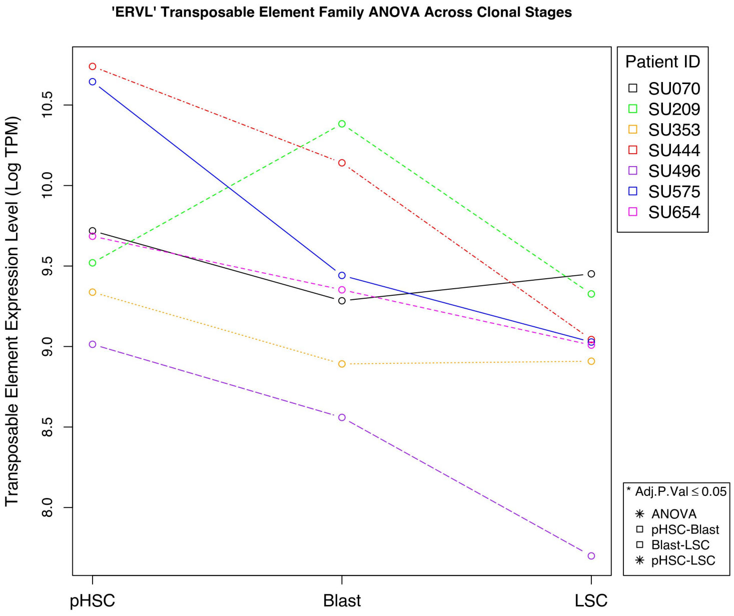
'ERVL' Transposable Element Family ANOVA Across Clonal Stages
Transposable Element Expression Level (Log TPM)
8.0
8.5
9.0
9.5
10.0
10.5
pHSC
Blast
LSC
Patient ID
SU070
SU209
SU353
SU444
SU496
SU575
SU654
* Adj.P.Val ≤ 0.05
ANOVA
pHSC-Blast
Blast-LSC
pHSC-LSC

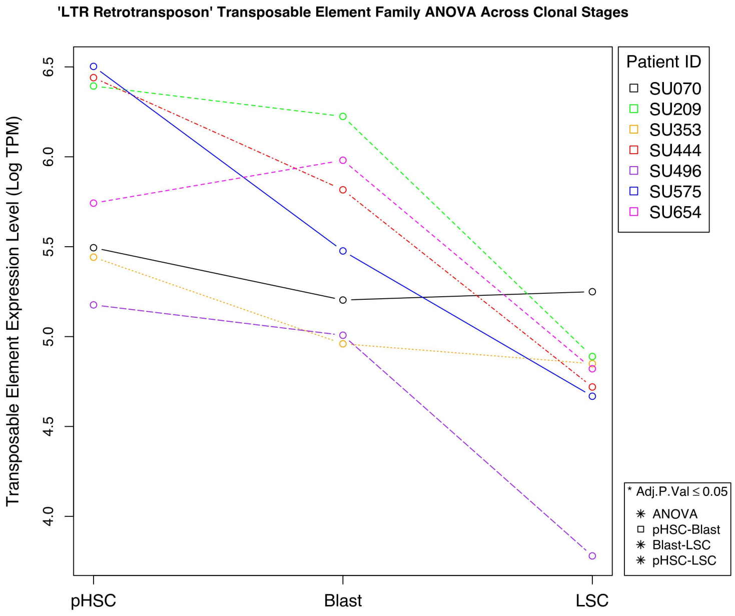
'LTR Retrotransposon' Transposable Element Family ANOVA Across Clonal Stages
Transposable Element Expression Level (Log TPM)
4.0
4.5
5.0
5.5
6.0
6.5
pHSC
Blast
LSC
Patient ID
SU070
SU209
SU353
SU444
SU496
SU575
SU654
* Adj.P.Val ≤ 0.05
ANOVA
pHSC-Blast
Blast-LSC
pHSC-LSC

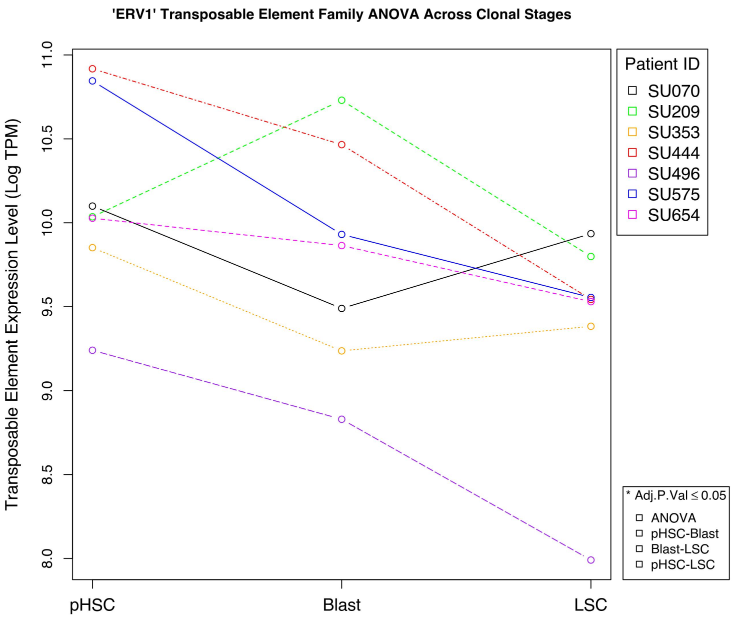
'ERV1' Transposable Element Family ANOVA Across Clonal Stages
Transposable Element Expression Level (Log TPM)
8.0
8.5
9.0
9.5
10.0
10.5
11.0
pHSC
Blast
LSC
Patient ID
SU070
SU209
SU353
SU444
SU496
SU575
SU654
* Adj.P.Val ≤ 0.05
ANOVA
pHSC-Blast
Blast-LSC
pHSC-LSC

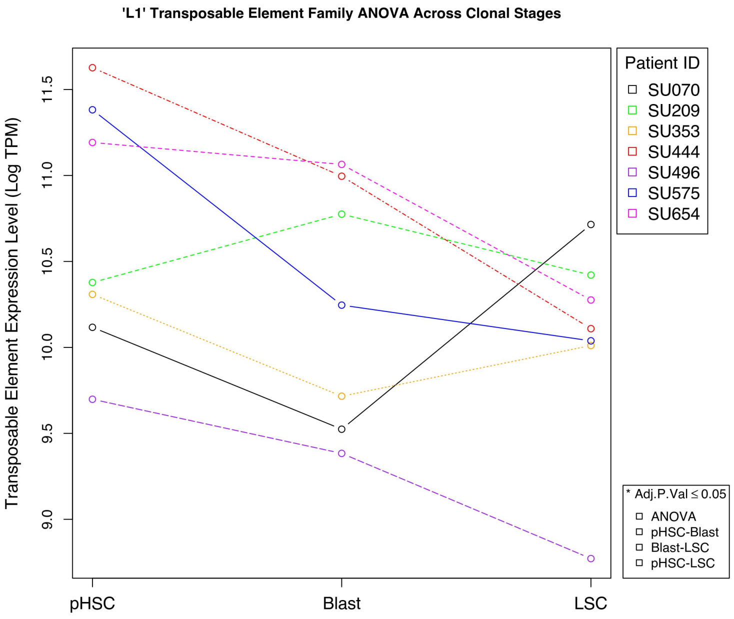
'L1' Transposable Element Family ANOVA Across Clonal Stages
Transposable Element Expression Level (Log TPM)
9.0
9.5
10.0
10.5
11.0
11.5
pHSC
Blast
LSC
Patient ID
SU070
SU209
SU353
SU444
SU496
SU575
SU654
* Adj.P.Val ≤ 0.05
ANOVA
pHSC-Blast
Blast-LSC
pHSC-LSC

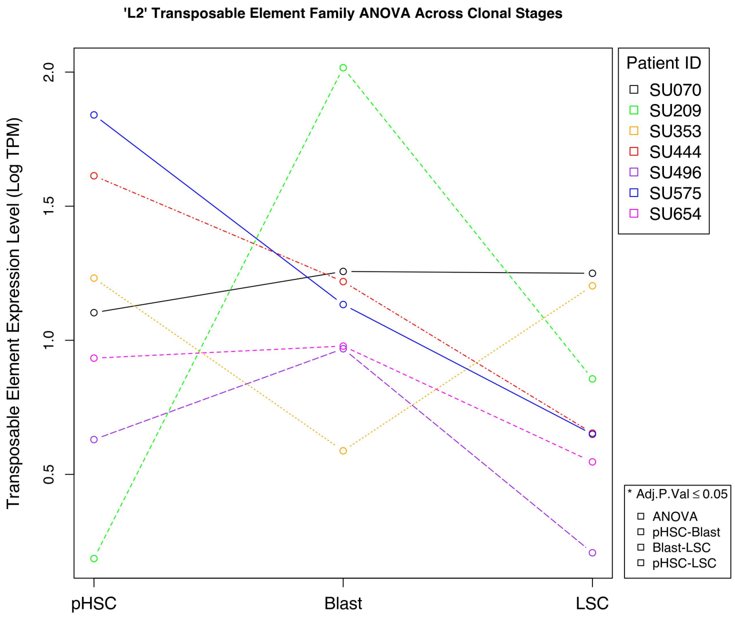
'L2' Transposable Element Family ANOVA Across Clonal Stages
Transposable Element Expression Level (Log TPM)
0.5
1.0
1.5
2.0
pHSC
Blast
LSC
Patient ID
SU070
SU209
SU353
SU444
SU496
SU575
SU654
* Adj.P.Val ≤ 0.05
ANOVA
pHSC-Blast
Blast-LSC
pHSC-LSC

'Transposable Element' Transposable Element Family ANOVA Across Clonal Stages

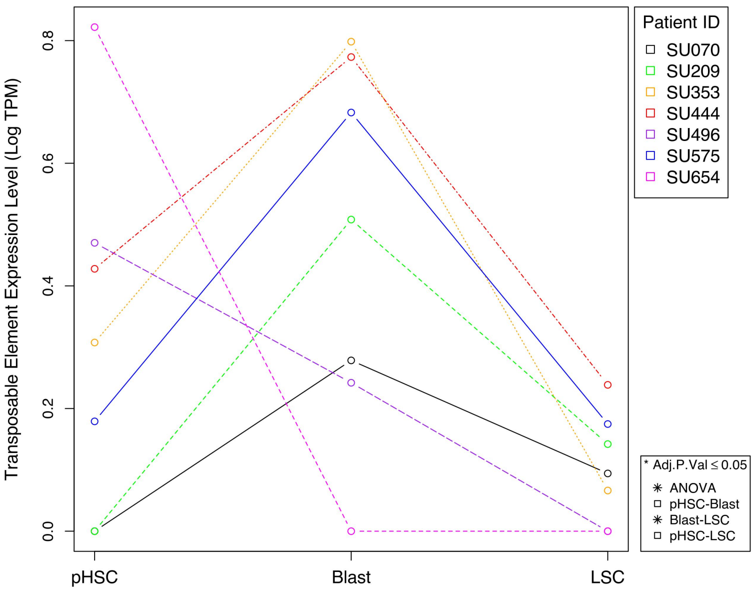

'telo' Transposable Element Family ANOVA Across Clonal Stages

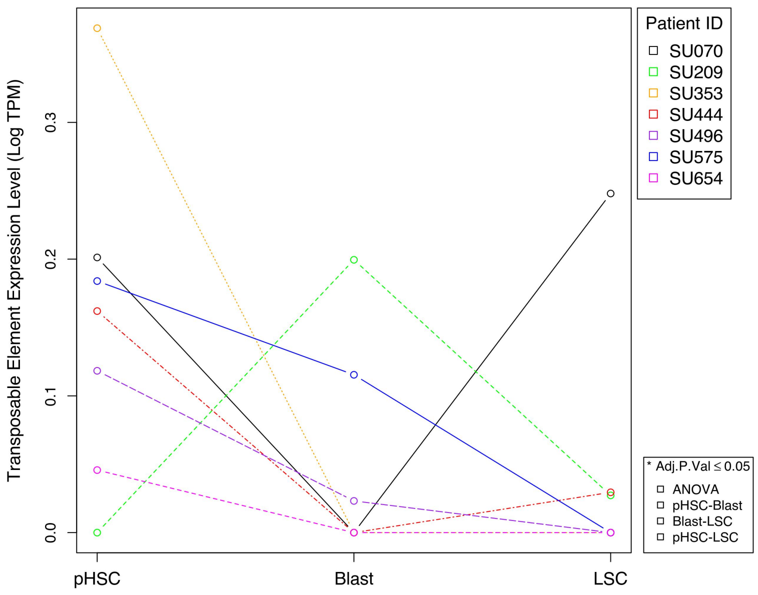

'hAT' Transposable Element Family ANOVA Across Clonal Stages

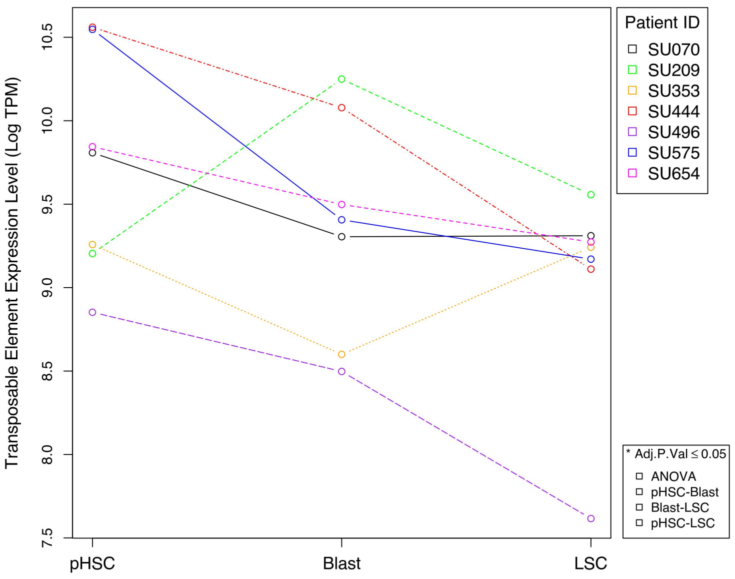

'centromeric' Transposable Element Family ANOVA Across Clonal Stages

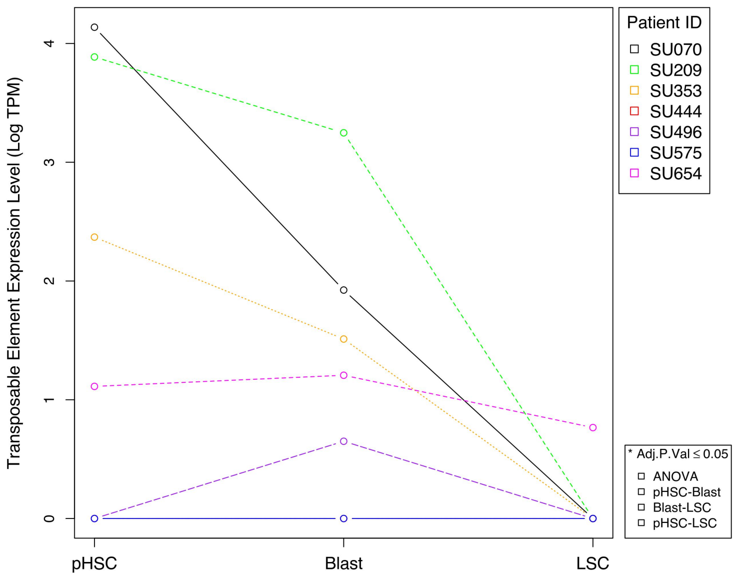

'DNA transposon' Transposable Element Family ANOVA Across Clonal Stages

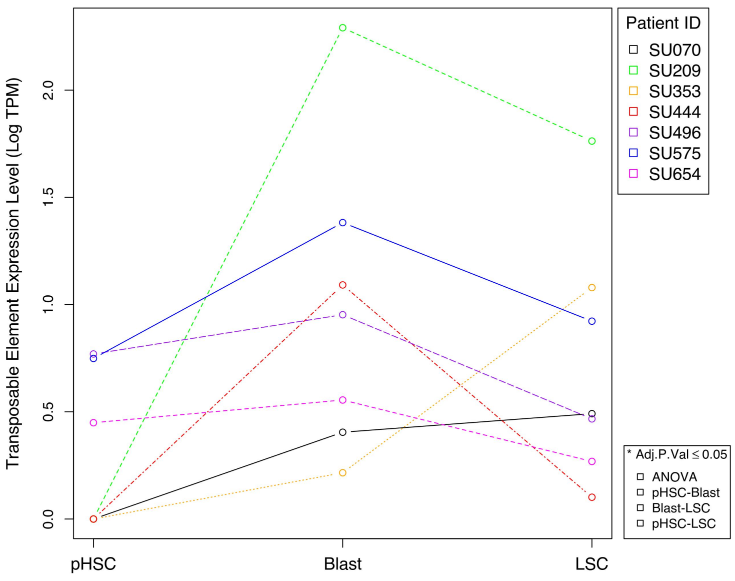

'TcMar' Transposable Element Family ANOVA Across Clonal Stages

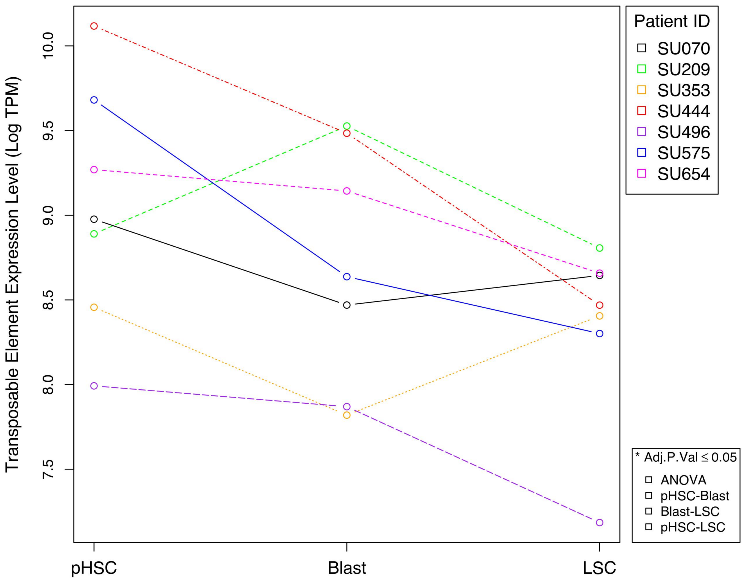

'Mariner/Tc1' Transposable Element Family ANOVA Across Clonal Stages

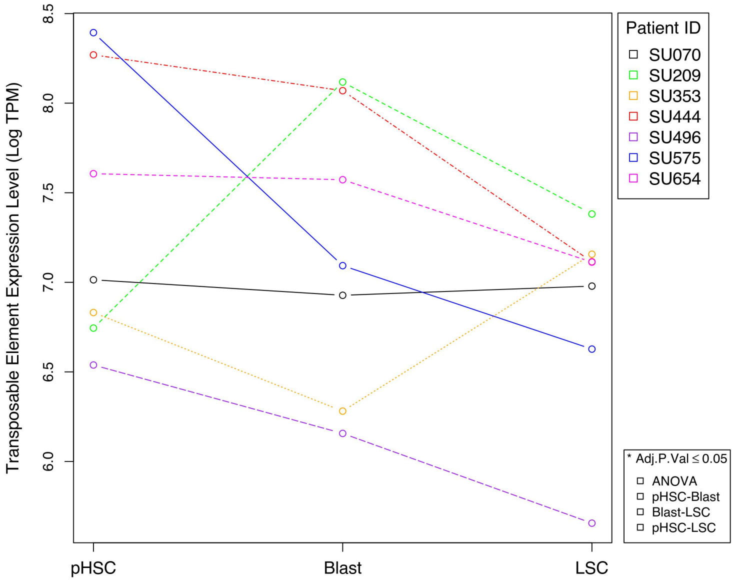

'MIR' Transposable Element Family ANOVA Across Clonal Stages

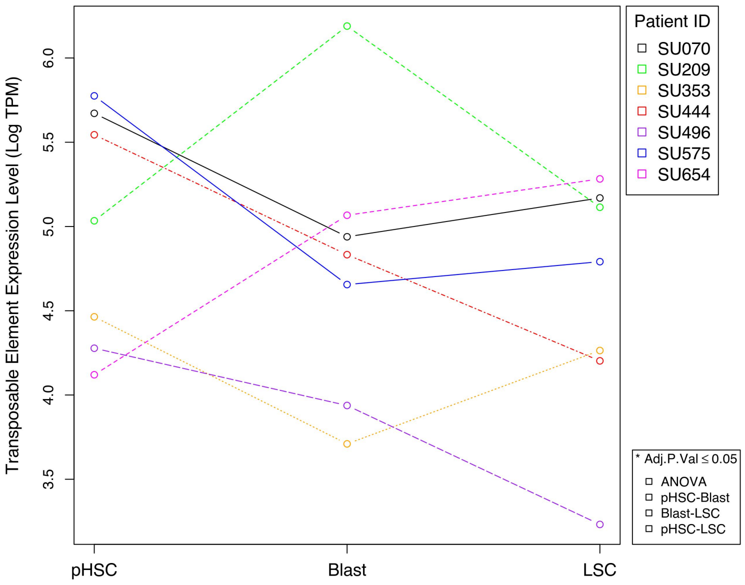

Supplement figure 3

a

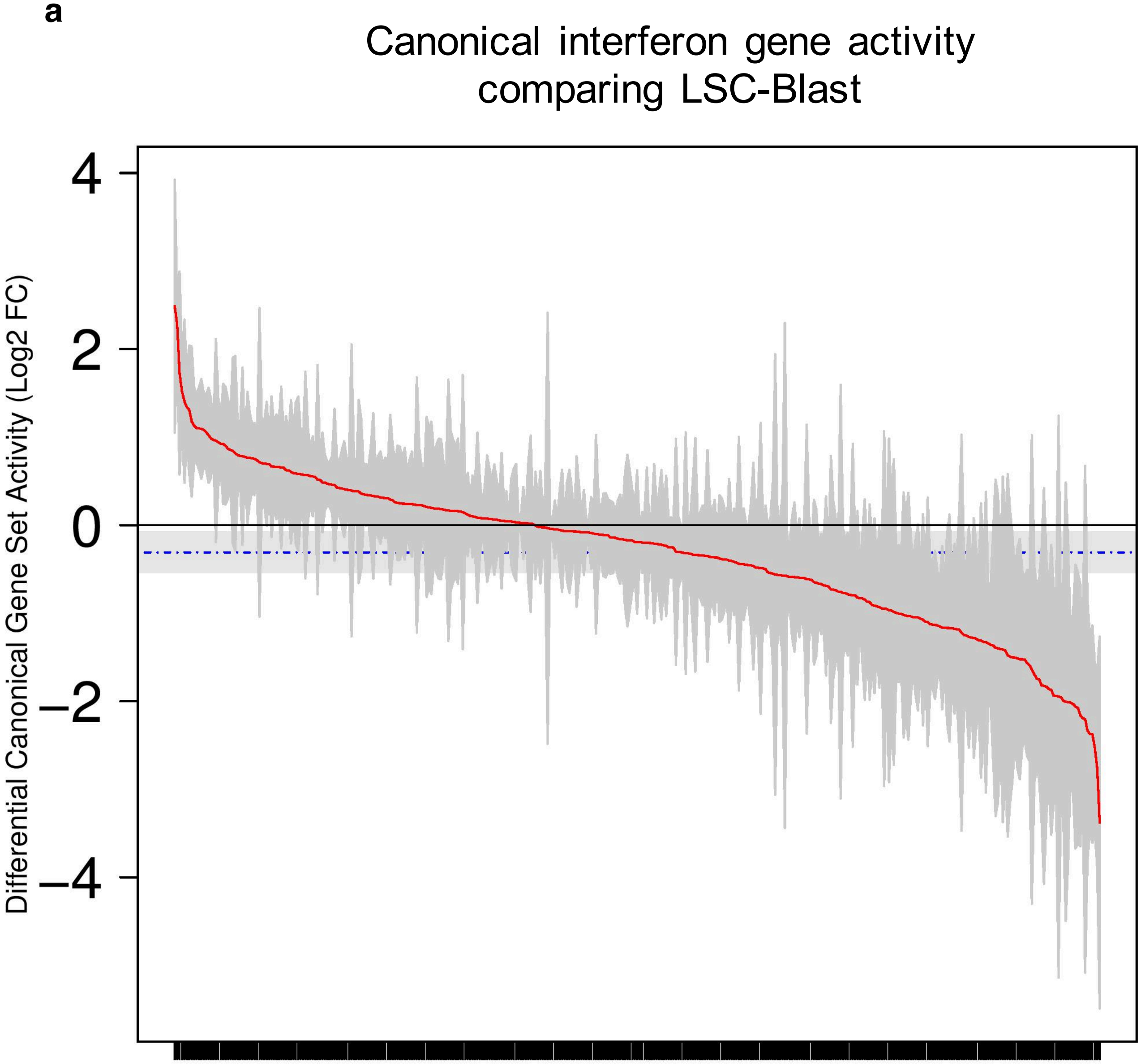

The Interferon gene set contained 433 unique genes from Gene Ontology (MSigDB) merged from 29 interferon related pathways available in GO. The each x-axis tick represents a gene in the set. The differential mean is located on the y-axis with a grey band as the confidence interval covering the mean plotted in red. The global pathway activity is a dashed blue line. The comparison was LSC to Blast (reference) with paired measurements using patient IDs.

The dashed blue line is below 0 on y-axis indicating that the gene-set enrichment is down-activated in the comparison of LSC-Blast, indicating higher activation in Blast, and less activation in LSC.

Interferon pathway distribution plot comparing LSC-Blast

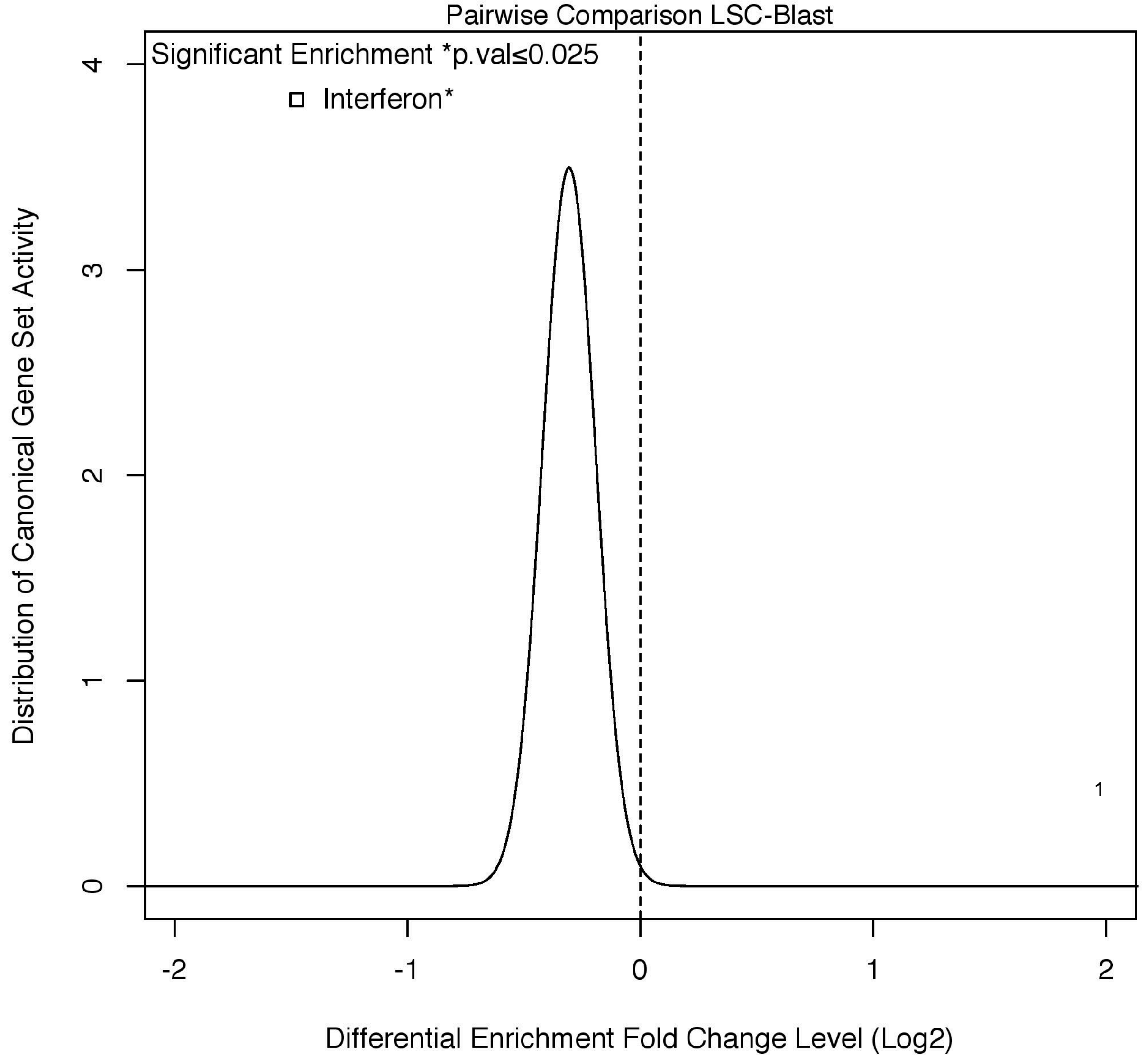

The distribution of all the interferon related pathways listed in Figure 2A with similar labeling to Figure 2B. Note that *negative* fold change indicates higher gene expression activity/signaling in Blasts compared to LSC.

DE interferon genes comparing LSC-Blast

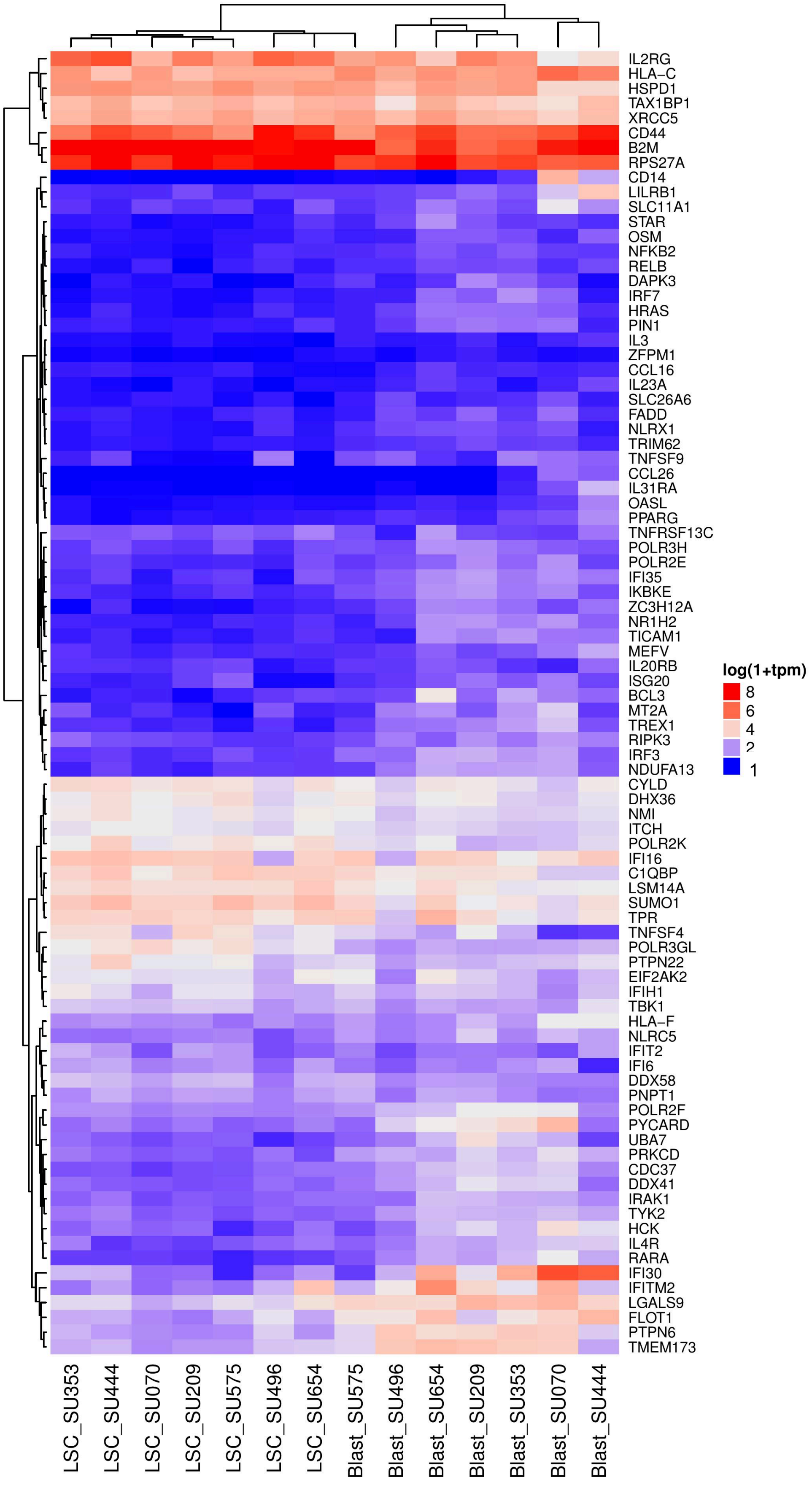

The sample groups are denoted on the x-axis with the patient ID as a suffix. The differentially expressed genes' expression values are plotted using log base 10 (1 + TPM). The y-axis denotes the genes used in the Immune gene set.

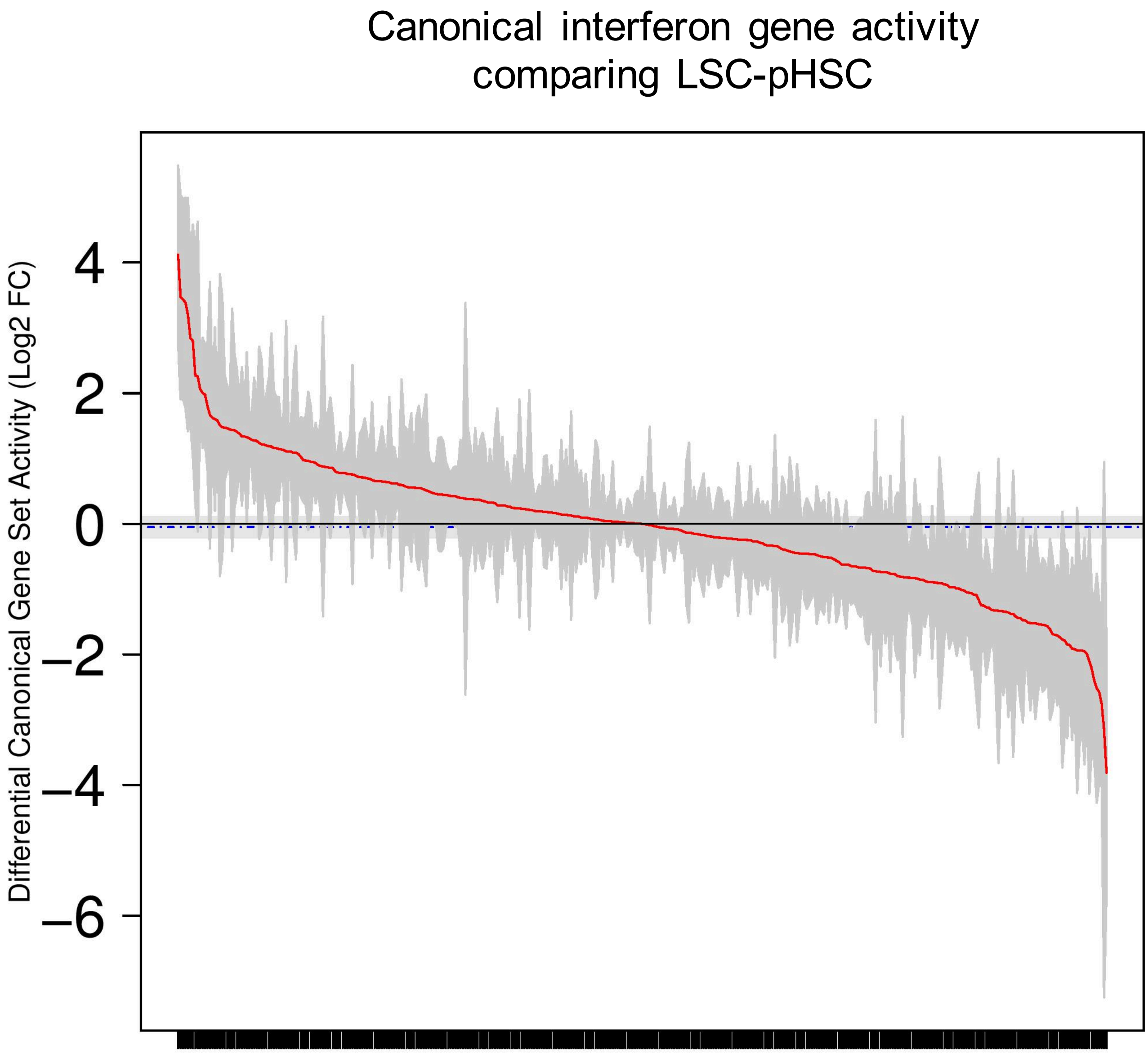

Labeling is similar to supplement 3A, however with respect to LSC-pHSC comparison.

LSC-pHSC (GO) MsigDB interferon gene-set analysis

LSC-pHSC (GO) Interferon MSigDB	log.FC	p.Value	FDR
GO_REGULATION_OF_INTERFERON_ALPHA_PRODUCTION	0.7061	3.77E-06	0.0001
GO_POSITIVE_REGULATION_OF_INTERFERON_ALPHA_PRODUCTION	0.5109	0.0001	0.0016
GO_POSITIVE_REGULATION_OF_INTERFERON_BETA_PRODUCTION	0.2474	0.0012	0.0119
GO_RESPONSE_TO_INTERFERON_BETA	-0.5467	0.0039	0.0239
GO_REGULATION_OF_INTERFERON_BETA_PRODUCTION	0.1982	0.0043	0.0239
REGULATION_OF_INTERFERON_GAMMA_BIOSYNTHETIC_PROCESS	0.4923	0.0048	0.0239
GO_CELLULAR_RESPONSE_TO_INTERFERON_BETA	-0.3394	0.0061	0.0262
INTERFERON_GAMMA_BIOSYNTHETIC_PROCESS	0.4812	0.0114	0.0429
GO_REGULATION_OF_INTERFERON_GAMMA_BIOSYNTHETIC_PROCESS	0.2863	0.0158	0.0528
GO_RESPONSE_TO_INTERFERON_ALPHA	-0.3387	0.0176	0.0528
GO_TYPE_I_INTERFERON_RECEPTOR_BINDING	0.1900	0.0216	0.0590
INTERFERON_GAMMA_PRODUCTION	0.3733	0.0271	0.0677
GO_POSITIVE_REGULATION_OF_INTERFERON_GAMMA_BIOSYNTHETIC_PROCES S	0.2639	0.0344	0.0794
GO_REGULATION_OF_TYPE_I_INTERFERON_MEDIATED_SIGNALING_PATHWAY	-0.1938	0.0804	0.1723
GO_RESPONSE_TO_TYPE_I_INTERFERON	-0.2248	0.1311	0.2622
GO_POSITIVE_REGULATION_OF_TYPE_I_INTERFERON_PRODUCTION	0.1094	0.2184	0.4096
HEMATOPOIETIN_INTERFERON_CLASSD200_DOMAIN_CYTOKINE_RECEPTOR_BIN DING	-0.0951	0.2714	0.4424
GO_INTERFERON_GAMMA_PRODUCTION	-0.1947	0.2797	0.4424
GO_REGULATION_OF_RESPONSE_TO_INTERFERON_GAMMA	-0.1173	0.2802	0.4424
GO_REGULATION_OF_INTERFERON_GAMMA_SECRETION	-0.1705	0.3554	0.5331
GO_INTERFERON_GAMMA_MEDIATED_SIGNALING_PATHWAY	0.1013	0.4748	0.6703
GO_REGULATION_OF_INTERFERON_GAMMA_PRODUCTION	-0.0674	0.4915	0.6703
INTERFERON (Combined Gene Set)	-0.0499	0.5370	0.7004
GO_POSITIVE_REGULATION_OF_INTERFERON_GAMMA_PRODUCTION	0.0608	0.5644	0.7055
GO_REGULATION_OF_TYPE_I_INTERFERON_PRODUCTION	0.0515	0.6154	0.7385
GO_NEGATIVE_REGULATION_OF_TYPE_I_INTERFERON_PRODUCTION	0.0477	0.6770	0.7628
GO_CELLULAR_RESPONSE_TO_INTERFERON_GAMMA	0.0461	0.6865	0.7628
GO_NEGATIVE_REGULATION_OF_INTERFERON_GAMMA_PRODUCTION	0.0172	0.9182	0.9837
GO_RESPONSE_TO_INTERFERON_GAMMA	0.0031	0.9960	0.9967
HEMATOPOIETIN_INTERFERON_CLASSD200_DOMAIN_CYTOKINE_RECEPTOR_AC TIVITY	-0.0013	0.9967	0.9967

Labeling similar to Figure 2A. Lists all pathways related to interferon using GO comparing LSC-pHSC.

Interferon pathway distribution plot comparing LSC-pHSC

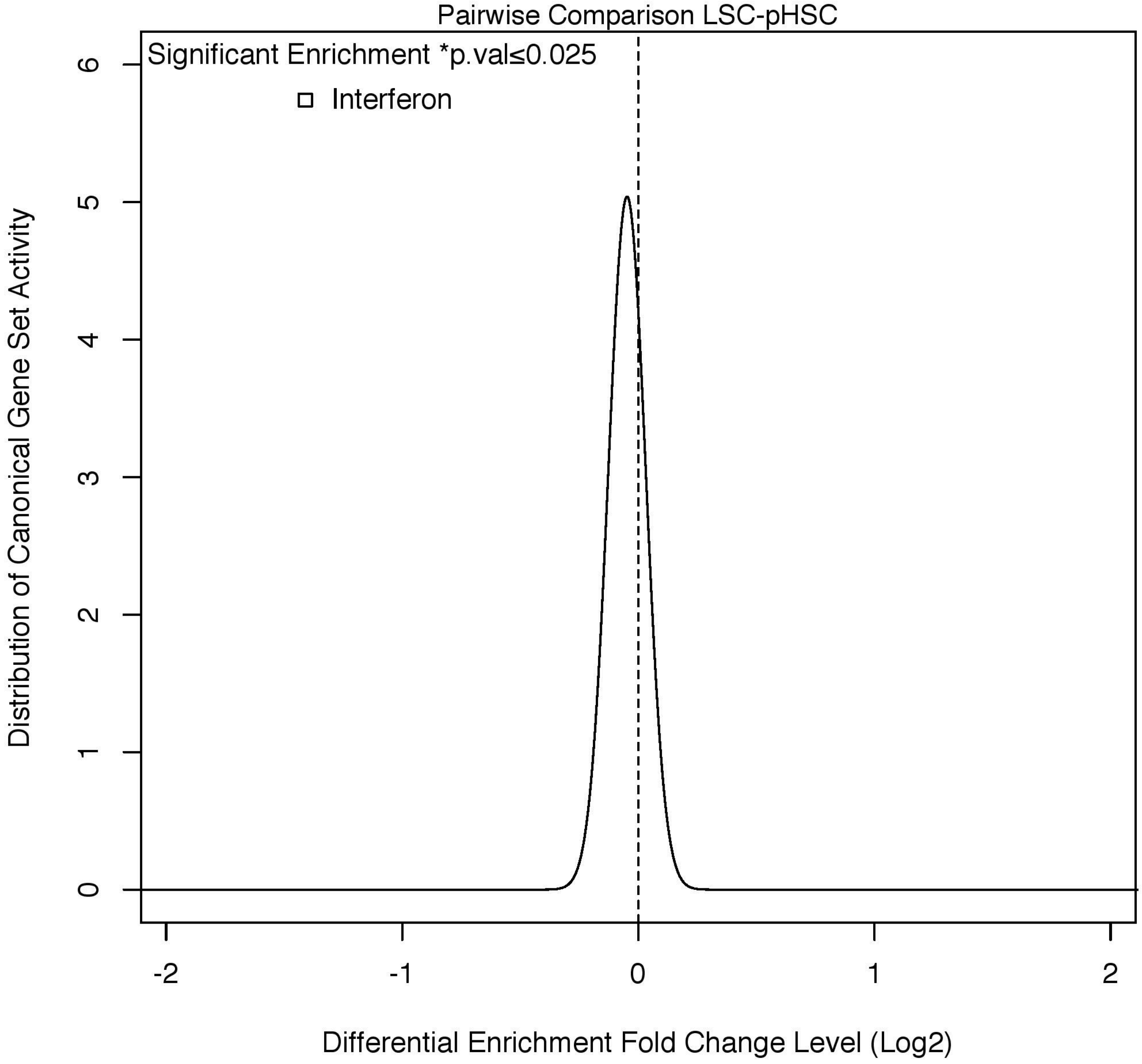

Similar labeling to Figure2B, with instead comparing LSC-pHSC using merged interferon pathways.

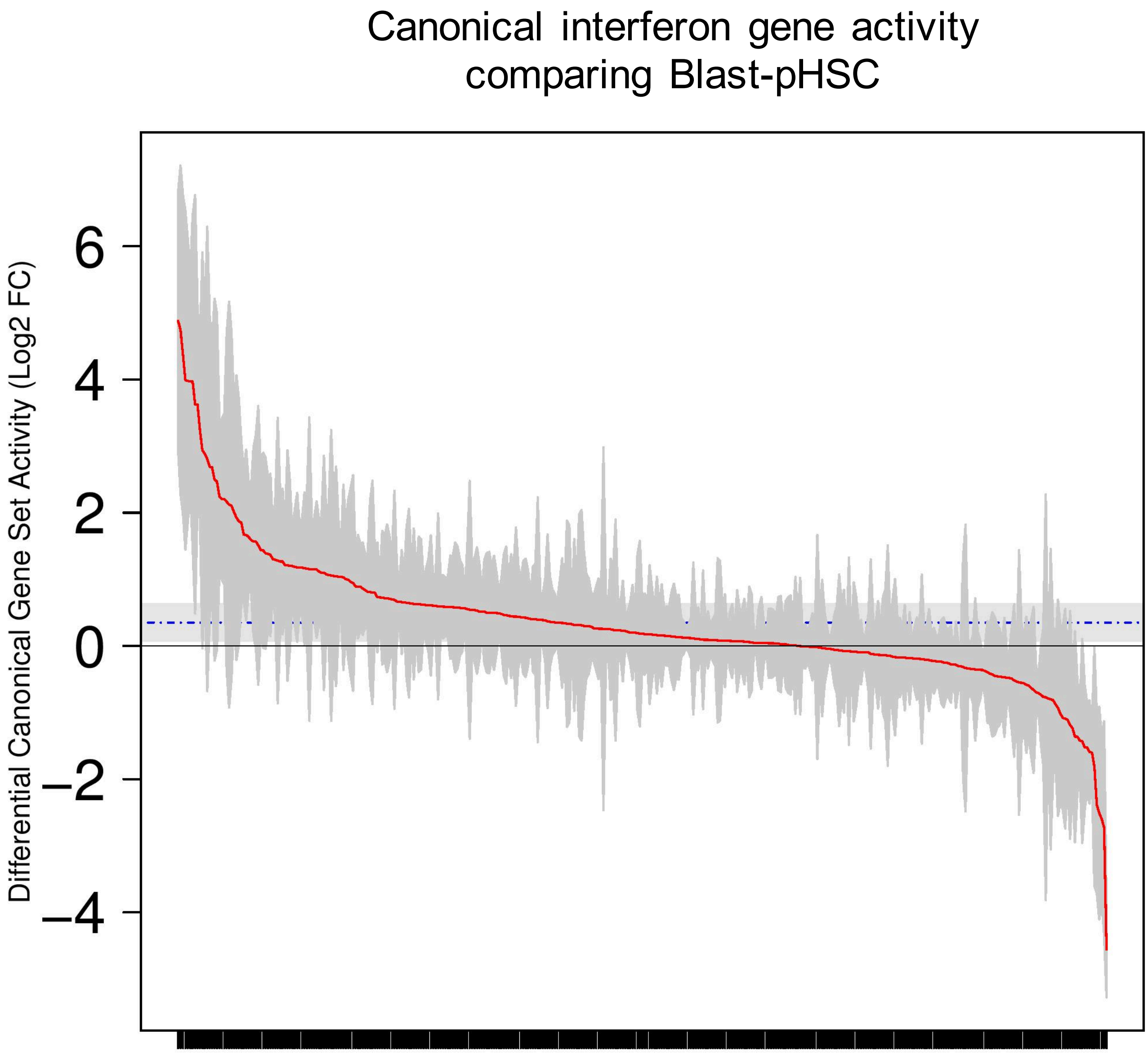

Labeling is similar to Supplement 3A, however with respect to Blast-pHSC comparison.

Blast-pHSC (GO) MsigDB interferon gene-set analysis

Blast-pHSC (GO) Interferon MSigDB	log.FC	p.Value	FDR
GO_REGULATION_OF_INTERFERON_BETA_PRODUCTION	0.6349	1.56E-05	0.0005
GO_POSITIVE_REGULATION_OF_INTERFERON_BETA_PRODUCTION	0.6272	6.79E-05	0.0007
GO_REGULATION_OF_INTERFERON_ALPHA_PRODUCTION	0.9260	7.25E-05	0.0007
GO_NEGATIVE_REGULATION_OF_TYPE_I_INTERFERON_PRODUCTION	0.4088	0.0001	0.0011
GO_POSITIVE_REGULATION_OF_INTERFERON_ALPHA_PRODUCTION	0.8089	0.0002	0.0011
GO_REGULATION_OF_TYPE_I_INTERFERON_PRODUCTION	0.4396	0.0006	0.0027
GO_POSITIVE_REGULATION_OF_TYPE_I_INTERFERON_PRODUCTION	0.4396	0.0006	0.0027
REGULATION_OF_INTERFERON_GAMMA_BIOSYNTHETIC_PROCESS	0.7919	0.0040	0.0139
INTERFERON_GAMMA_BIOSYNTHETIC_PROCESS	0.7317	0.0045	0.0139
GO_TYPE_I_INTERFERON_RECEPTOR_BINDING	0.4163	0.0046	0.0139
GO_CELLULAR_RESPONSE_TO_INTERFERON_GAMMA	0.5614	0.0065	0.0174
INTERFERON_GAMMA_PRODUCTION	0.5683	0.0073	0.0174
GO_RESPONSE_TO_INTERFERON_GAMMA	0.5402	0.0075	0.0174
GO_INTERFERON_GAMMA_MEDIATED_SIGNALING_PATHWAY	0.6815	0.0090	0.0192
INTERFERON (Combined Gene Set)	0.3495	0.0115	0.0221
GO_REGULATION_OF_INTERFERON_GAMMA_BIOSYNTHETIC_PROCESS	0.6695	0.0118	0.0221
GO_POSITIVE_REGULATION_OF_INTERFERON_GAMMA_BIOSYNTHETIC_PROCESS	0.6152	0.0154	0.0271
GO_POSITIVE_REGULATION_OF_INTERFERON_GAMMA_PRODUCTION	0.4869	0.0166	0.0277
GO_RESPONSE_TO_TYPE_I_INTERFERON	0.3904	0.0325	0.0499
GO_NEGATIVE_REGULATION_OF_INTERFERON_GAMMA_PRODUCTION	0.3479	0.0332	0.0499
GO_REGULATION_OF_TYPE_I_INTERFERON_MEDIATED_SIGNALING_PATHWAY	0.2357	0.0394	0.0563
GO_REGULATION_OF_INTERFERON_GAMMA_PRODUCTION	0.3529	0.0575	0.0785
HEMATOPOIETIN_INTERFERON_CLASSD200_DOMAIN_CYTOKINE_RECEPTOR_ACTIVITY	0.3666	0.0958	0.1250
GO_REGULATION_OF_INTERFERON_GAMMA_SECRETION	0.2943	0.1027	0.1284
GO_REGULATION_OF_RESPONSE_TO_INTERFERON_GAMMA	0.1805	0.1495	0.1794
HEMATOPOIETIN_INTERFERON_CLASSD200_DOMAIN_CYTOKINE_RECEPTOR_BINDING	0.1590	0.2544	0.2935
GO_CELLULAR_RESPONSE_TO_INTERFERON_BETA	0.0555	0.5439	0.6043
GO_INTERFERON_GAMMA_PRODUCTION	-0.0912	0.6011	0.6441
GO_RESPONSE_TO_INTERFERON_BETA	0.0515	0.7229	0.7478
GO_RESPONSE_TO_INTERFERON_ALPHA	0.0120	0.9437	0.9437

Labeling similar to Figure 2A. Lists all pathways related to interferon using GO comparing Blast-pHSC.

Interferon pathway distribution plot comparing Blast-pHSC

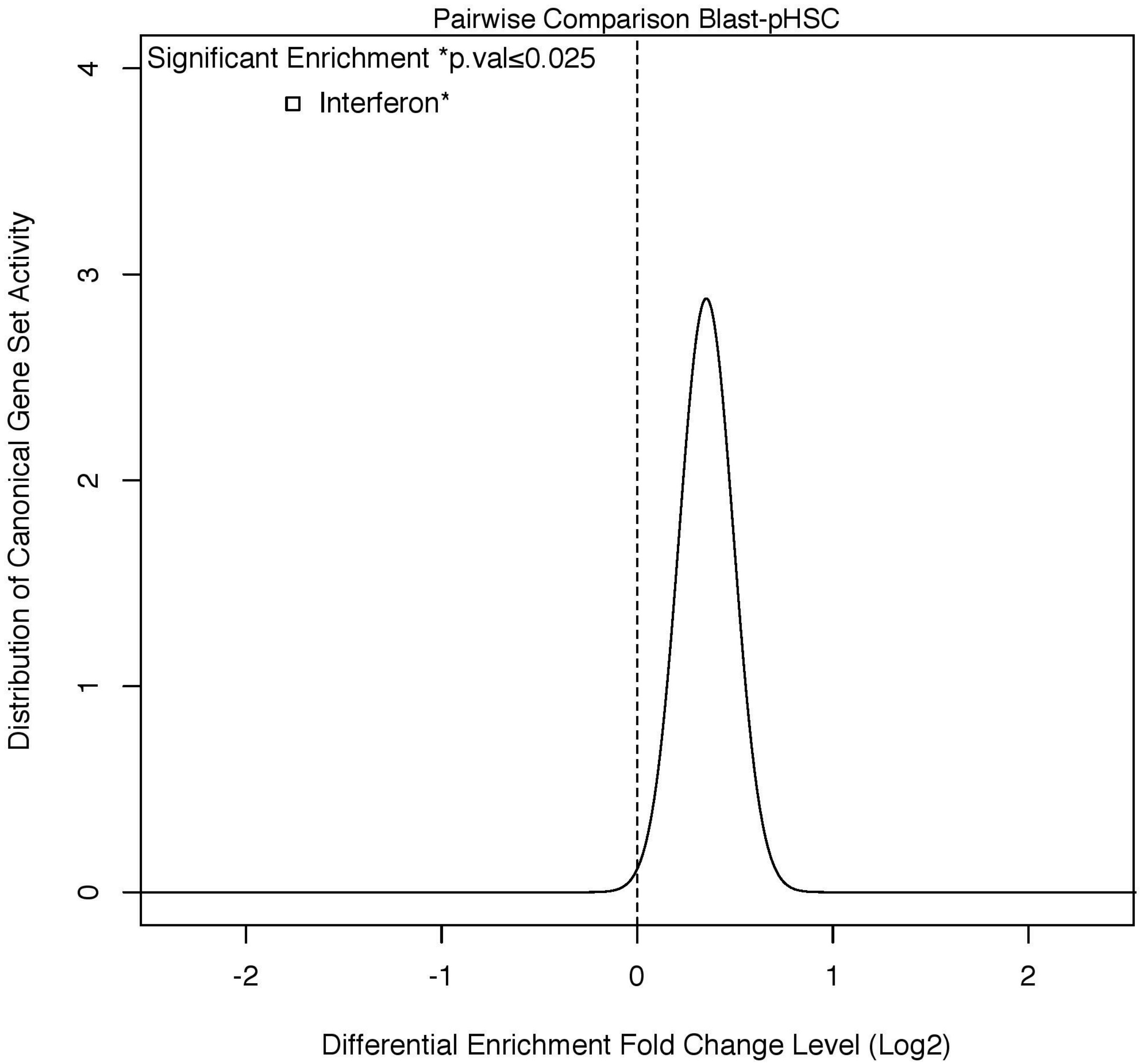

Similar labeling to FIgure2B, with instead comparing Blast-pHSC using merged interferon pathways. Note that positive fold change indicates higher gene activity in Blasts compared with pHSC.

b

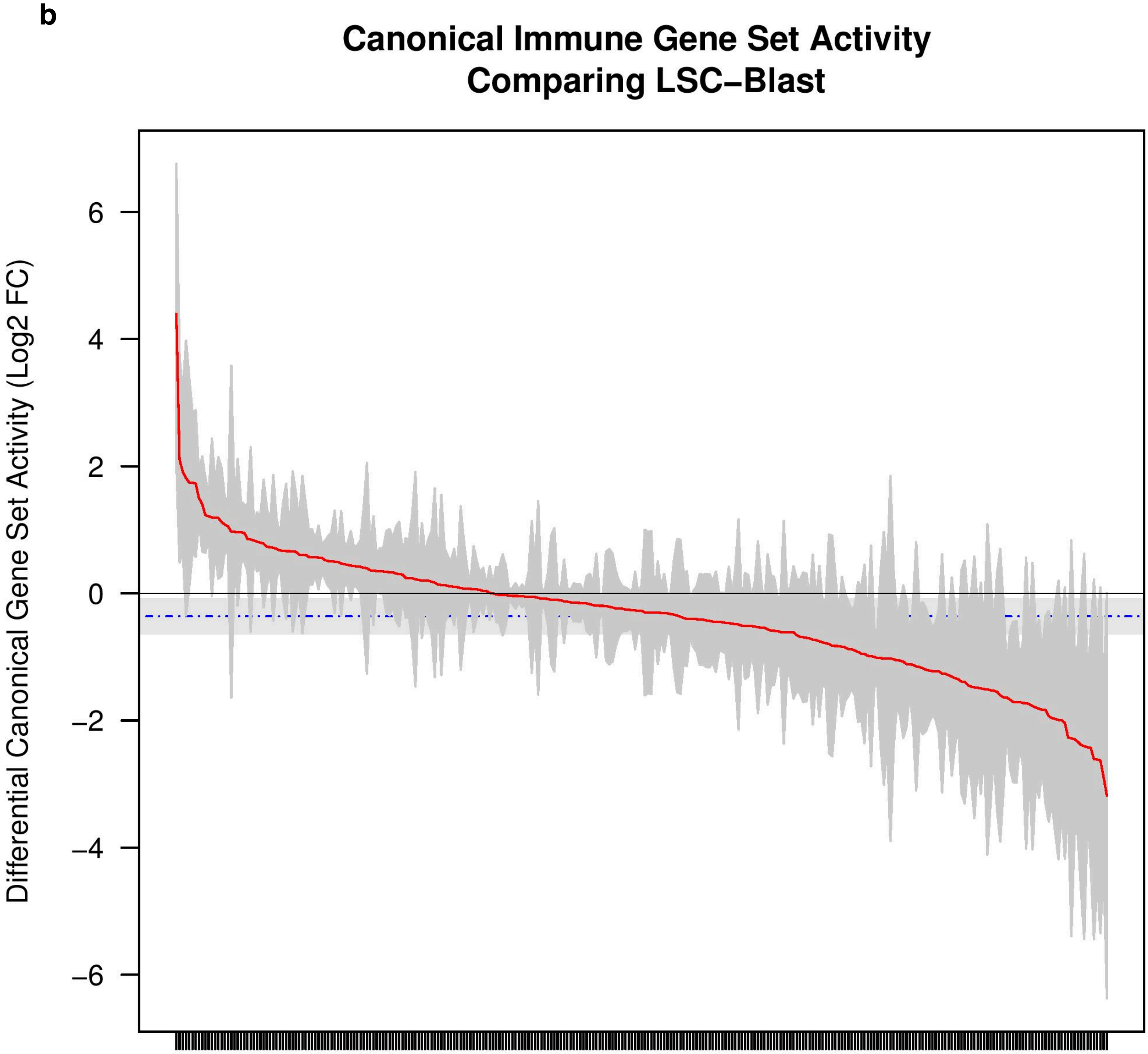

The Immune gene set contained 335 unique genes from Gene Ontology (MSigDB). Labeling is similar to supplement 3A, except with respect to Immune related genes comparing LSC-Blasts.

LSC-Blast (GO) MsigDB immune gene-set analysis *p.value <0.05

LSC-Blast (GO) MsigDB	log.fold.change	p.Value	FDR
Acute_inflammatory_response*	-0.715477207	0.002988949	0.015542534
Adaptive_immune_response_go_0002460*	-0.233370676	0.048202648	0.083551257
Go_acute_inflammatory_response	-0.448684976	0.051492631	0.083675526
Go_inflammatory_response*	-0.416391038	0.007434892	0.027147568
Go_positive_regulation_of_inflammatory_response**	-0.343401217	0.030273235	0.065592008
Go_regulation_of_inflammatory_response*	-0.400964389	0.002144159	0.013937035
Humoral_immune_response	-0.393832605	0.073948014	0.100477695
Immune (Combined Set 335 Genes)*	-0.359771838	0.009397235	0.027147568
Immune_effector_process	-0.221684644	0.077600938	0.100477695
Immune_response*	-0.376581228	0.027990005	0.065592008
Immune_system_development*	-0.384975163	0.000269815	0.004633724
Immune_system_process**	-0.361182328	0.00917365	0.027147568
Inflammation(Combined Set of 649 Genes)*	-0.385519405	0.003741098	0.016211424
Inflammatory_response*	-0.48623259	0.023216033	0.060361685
Innate_immune_response	-0.371927548	0.066354713	0.095845697
Positive_regulation_of_immune_response*	-0.328325557	0.042252733	0.078469361
Positive_regulation_of_immune_system_process*	-0.280325294	0.00057216	0.004958722
Production_of_molecular_mediator_of_immune_response	-0.270328872	0.061532455	0.09410846
Regulation_of_immune_response*	-0.304748406	0.035090761	0.070181522
Regulation_of_immune_system_process*	-0.258362438	0.00035644	0.004633724

All pathways used in the depiction of Figure 2B LSC-Blast.

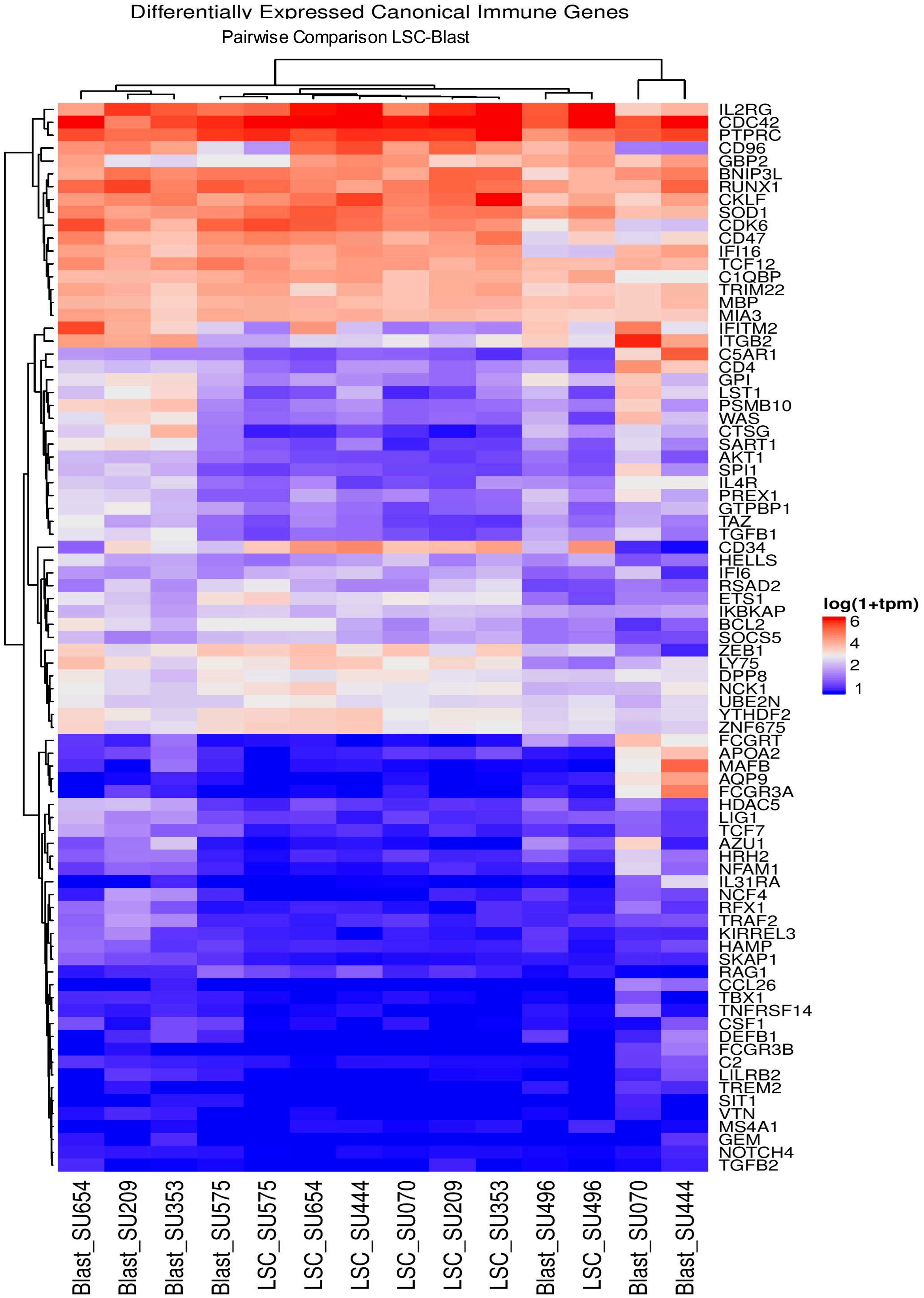

Similar labeling to supplement 3A.

c

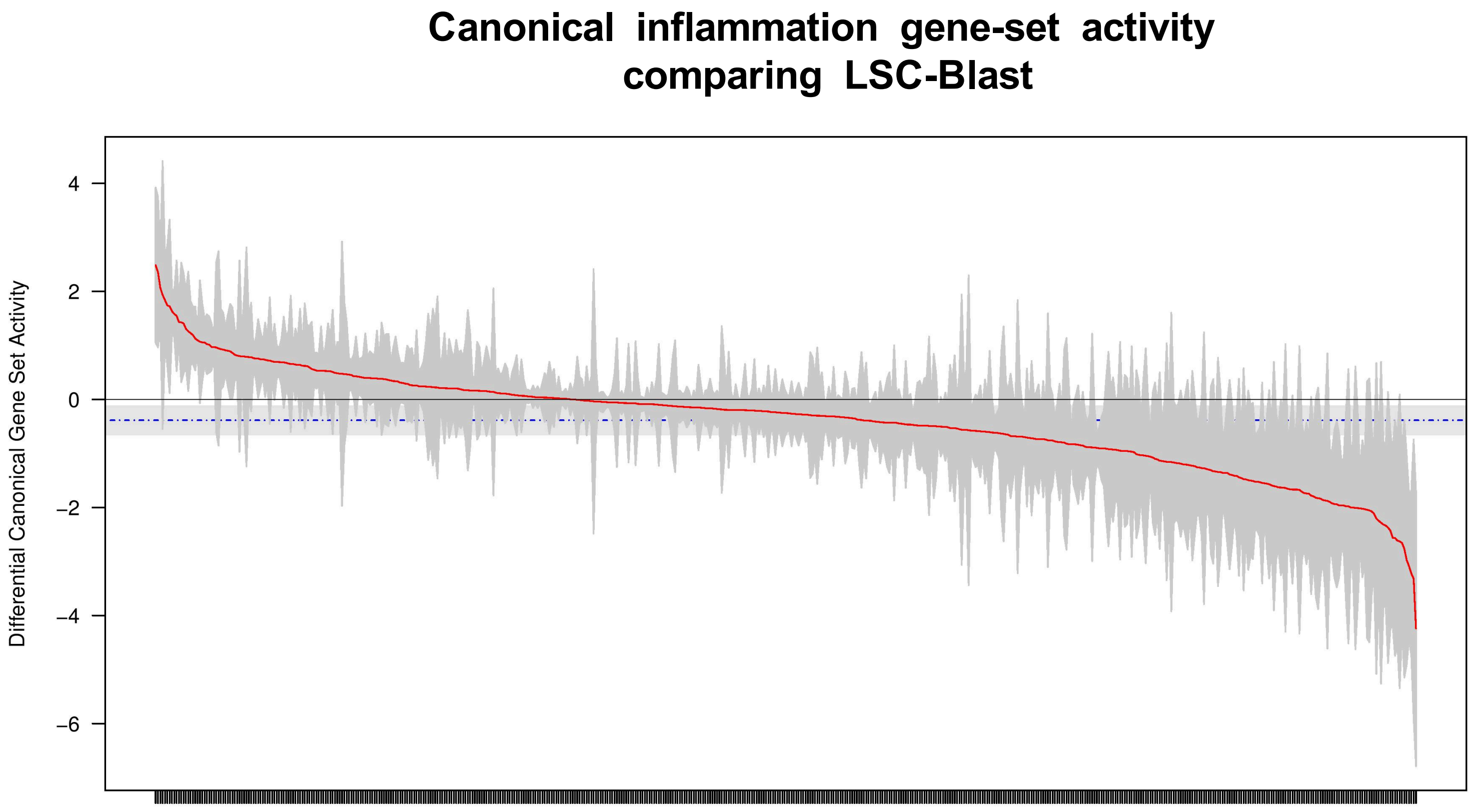

Labeling is similar to Supplemental Figure Supplement 3 A. The Inflammation Gene set merged GO (MSigDB) Inflammation Response and Acute Inflammation Response totaling 649 unique genes.

The dashed blue line is below 0 on y-axis indicating that the gene-set enrichment is down-activated in the comparison of LSC-Blast, indicating higher activation in Blast, and less activation in LSC.

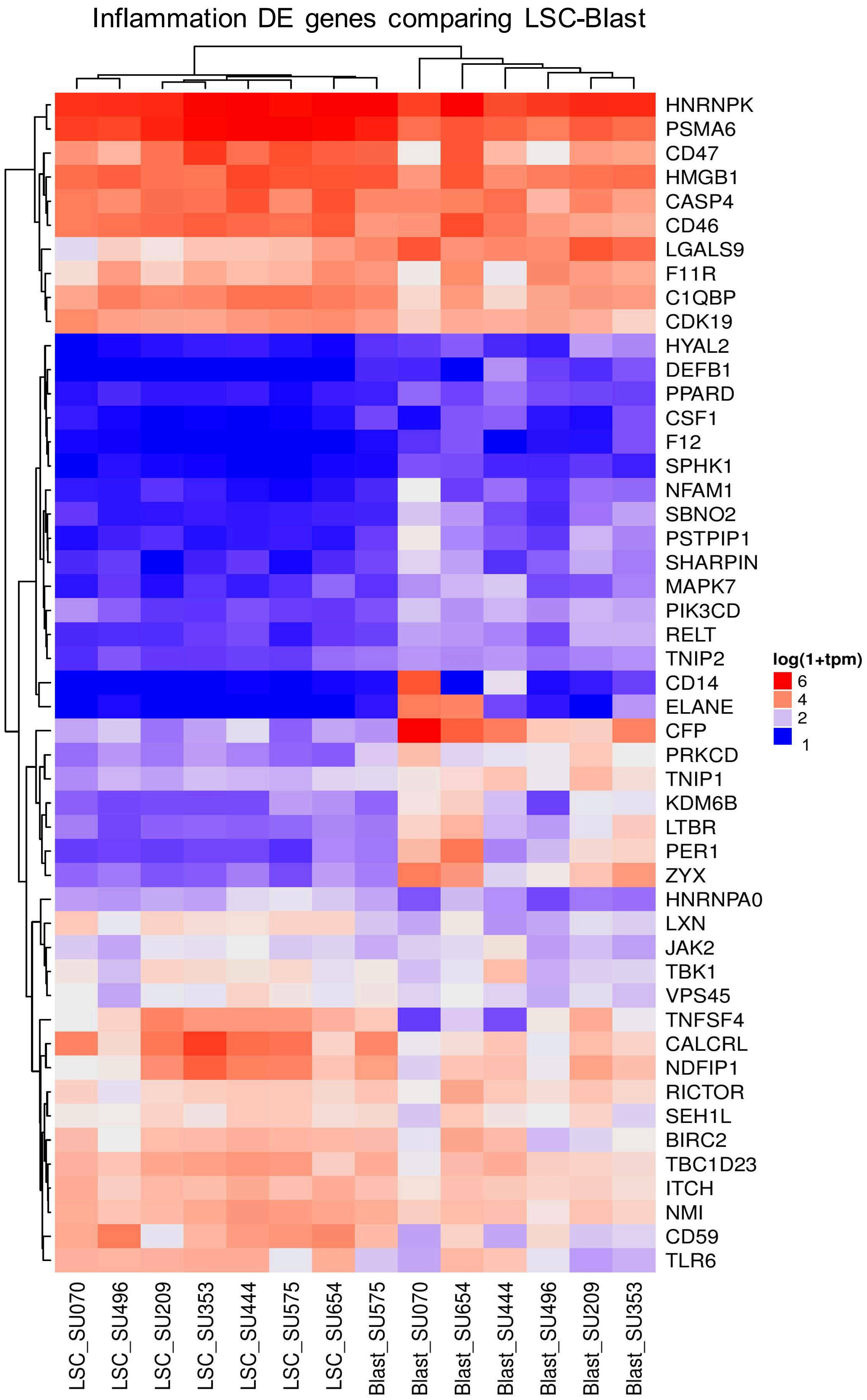

Inflammation DE genes comparing LSC-Blast
HNRNPK
PSMA6
CD47
HMGB1
CASP4
CD46
LGALS9
F11R
C1QBP
CDK19
HYAL2
DEFB1
PPARD
CSF1
F12
SPHK1
NFAM1
SBNO2
PSTPIP1
SHARPIN
MAPK7
PIK3CD
RELT
TNIP2
CD14
ELANE
CFP
PRKCD
TNIP1
KDM6B
LTBR
PER1
ZYX
HNRNPA0
LXN
JAK2
TBK1
VPS45
TNFSF4
CALCRL
NDFIP1
RICTOR
SEH1L
BIRC2
TBC1D23
ITCH
NMI
CD59
TLR6
log(1+tpm)
6
4
2
1
LSC_SU070
LSC_SU496
LSC_SU209
LSC_SU353
LSC_SU444
LSC_SU575
LSC_SU654
Blast_SU575
Blast_SU070
Blast_SU654
Blast_SU444
Blast_SU496
Blast_SU209
Blast_SU353

D

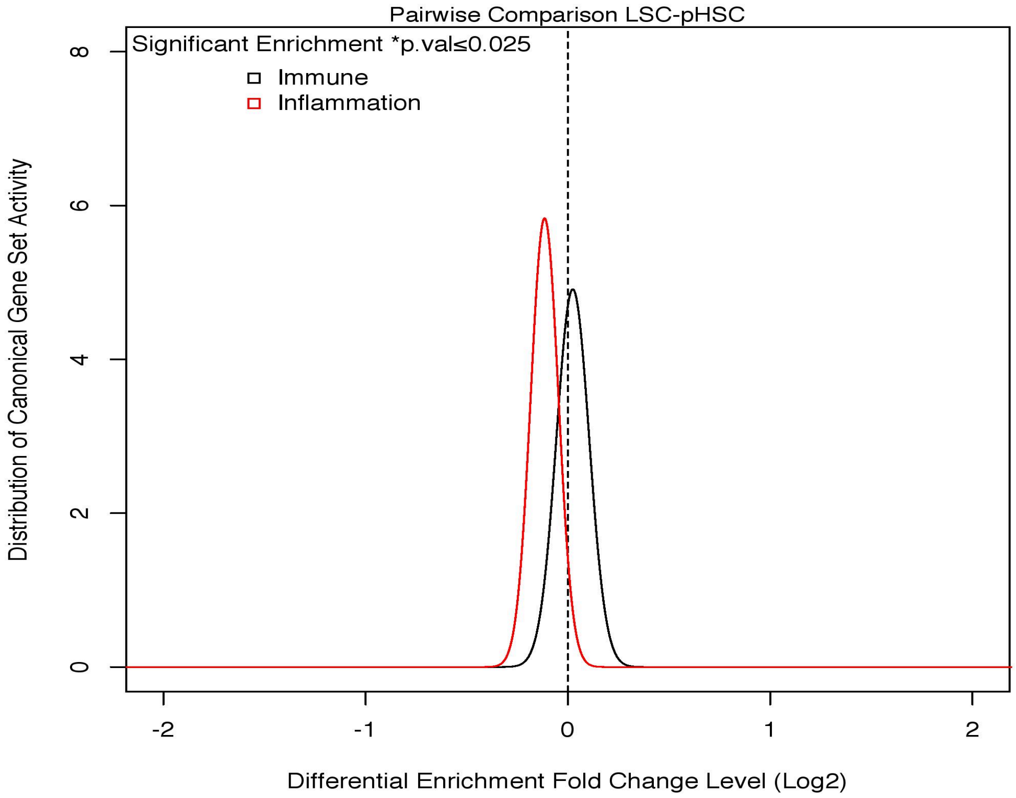

pathway name (GO) comparing LSC-pHSC	log.fold.change	p.Value	FDR
IMMUNE_SYSTEM_DEVELOPMENT	-0.363025839	2.76E-05	0.000717492
NEGATIVE_REGULATION_OF_IMMUNE_SYSTEM_PROCESS	0.270310336	0.000421516	0.005479707
ACUTE_INFLAMMATORY_RESPONSE	-0.435721735	0.001916285	0.016607804
IMMUNE_EFFECTOR_PROCESS	0.208150501	0.015038204	0.094661475
ACTIVATION_OF_IMMUNE_RESPONSE	0.214794502	0.020557639	0.094661475
GO_POSITIVE_REGULATION_OF_ACUTE_INFLAMMATORY_R	-0.349410172	0.021844956	0.094661475
ADAPTIVE_IMMUNE_RESPONSE	0.227708231	0.050741534	0.188468555
REGULATION_OF_IMMUNE_EFFECTOR_PROCESS	0.212546161	0.063032186	0.204854604
PRODUCTION_OF_MOLECULAR_MEDIATOR_OF_IMMUNE_RI	0.18474162	0.075459193	0.211389622
GO_REGULATION_OF_INFLAMMATORY_RESPONSE	-0.128458501	0.083622649	0.211389622
Inflammation (Combined Gene Set)	-0.115373033	0.089434071	0.211389622
ADAPTIVE_IMMUNE_RESPONSE_GO_0002460	0.188786783	0.131683899	0.285315113
INFLAMMATORY_RESPONSE	0.124253996	0.233715173	0.467430346
GO_INFLAMMATORY_RESPONSE	-0.07316779	0.298146163	0.539957568
GO_ACUTE_INFLAMMATORY_RESPONSE	0.087637491	0.322237068	0.539957568
IMMUNOLOGICAL_SYNAPSE	0.133482169	0.343769164	0.539957568
IMMUNE_RESPONSE	0.078024002	0.367474629	0.539957568
REGULATION_OF_IMMUNE_SYSTEM_PROCESS	0.069405234	0.373816778	0.539957568
POSITIVE_REGULATION_OF_IMMUNE_SYSTEM_PROCESS	0.060968393	0.454206761	0.621546094
POSITIVE_REGULATION_OF_IMMUNE_RESPONSE	0.047898367	0.573749046	0.74587376
Immune (Combined Gene Set)	0.02	0.77771	0.77771

Labeling similar to LSC.vs.Blast, instead comparing LSC.vs.pHSC. The genes in the merged immune and inflammation were tested with an adjusted significance threshold accounting for pairwise comparisons signified by 'adj.p.value'. The x-axis represent log2 Fold change comparing LSC-pHSC, so negative FC indicates negative gene-set activity indicating that the gene-set enrichment is down-activated in the comparison of LSC-pHSC, indicating higher activation in pHSC, and less activation in LSC.

The table shows all the individual sets used in pooling the gene set into a combined gene set for Immune and Inflammation.

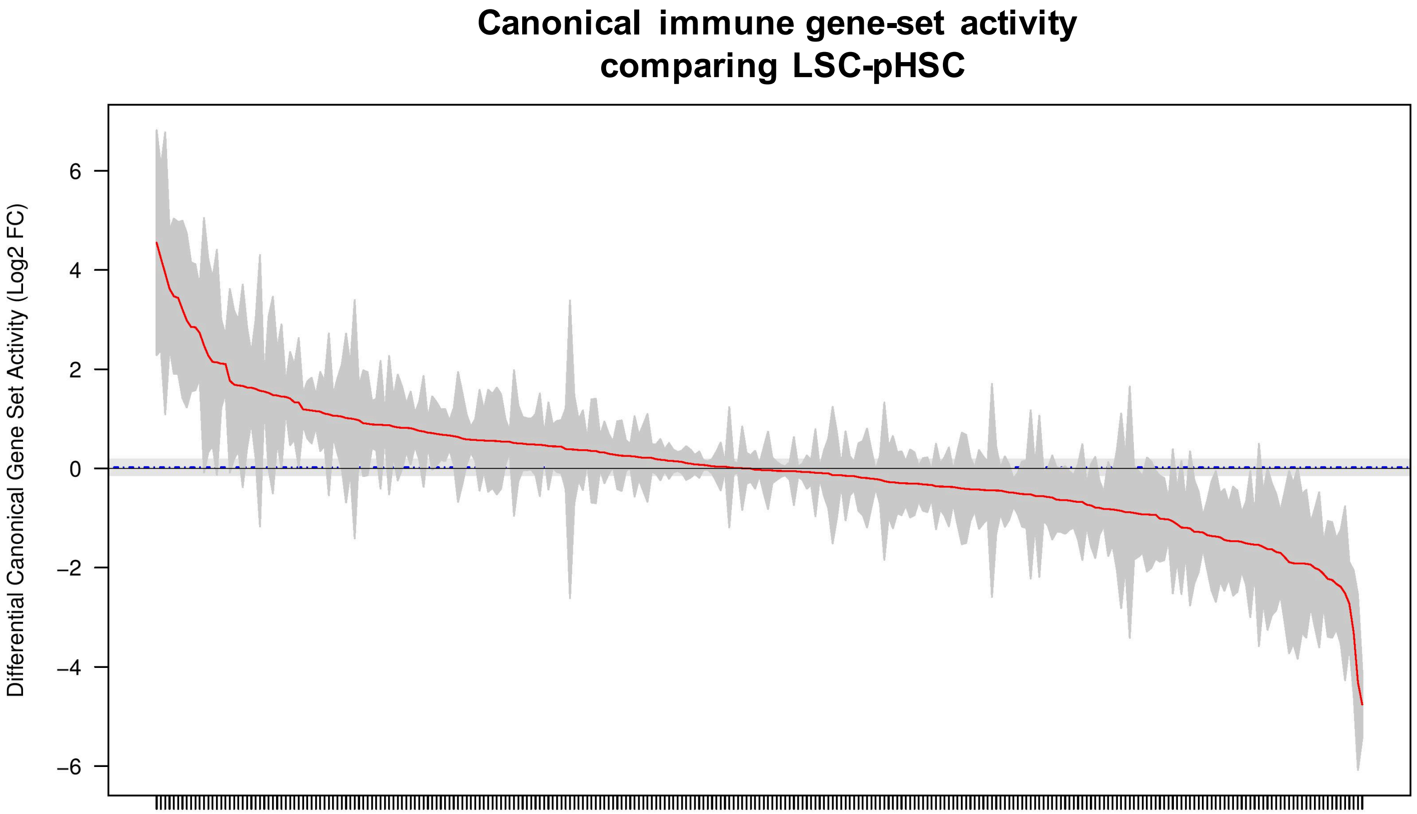

Labeling similar to Supplement 3A, instead comparing LSC-pHSC

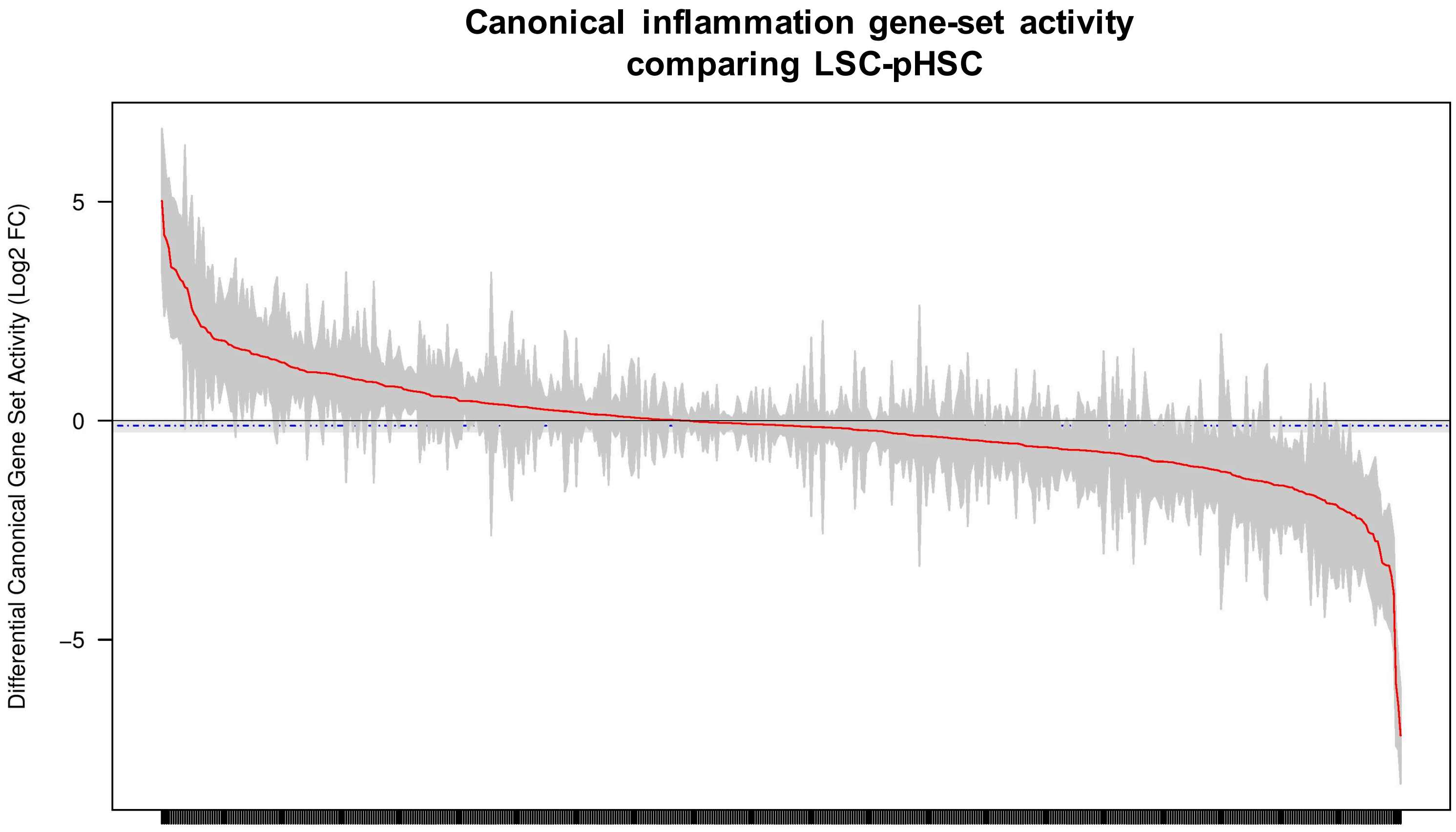

Labeling similar to Supplement 3A, instead comparing LSC-pHSC

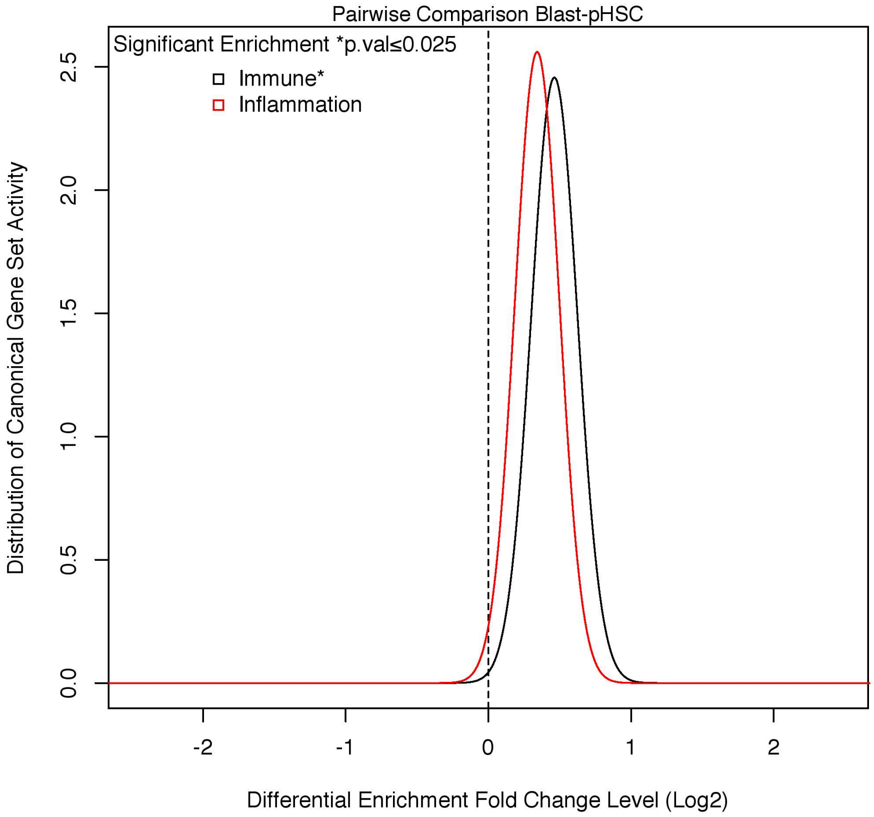

pathway.name	log.FC	p.Value	FDR
NEGATIVE_REGULATION_OF_IMMUNE_SYSTEM_PROCESS	0.345029409	4.08E-05	0.001061892
REGULATION_OF_IMMUNE_SYSTEM_PROCESS	0.401525965	0.000194131	0.002523709
ACTIVATION_OF_IMMUNE_RESPONSE	0.385231042	0.000408966	0.003544371
POSITIVE_REGULATION_OF_IMMUNE_SYSTEM_PROCESS	0.406228666	0.000644203	0.004187323
PRODUCTION_OF_MOLECULAR_MEDIATOR_OF_IMMUNE_RESPONSE	0.519970365	0.001822851	0.009478826
ADAPTIVE_IMMUNE_RESPONSE	0.514550025	0.003041695	0.013180677
REGULATION_OF_IMMUNE_EFFECTOR_PROCESS	0.284511317	0.004369008	0.013193374
IMMUNE_SYSTEM_PROCESS	0.467195131	0.004640507	0.013193374
Immune* (Combined Gene Set)	0.464833852	0.004844822	0.013193374
IMMUNE_EFFECTOR_PROCESS	0.500211628	0.005074375	0.013193374
ADAPTIVE_IMMUNE_RESPONSE_GO_0002460	0.499458961	0.005781257	0.01366479
IMMUNE_RESPONSE	0.532445939	0.007571309	0.016404503
INFLAMMATORY_RESPONSE	0.671634321	0.009993819	0.019987639
GO_REGULATION_OF_INFLAMMATORY_RESPONSE	0.343392516	0.01813929	0.033687253
HUMORAL_IMMUNE_RESPONSE	0.479479048	0.021116113	0.03436943
IMMUNOLOGICAL_SYNAPSE	0.322194804	0.021150419	0.03436943
GO_INFLAMMATORY_RESPONSE	0.413905194	0.022659301	0.034472068
POSITIVE_REGULATION_OF_IMMUNE_RESPONSE	0.429813953	0.024032308	0.034472068
REGULATION_OF_IMMUNE_RESPONSE	0.39957518	0.02523217	0.034472068
GO_ACUTE_INFLAMMATORY_RESPONSE	0.606810691	0.026516976	0.034472068
Inflammation (Combined Gene Set)	0.342	0.029457	0.029457

Labeling similar to LSC.vs.Blast, instead comparing Blast.vs.pHSC. The x-axis represent log2 Fold change comparing Blast-pHSC, so positive FC indicates positive gene-set activity indicating that the gene-set enrichment is up-activated in the comparison of Blast-pHSC, indicating higher activation in Blast, and less activation in pHSC.

The table shows all the individual sets used in pooling the gene set into a combined gene set for Immune and Inflammation.

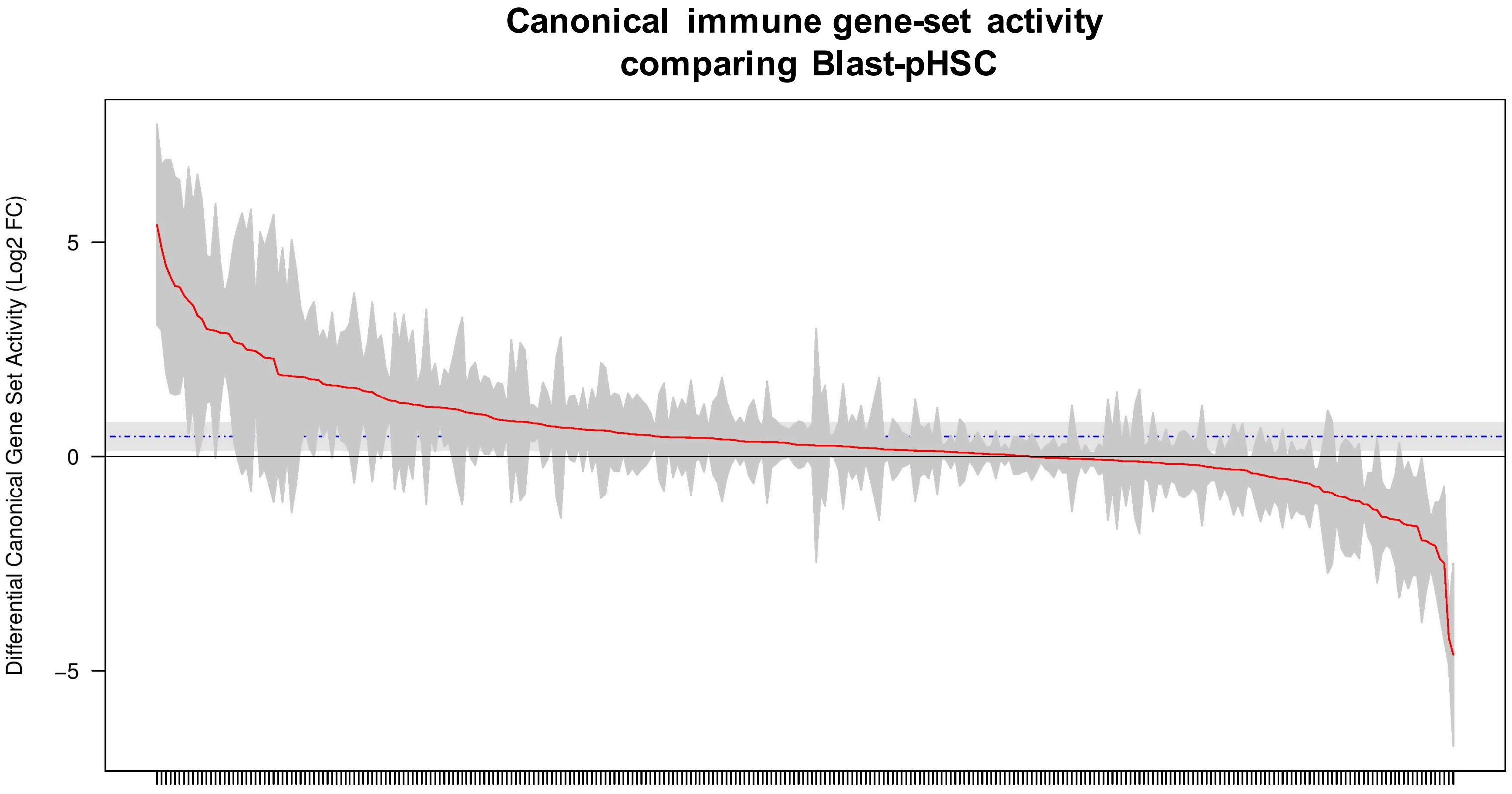

Labeling similar to Supplement 3A, instead comparing Blast-pHSC in the Immune gene-set.

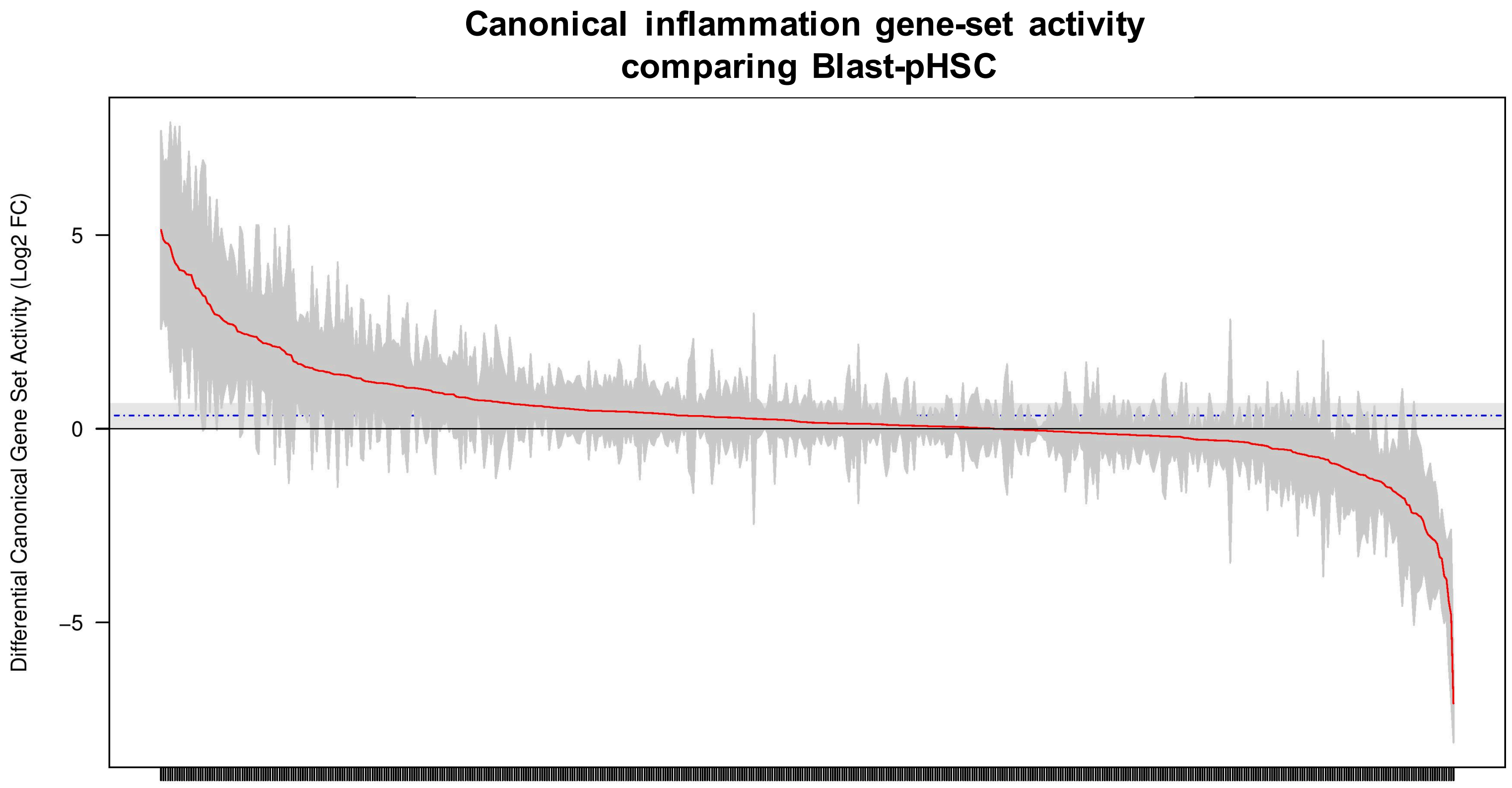

Labeling similar to Supplement 3A, instead comparing Blast-pHSC with respect to inflammation gene-sets.

Supplement figure 4

a

EVI-1 expression per clonal stage

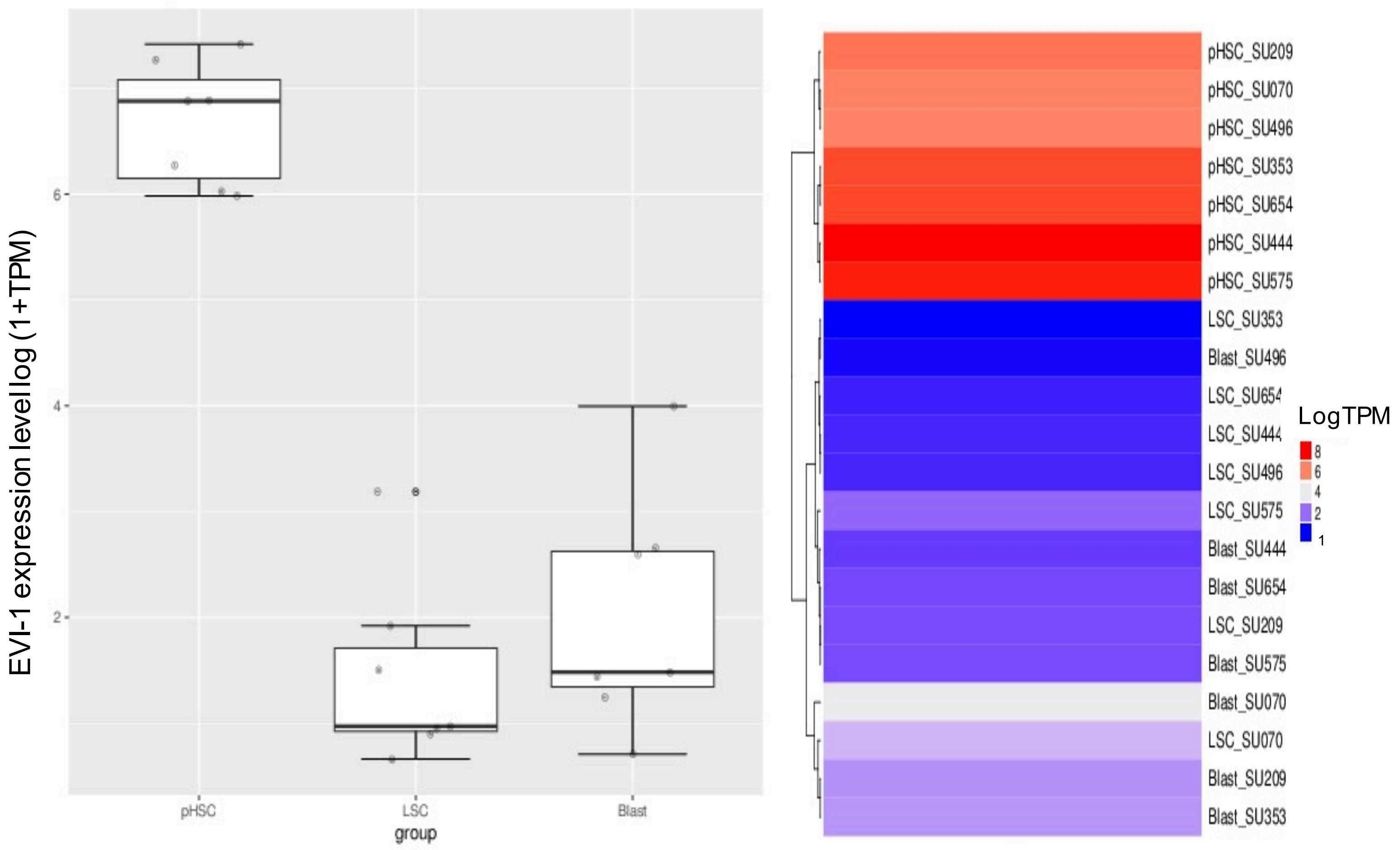

EVI-1 expression

ANOVA of pHSC, LSC, and Blast EVI-1 expression

ANOVA	pHSC vs. LSC	Blast vs. LSC	Blast vs. pHSC
2.40E-09	6.13E-09	0.727607	3.74E-08

The EVI-1 Expression was analyzed across groups using an ANOVA that included pairwise-t-tests which used Bonferroni multiple testing corrections to adjust the significance threshold. The log base 10 (1+TPM) of the EVI-1 expression in each group is displayed in the box plot and as a heatmap.

b

GO Biological Processes Pathways comparing Blast vs. pHSC	log.FC	p.value	FDR
GO Regulation of NIK NF Kappa-Beta Signaling*	0.392	1.04E-05	6.22E-05
GO Positive Regulation of NIK NF Kappa-Beta Signaling*	0.4496	3.58E-05	0.0001
**NFKB Combined Gene-Set***	0.314	0.0006	0.0018
GO Activation of NF Kappa-Beta Inducing Kinase Activity*	0.354	0.0018	0.0027
GO NIK NF Kappa-Beta Signaling*	0.294	0.0022	0.0027
GO Regulation of NF Kappa-Beta Import Into Nucleus*	0.3491	0.0023	0.0027
GO Positive Regulation of NF Kappa-Beta Import Nucleus	0.2821	0.0333	0.0333
GO Positive Regulation of NF Kappa-Beta Transcription Factor Activity*	0.3230	0.0066	0.0076

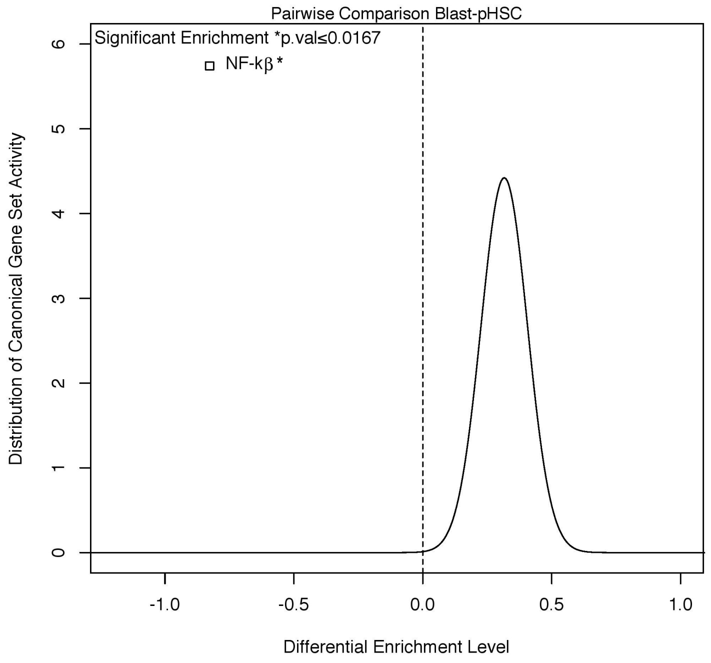

GO Biological Processes Pathways comparing LSC vs. Blast	log.FC	p.Value	FDR
GO Positive Regulation of NF Kappa-Beta Transcription Factor Activity*	-0.298	0.003	0.022
GO Positive Regulation of NF Kappa-Beta Import Into Nucleus*	-0.346	0.006	0.022
GO Regulation of NF Kappa-Beta Import Into Nucleus*	-0.269	0.014	0.037
**NFKB Combined Gene-Set**	-0.177	0.0408	0.082
GO Regulation of NIK NF Kappa-Beta Signaling	-0.143	0.060	0.095
GO Positive Regulation of NIK NF Kappa-Beta Signaling	-0.112	0.244	0.325
GO Activation of NF Kappa-Beta Inducing Kinase Activity	-0.046	0.661	0.734
GO NIK NF Kappa-Beta Signaling	-0.033	0.734	0.734

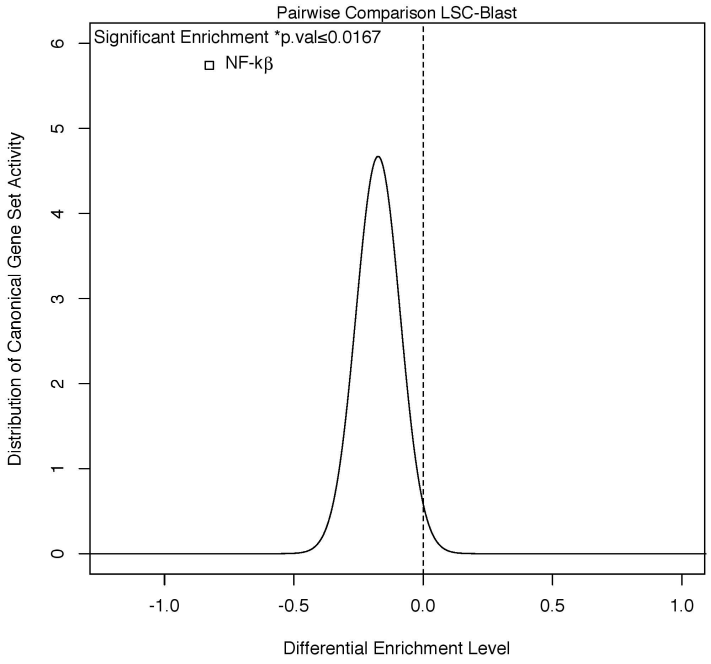

EVI-1 has been shown to suppress the NF-kB pathway in AML, so we tested the MSigDB biological processes pathways for 7 of positive regulation of NF-kB pathways. We also combined all 7 gene pathways into a combined gene set of 247 unique genes in 7 of the canonical MSigDB NF-kB pathways used. The analysis shown compared Blast-pHSC, LSC-Blast, and LSC-pHSC respectively. The x-axis is the differential activity level (log2 FC) where x>0 indicates positive activation in the comparison group.

The p-value threshold was adjusted for 3 pairwise comparisons with a threshold of 0.0167 (where an asterisk (*) denotes p.value ≤0.0167).

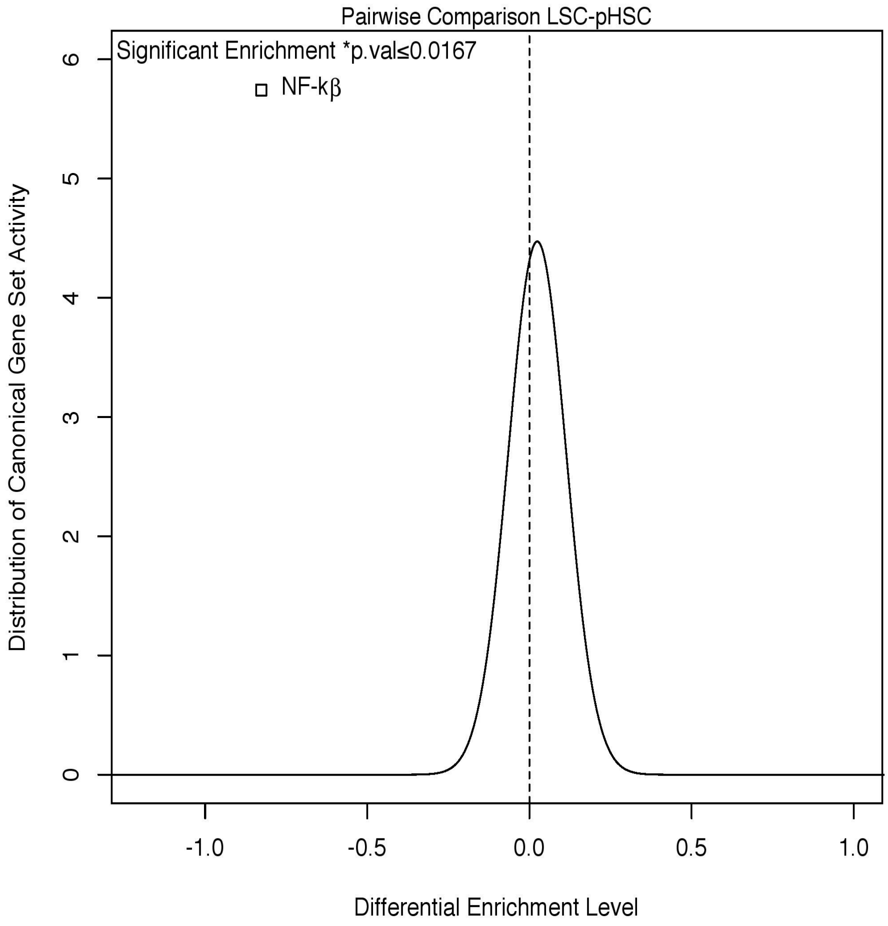

GO Biological Processes Pathways comparing LSC vs. pHSC	log.FC	p.value	FDR
GO Activation of NF Kappa-Beta Inducing Kinase Activity*	0.219136	0.007914	0.045008779
GO Positive Regulation of NIK NF Kappa-Beta Signaling*	0.222418	0.011252	0.045008779
GO Regulation of NIK NF Kappa-Beta Signaling	0.125693	0.058721	0.156588793
GO Positive Regulation of NF Kappa-Beta Import Into Nucleus	-0.14245	0.129881	0.259761951
GO NIK NF Kappa-Beta Signaling	0.136359	0.349913	0.544957721
GO Positive Regulation of NF Kappa-Beta Transcription Factor Activity	-0.08236	0.408718	0.544957721
**NFKB Combined Gene-Set**	0.025248	0.797416	0.848986437
GO Regulation of NF Kappa-Beta Import Into Nucleus	-0.01569	0.848986	0.848986437

Supplement figure 5

Module 5 predictive gene expression

a

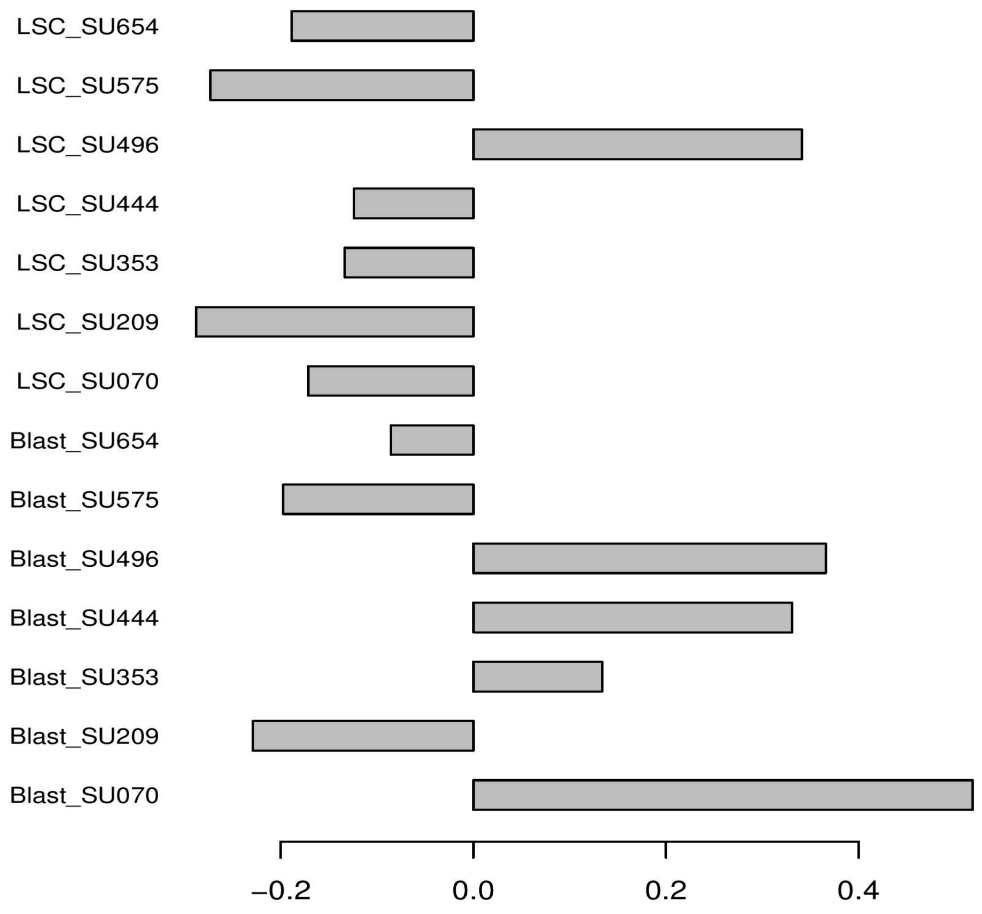

Depicts 'Eigen-Gene' (Module) Number 5 Expression Levels For Each Sample Group, The Module Number is located in the title, which corresponds to the 5th row in the main Figure 4.A. The y-axis denotes the sample group along with the unique patient identifier. The x-axis denotes the predictive expression levels, where the positive/negative x-axis values denotes as to whether a sample is likely to be high/low expressed in that given module respectively.

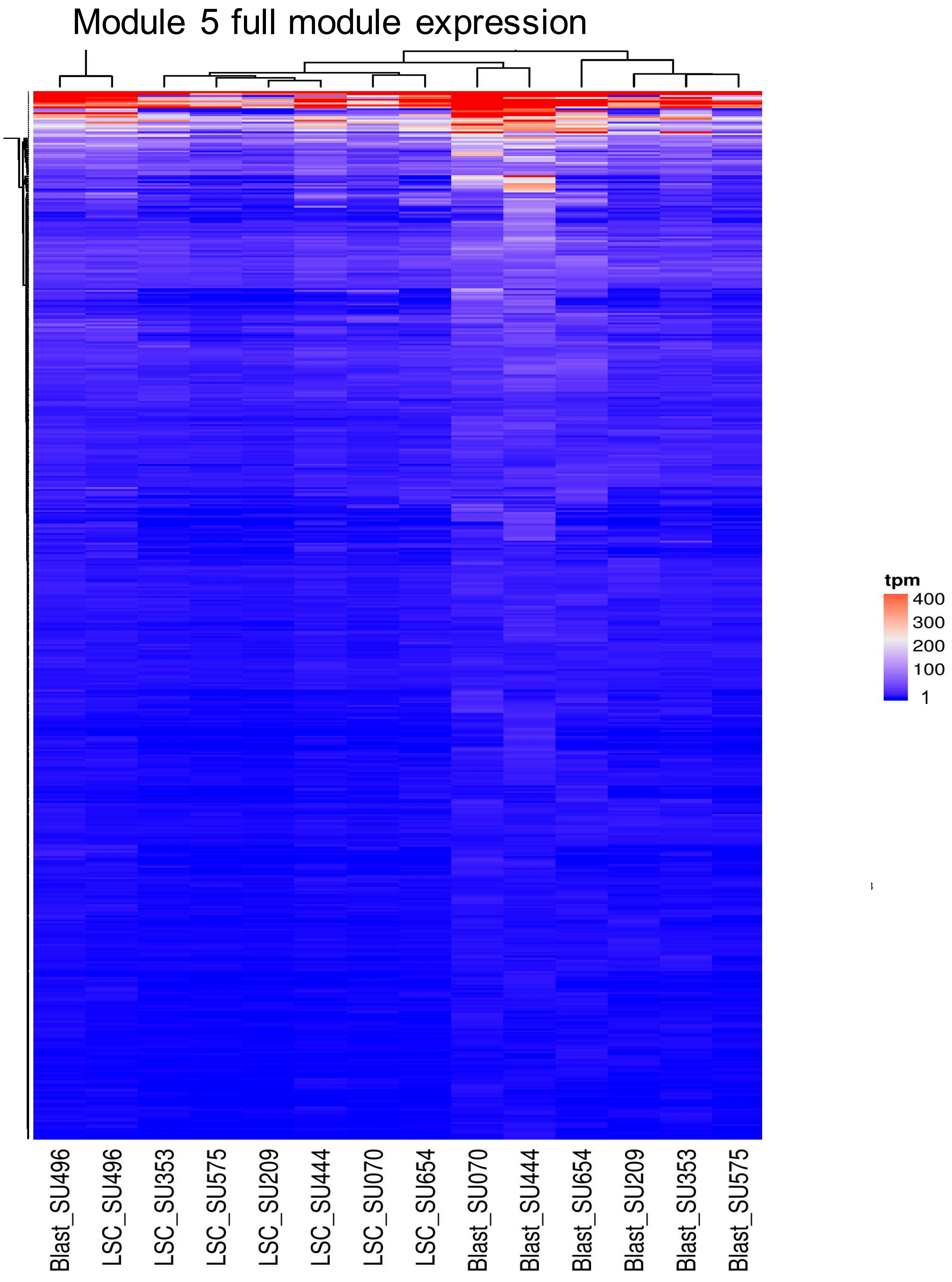

Depicts the entire module gene expression. The x-axis denotes each sample and patient identifier. The y-axis denotes the expression of each gene in module/hub 5, where the expression values are in TPM.

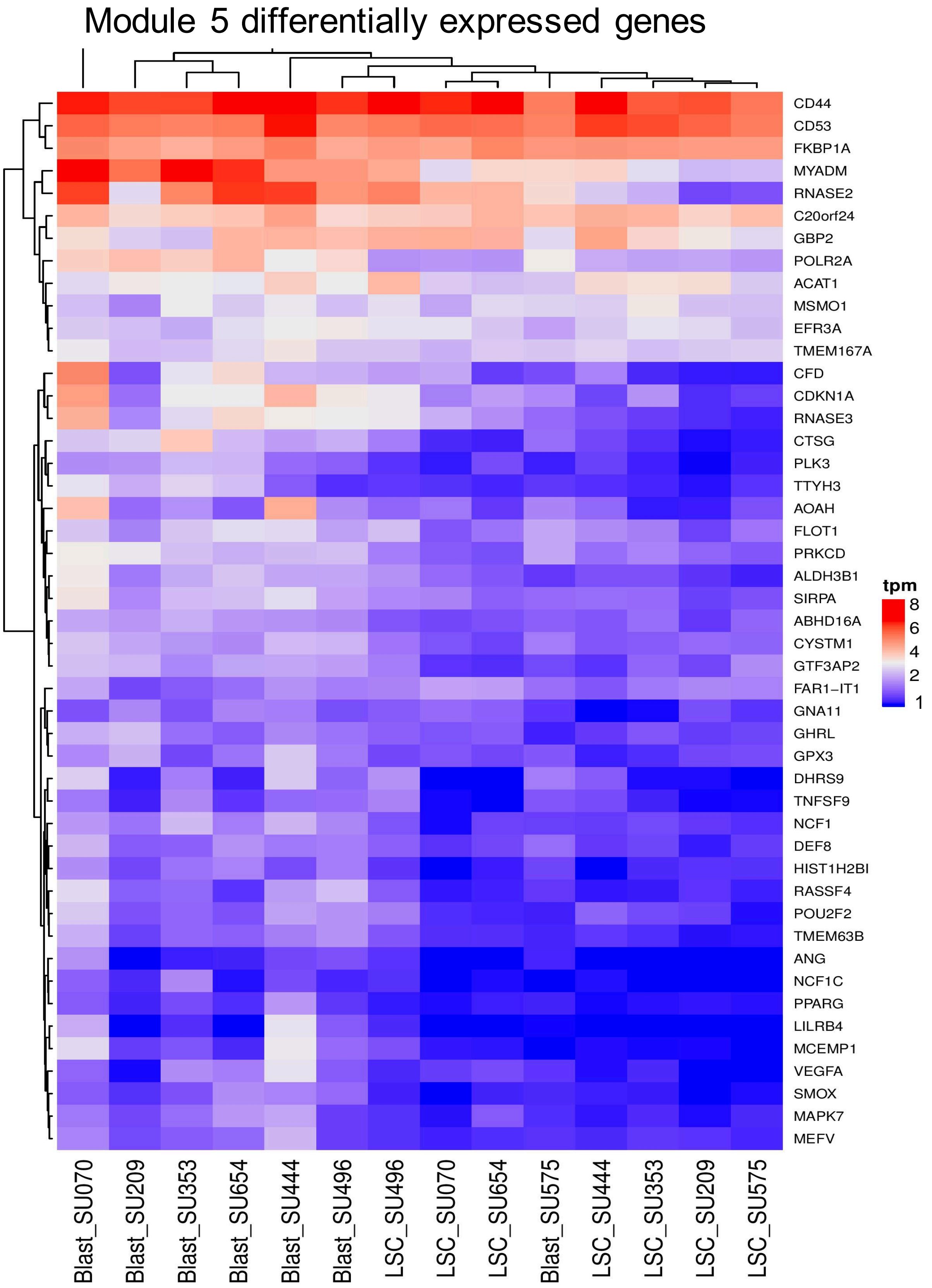
Module 5 differentially expressed genes
CD44
CD53
FKBP1A
MYADM
RNASE2
C20orf24
GBP2
POLR2A
ACAT1
MSMO1
EFR3A
TMEM167A
CFD
CDKN1A
RNASE3
CTSG
PLK3
TTYH3
AOAH
FLOT1
PRKCD
ALDH3B1
SIRPA
ABHD16A
CYSTM1
GTF3AP2
FAR1-IT1
GNA11
GHRL
GPX3
DHRS9
TNFSF9
NCF1
DEF8
HIST1H2BI
RASSF4
POU2F2
TMEM63B
ANG
NCF1C
PPARG
LILRB4
MCEMP1
VEGFA
SMOX
MAPK7
MEFV
tpm
8
6
4
2
1
Blast_SU070
Blast_SU209
Blast_SU353
Blast_SU654
Blast_SU444
Blast_SU496
LSC_SU496
LSC_SU070
LSC_SU654
Blast_SU575
LSC_SU444
LSC_SU353
LSC_SU209
LSC_SU575

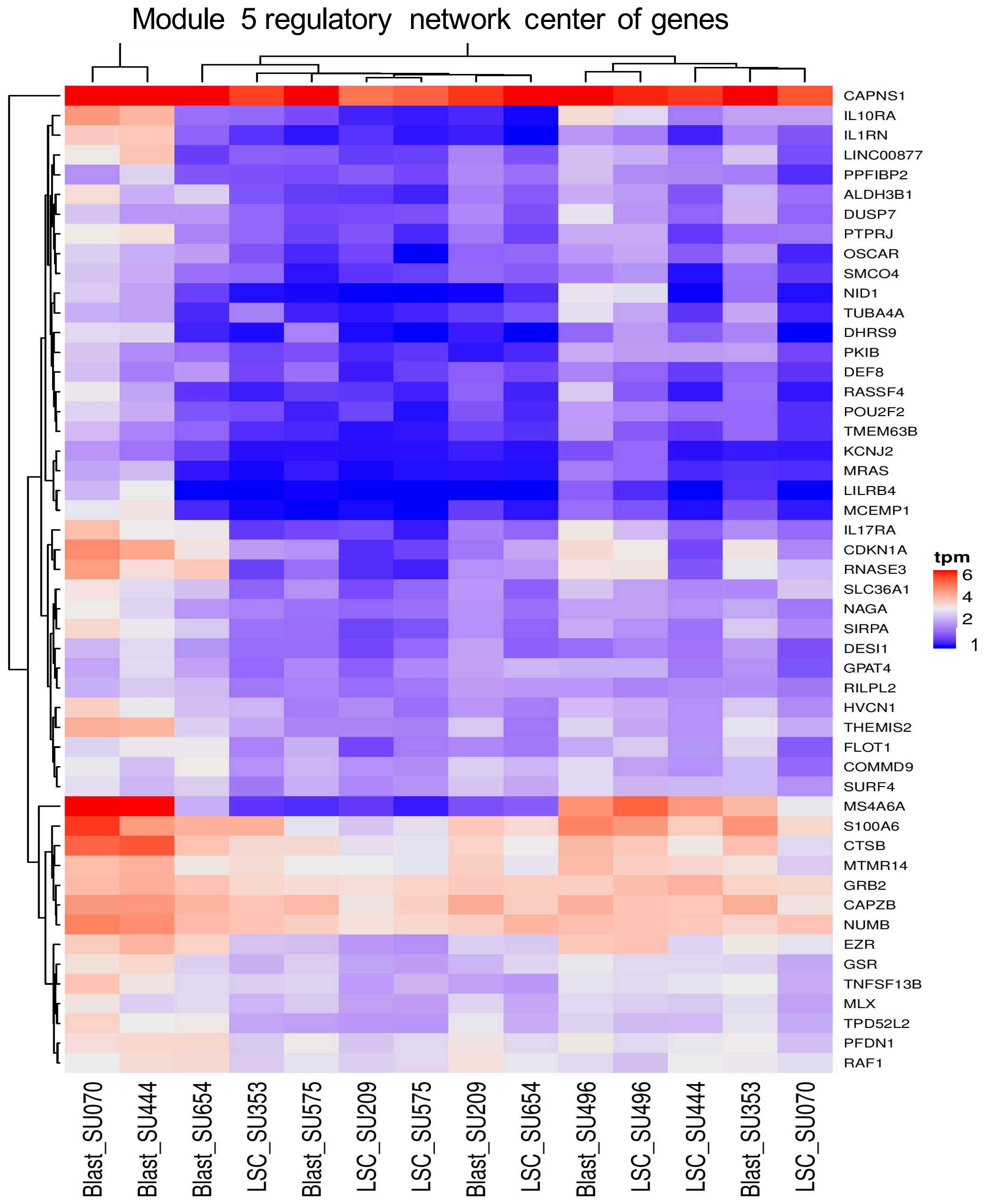

Depicts the Network-Hub Center Gene Expression for Module 5. The connected hub elements are most likely regulators within the module determined by a weighted gene network in a scale free topology.

Gene module 5 pathway enrichment comparing Blast to LSCs

pathways Activated in Module 5 Comparing Blast to LSC	logFC	pvalue	FDR
RESPONSE_TO_STRESS	0.73712062	0.003787561	0.025491116
CYTOKINE_BINDING	1.147375035	0.003787932	0.025491116
APOPTOSIS_GO	0.547532874	0.005741553	0.032072026
JAK_STAT_CASCADE	0.877177083	0.008940268	0.038793484
CYTOKINE_METABOLIC_PROCESS	0.8222507	0.010952116	0.041401797
DEFENSE_RESPONSE_TO_VIRUS	1.998632751	0.014142651	0.044639045
REGULATION_OF_JAK_STAT_CASCADE	0.597350493	0.026403027	0.067264855
CELLULAR_DEFENSE_RESPONSE	1.121487143	0.026977299	0.068402155
CYTOKINE_AND_CHEMOKINE_MEDIATED_SIG._PATHWAY	1.436830265	0.029336014	0.073169078
RESPONSE_TO_VIRUS	0.871575739	0.036905357	0.086788423
INTERFERON_GAMMA_PRODUCTION	0.802197911	0.043511936	0.093866474
INFLAMMATORY_RESPONSE	0.853829056	0.001087356	0.027183908
Inflammation (combined set of 649 genes)	0.664765453	0.00644078	0.066992012
GO_REGULATION_OF_INFLAMMATORY_RESPONSE	0.65492378	0.008039041	0.066992012
GO_INFLAMMATORY_RESPONSE	0.628374489	0.015782533	0.085430098
GO_POSITIVE_REGULATION_OF_ACUTE_INFLAMMATORY_RESPONSE	0.810982854	0.01708602	0.085430098
GO_POSITIVE_REGULATION_OF_INFLAMMATORY_RESPONSE	0.675001259	0.022006107	0.091692113
POSITIVE_REGULATION_OF_IMMUNE_SYSTEM_PROCESS	1.112970426	0.038742617	0.109801341
ADAPTIVE_IMMUNE_RESPONSE_GO_0002460	0.802197911	0.043511936	0.109801341
ADAPTIVE_IMMUNE_RESPONSE	0.802197911	0.043511936	0.109801341

In this comparison, positive fold change indicates that the genes were up-expressed in Blasts upon comparison with LSCs. This table of the enrichments relating to immunogeneic cellular defense of the gene network module 5. The 'pathway names' are the Molecular Signature Canonical GO pathway data base names. The 'logFC' is the log-fold change of the activation changes, where logFC>0 indicates higher activity in Blasts compared to LSCs, and vice versa. The 'pvalue' is the significance value, along the 'FDR' using Bonferroni Hochberg adjustment.

The pathway 'Immune' or 'Inflammation' were the combined MSigDB pathways, and the pathways in all capital letters are the raw MSigDB pathways (GO) related to immune defense or inflammatory signaling.

b

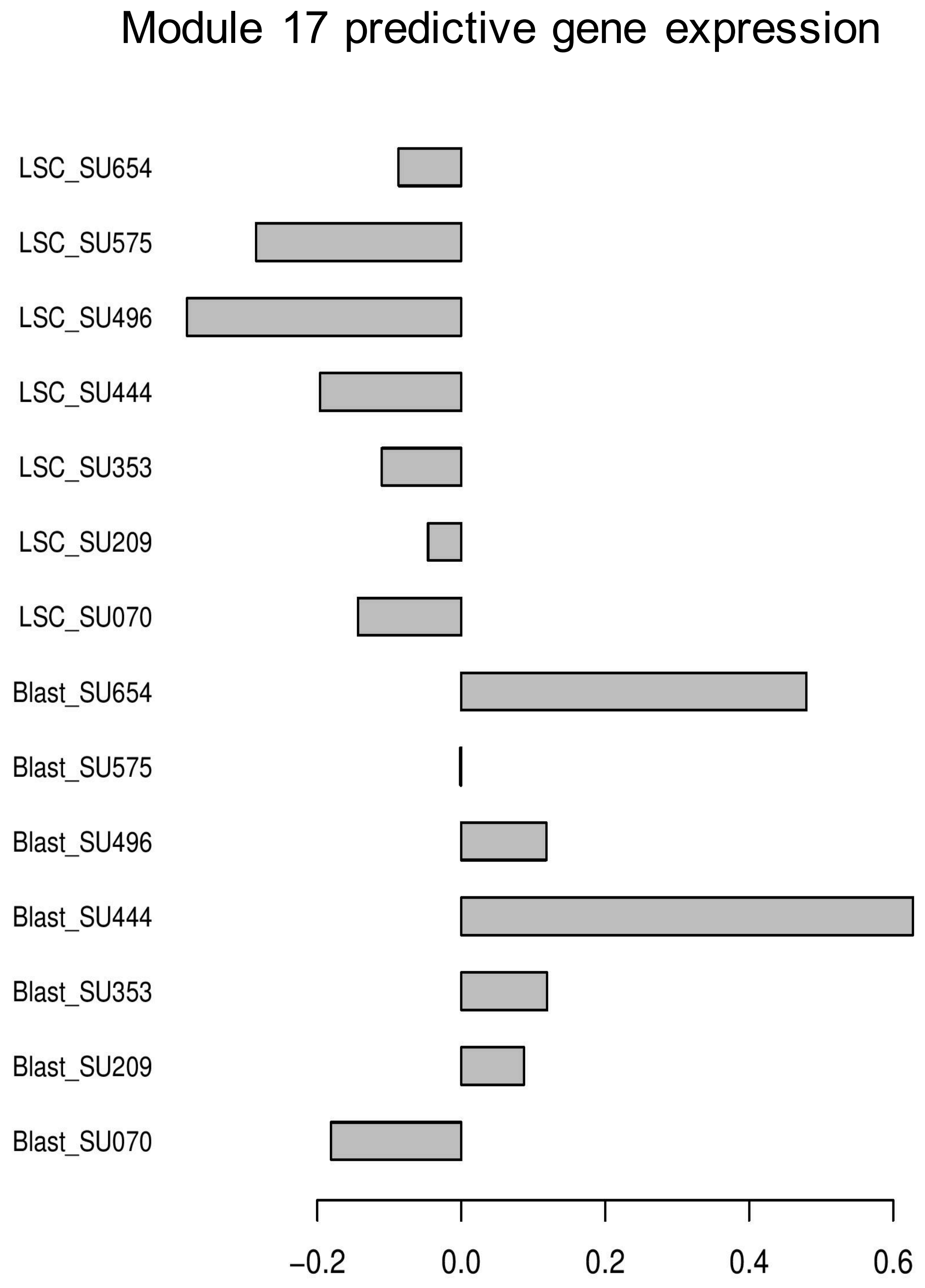

The labeling is similar to 'a'.

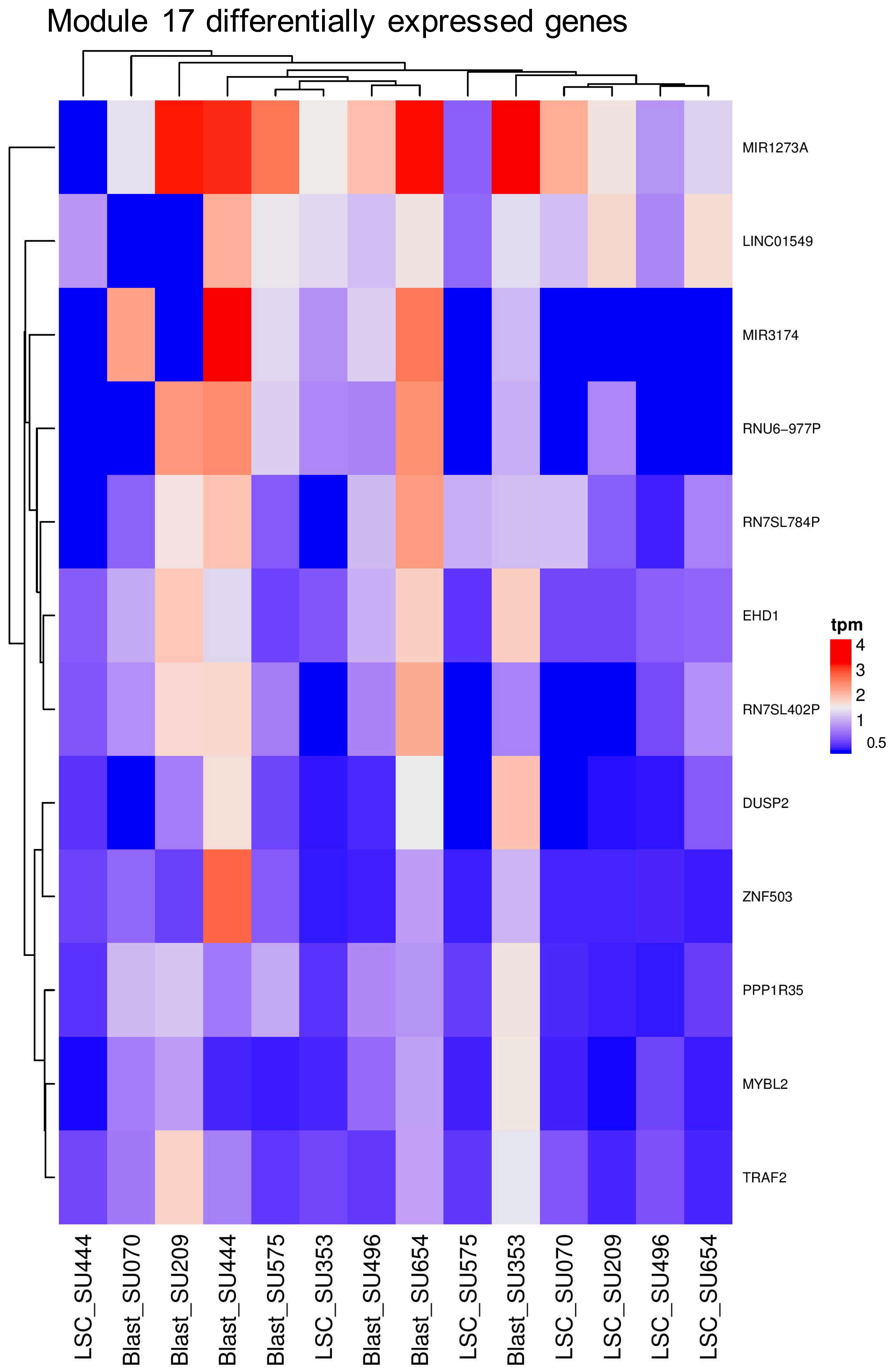
Module 17 differentially expressed genes
MIR1273A
LINC01549
MIR3174
RNU6−977P
RN7SL784P
EHD1
RN7SL402P
DUSP2
ZNF503
PPP1R35
MYBL2
TRAF2
tpm
4
3
2
1
0.5
LSC_SU444
Blast_SU070
Blast_SU209
Blast_SU444
Blast_SU575
LSC_SU353
Blast_SU496
Blast_SU654
LSC_SU575
Blast_SU353
LSC_SU070
LSC_SU209
LSC_SU496
LSC_SU654

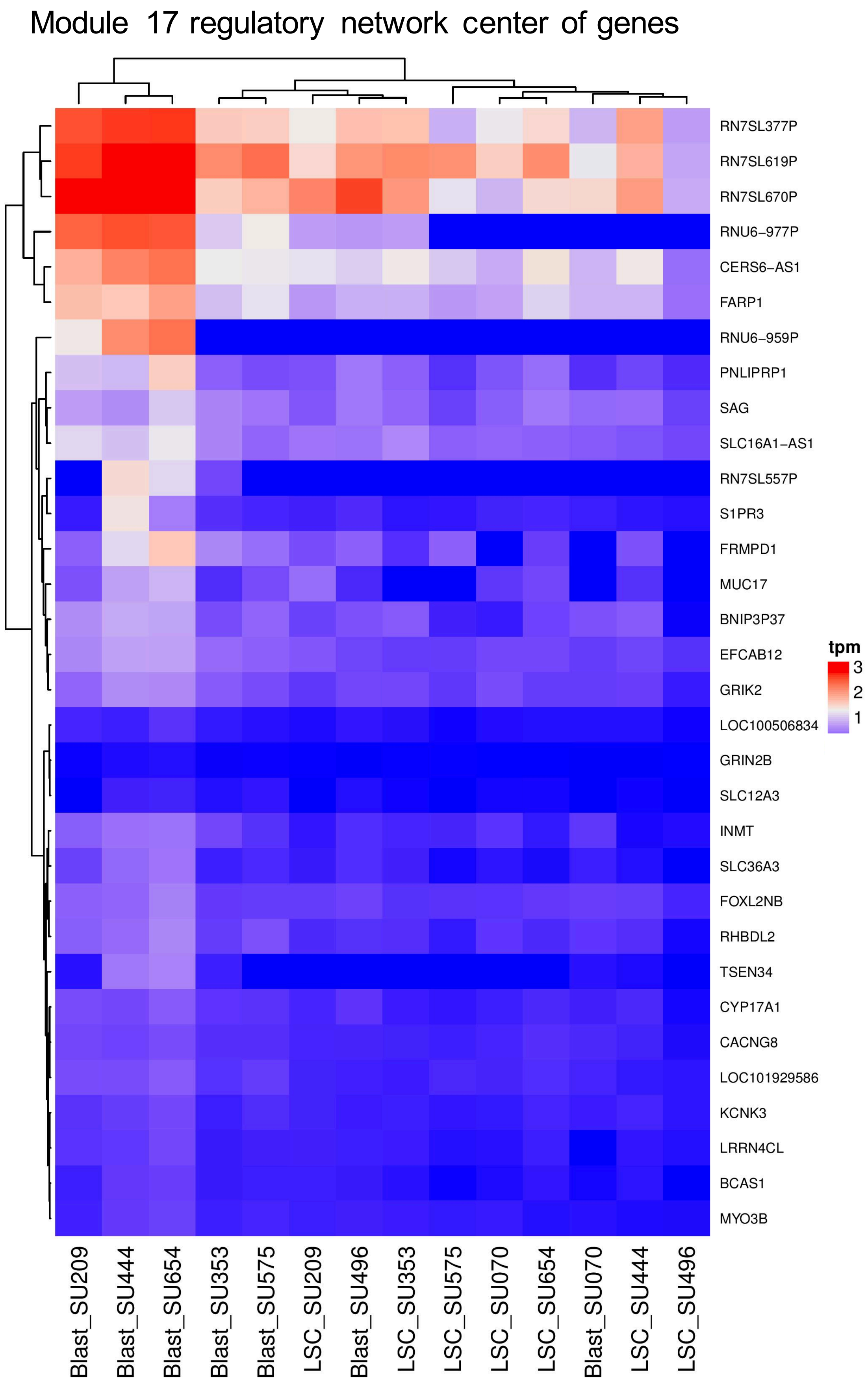

Labeling similar to a.

Gene module 17 pathway enrichment comparing Blast to LSCs

Pathways Activated in Module 17 Comparing Blast to LSC	logFC	pvalue	FDR
CYTOKINE_ACTIVITY	3.145798715	0.000380827	0.022673841
IMMUNE_SYSTEM_DEVELOPMENT	1.306245535	0.001353121	0.055121895
CYTOKINE_PRODUCTION	2.749896012	0.006140837	0.122064796
REGULATION_OF_APOPTOSIS	1.033138573	0.044238391	0.235916235
IMMUNE_SYSTEM_DEVELOPMENT	1.306245535	0.001353121	0.020437542
POSITIVE_REGULATION_OF_IMMUNE_SYSTEM_PROCESS	1.869404675	0.002786938	0.020437542
REGULATION_OF_IMMUNE_SYSTEM_PROCESS	1.869404675	0.002786938	0.020437542
POSITIVE_REGULATION_OF_IMMUNE_RESPONSE	2.749896012	0.006140837	0.024688135
REGULATION_OF_IMMUNE_RESPONSE	2.749896012	0.006140837	0.024688135
GO_INFLAMMATORY_RESPONSE	0.757497147	0.006733128	0.024688135
IMMUNE_SYSTEM_PROCESS	0.907993859	0.014782594	0.040652133
Immune (Combined set of 332 genes)	0.907993859	0.014782594	0.040652133

Labeling similar to a.

c

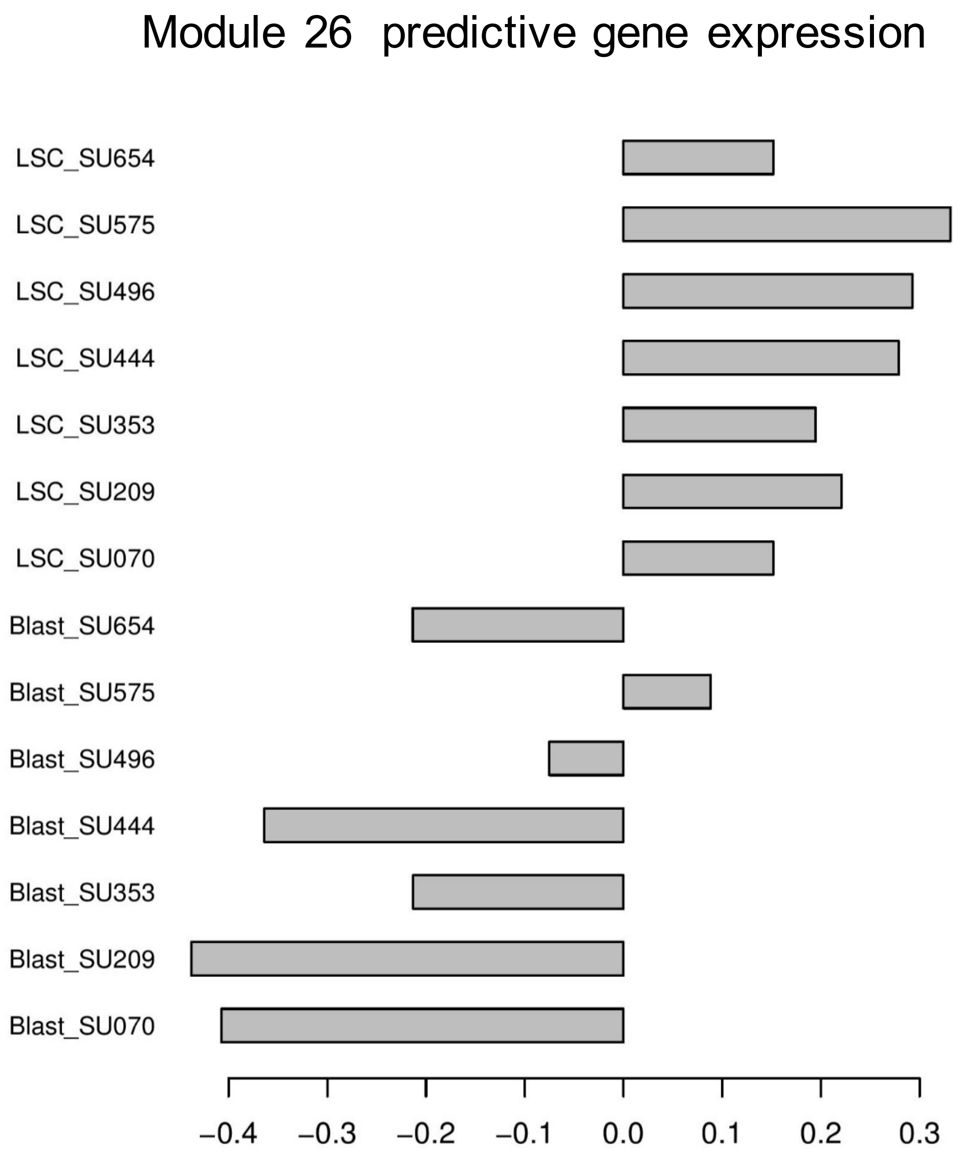

Labeling similar to a.

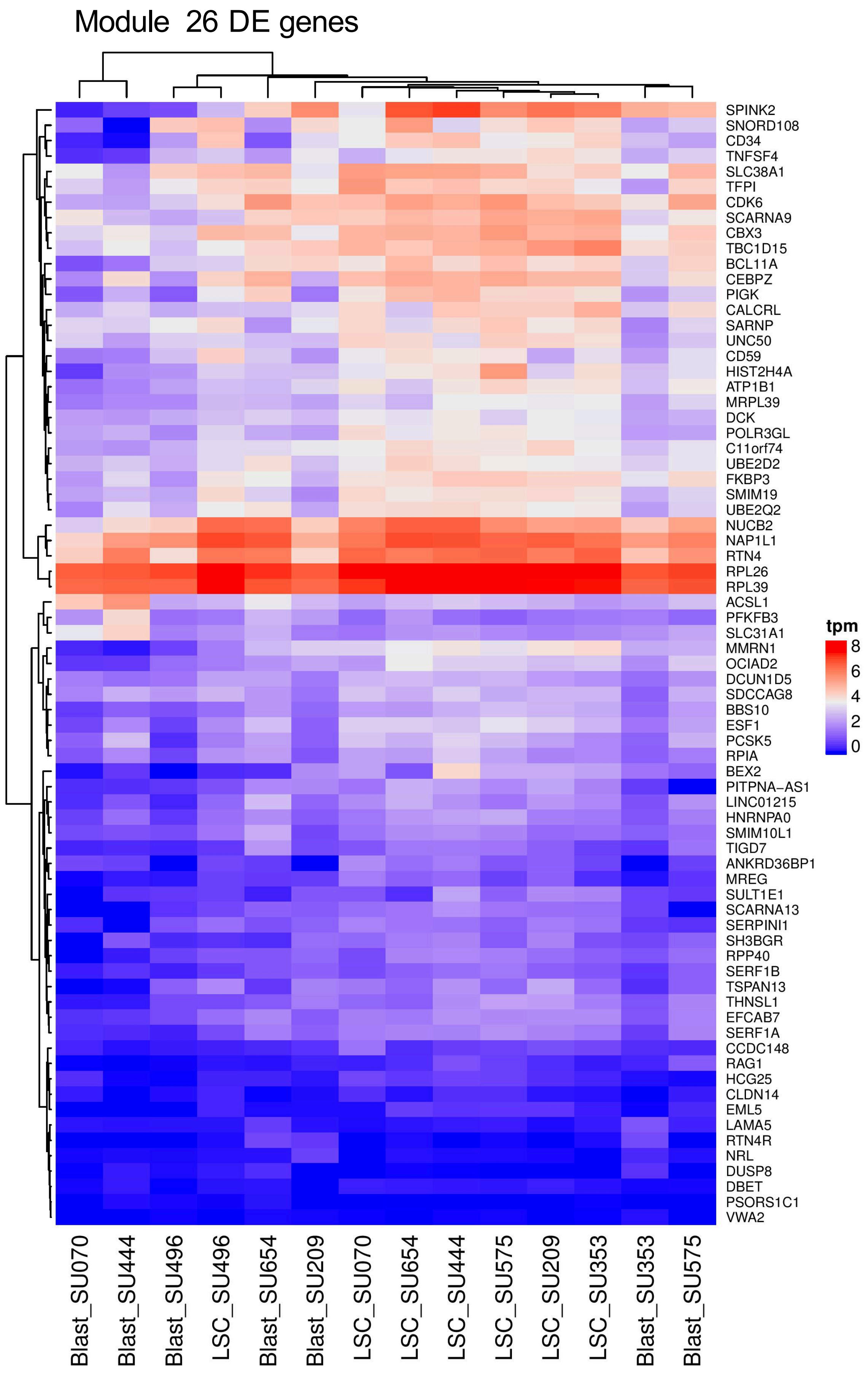

Labeling similar to a.

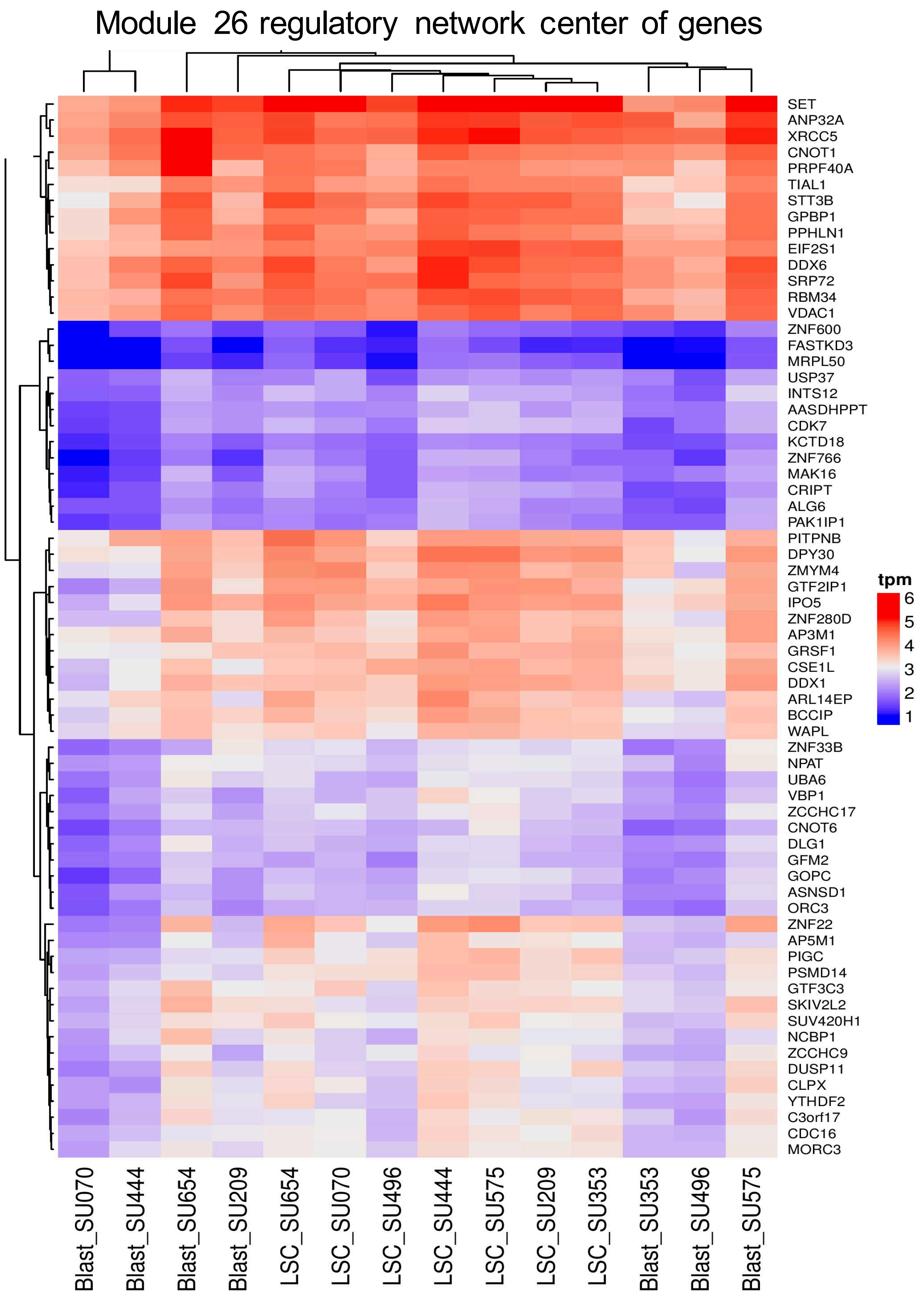

The labeling is similar to a.

The regulatory network center of genes for module 26 had signed connectivity scores (Horvath, et.al) greater than or equal to 0.9501.

Module 26 regulation gene members

ENSEMBL ID	Gene Name	Signed Connectivity Score
ENSG00000109606	DHX15	0.927732914
ENSG00000089876	DHX32	0.925782312
ENSG00000135829	DHX9	0.907240698
ENSG00000108406	DHX40	0.90666425
ENSG00000067248	DHX29	0.893110964
ENSG00000057663	ATG5	0.892508062
ENSG00000005893	LAMP2	0.869454175
ENSG00000174953	DHX36	0.817617173
ENSG00000277140	KIAA0430	0.790299888
ENSG00000080824	HSP90AA1	0.756040501
ENSG00000281763	DHX36	0.753513167
ENSG00000113360	DROSHA	0.74633069

Regulation genes in Figure 7 held high values for the signed eigen-based connectivity score (Horvath et.al) which indicates that the regulating genes in Figure 7 held membership within the same module (26). This is sensible because the network is created using collections of genes that are co-expressed, or well-correlated. Figure 7 depicts regulating genes with similar expression profiles, and this is supported by network membership assignment which collected these genes into the same eigen-gene-module.

Note many of these are missing from the previous central-hub network heatmap because the center hub genes had higher more competitive signed connectivity scores.

Gene module 26 pathway enrichment comparing Blast to LSCs

pathways Activated in Module 26 Comparing Blast to LSC	logFC	pvalue	FDR
CYTOKINE_ACTIVITY	0.365511385	0.00040149	0.028626156
NF_KAPPAB_BINDING	-0.39814468	0.000544961	0.030784931
DNA_INTEGRITY_CHECKPOINT	-0.217330358	0.000656442	0.030784931
DNA_DAMAGE_CHECKPOINT	-0.211183297	0.001784519	0.047362917
RESPONSE_TO_WOUNDING	-0.363775049	0.002244836	0.047362917
WOUND_HEALING	-0.956605049	0.002892206	0.047362917
BLOOD_COAGULATION	-1.172743357	0.002933145	0.047362917
DOUBLE_STRANDED_RNA_BINDING	0.160658226	0.003569842	0.048355137
IMMUNE_SYSTEM_PROCESS	-0.17732978	0.004040075	0.051176777
IMMUNE_SYSTEM_DEVELOPMENT	-0.229180206	0.006964717	0.077187487
GO_REGULATION_OF_INFLAMMATORY_RESPONSE	-0.232805492	0.003011986	0.032320601
IMMUNE_SYSTEM_PROCESS	-0.17732978	0.004040075	0.032320601
Immune (combined set of 332 genes)	-0.17732978	0.004040075	0.032320601
IMMUNE_SYSTEM_DEVELOPMENT	-0.229180206	0.006964717	0.041788304
Inflammation (combined set of 649 genes)	-0.111531907	0.013995151	0.067176723

Labeling similar to A.

d

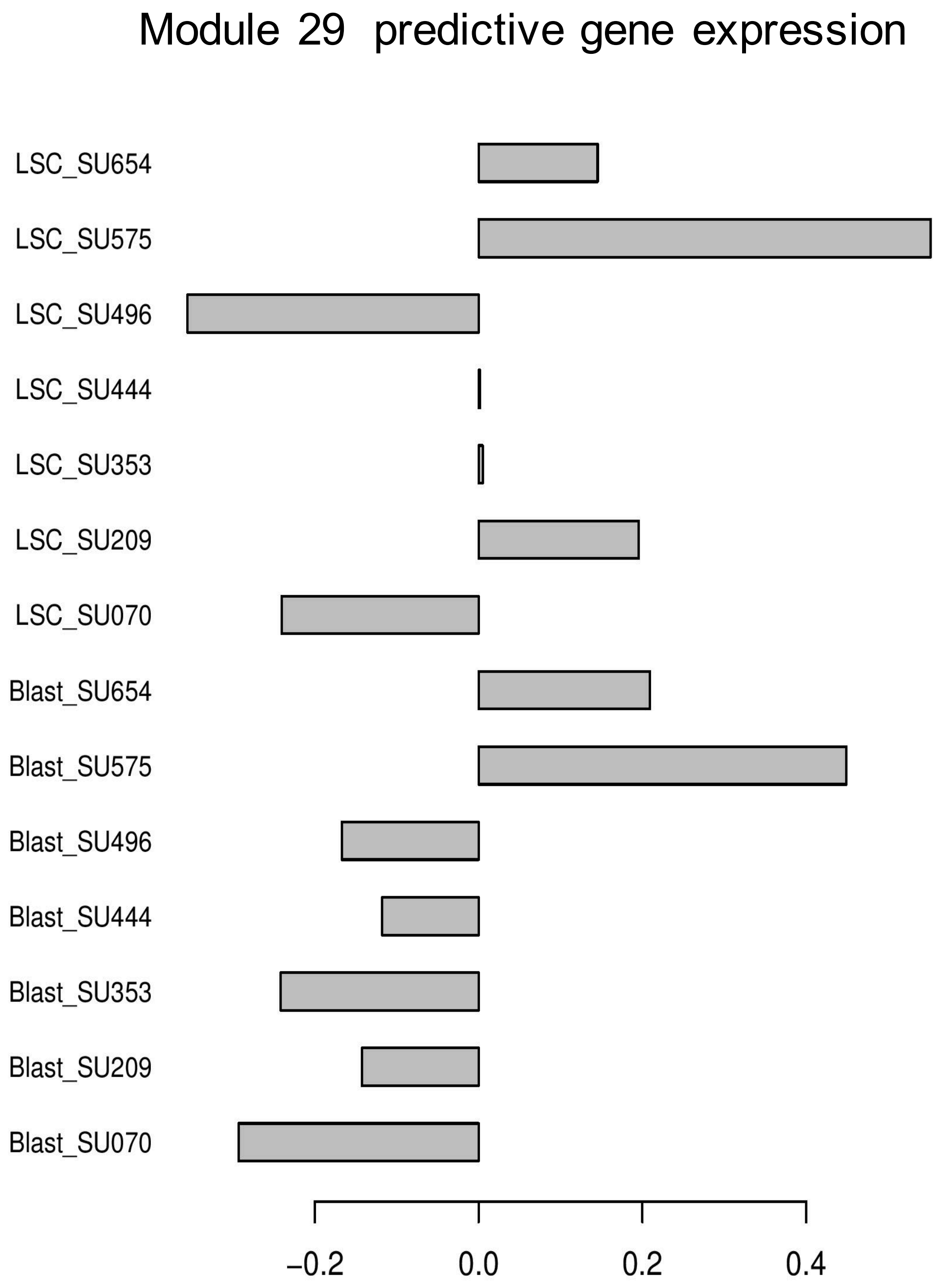

Labeling similar to “a”.

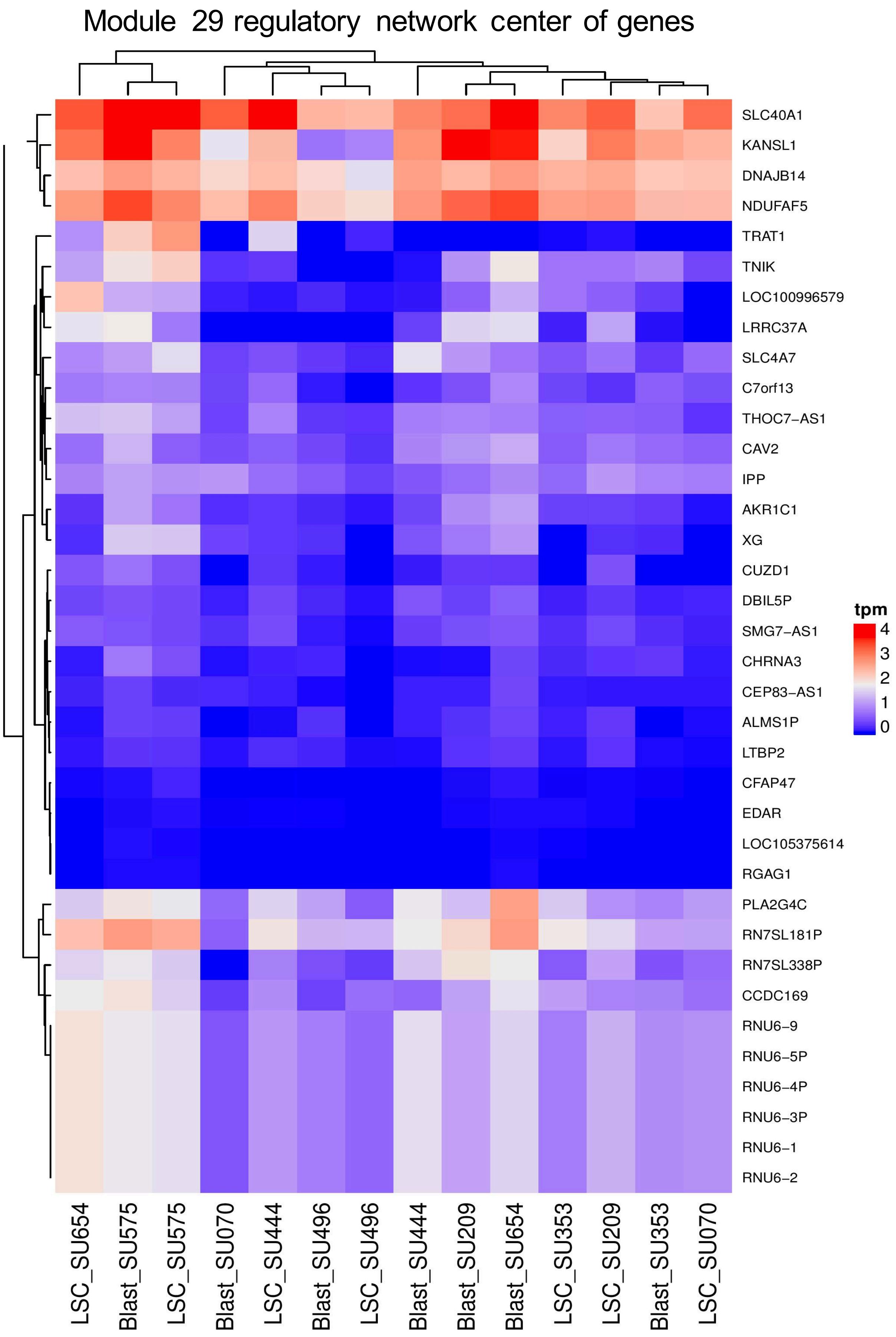

Labeling similar to a.

Gene module 29 pathway enrichment comparing Blast to LSCs

Pathways Activated in Module 29 Comparing Blast to LSC	logFC	pvalue	FDR
IMMUNOLOGICAL_SYNAPSE	-7.998884155	0.00702873	0.070547624
Immune (combined gene set of 332 genes)	-7.998884155	0.00702873	0.00859067

Labeling similar to a.

e

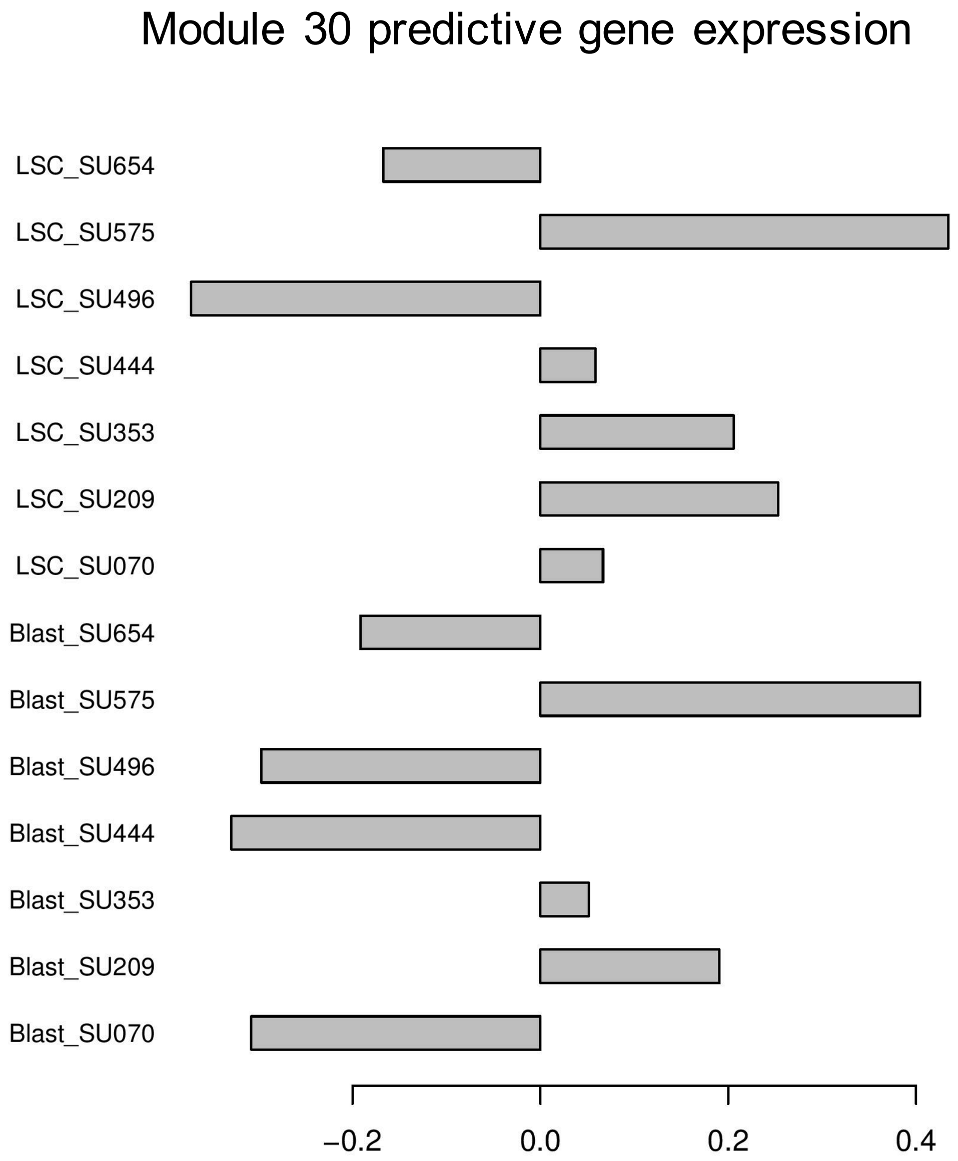

Labeling similar to a.

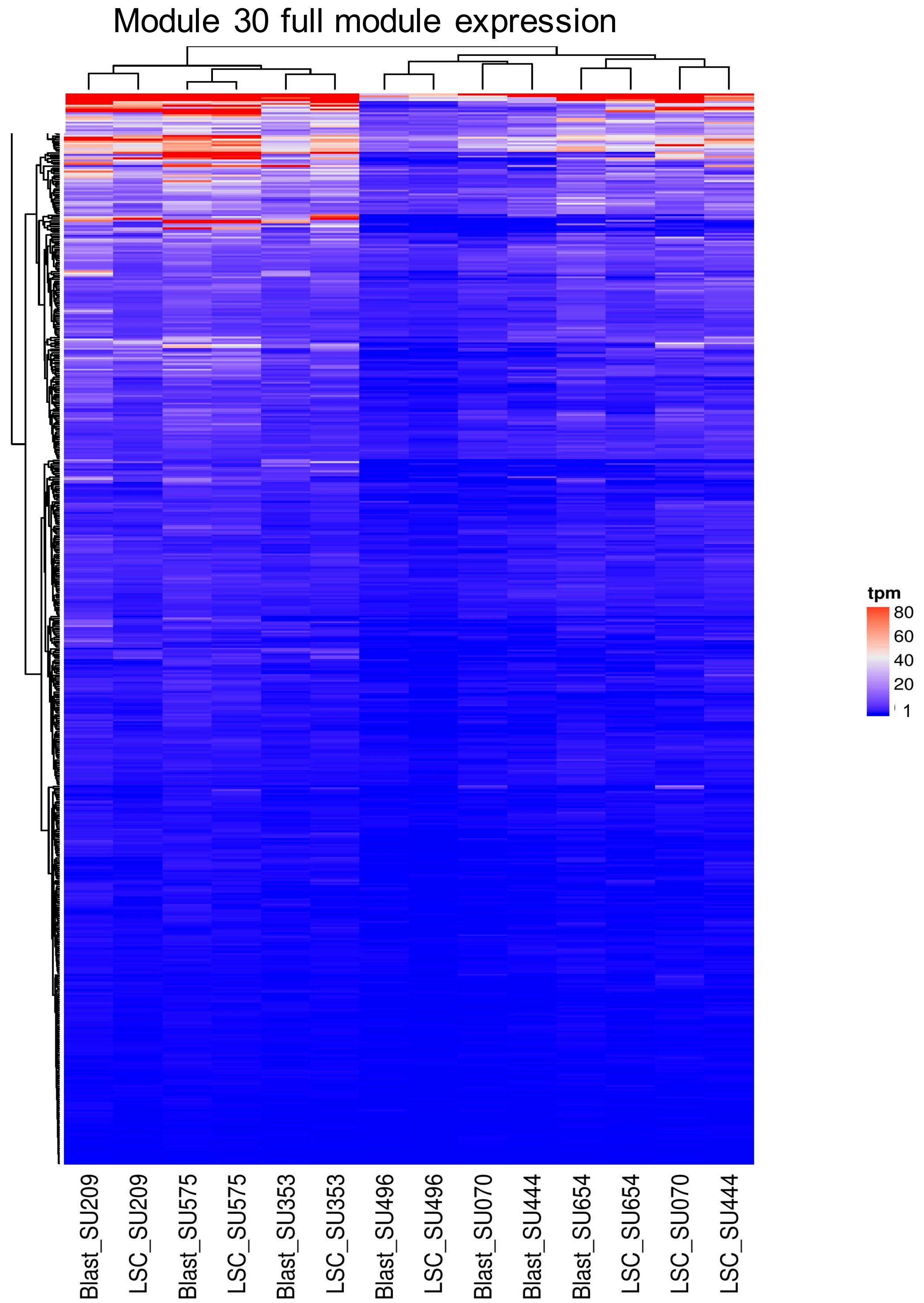

Labeling similar to a.

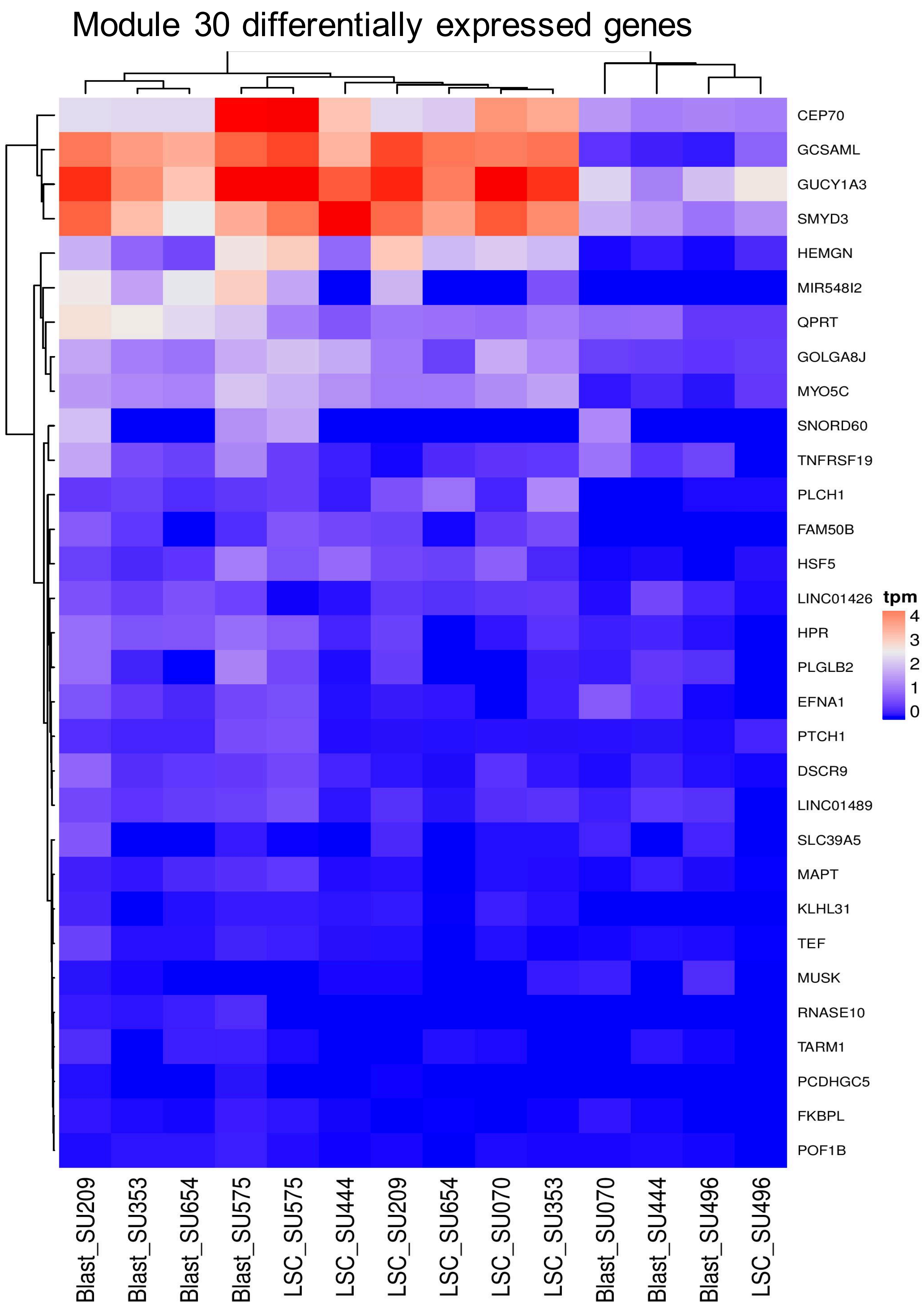

Labeling similar to a.

Module 30 regulatory network center of genes

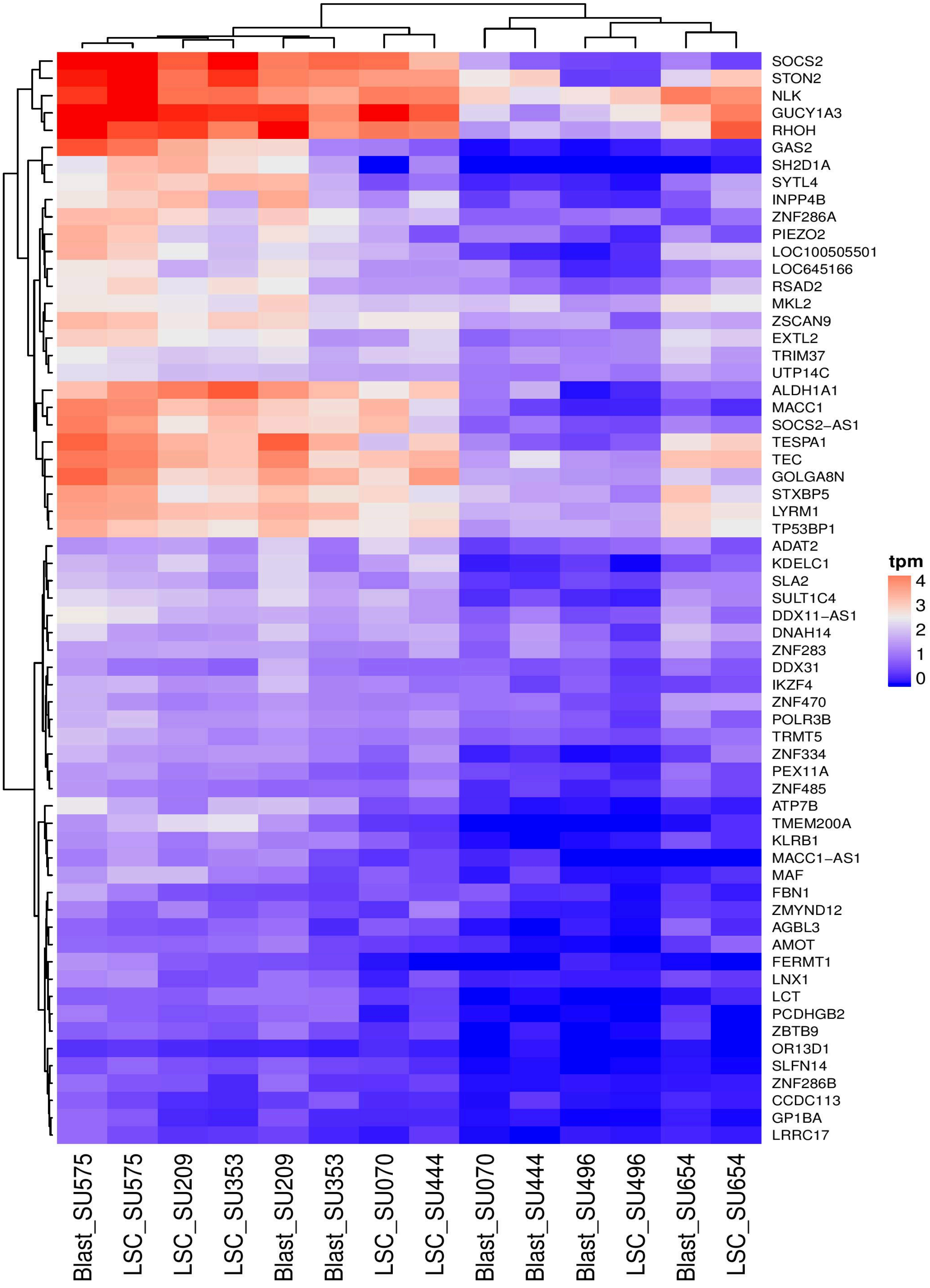

Labeling similar to a.

Gene module 30 pathway enrichment comparing Blast to LSCs

Pathways Activated in Module 30 Comparing Blast to LSC	logFC	pvalue	FDR
RESPONSE_TO_VIRUS	-0.896821468	0.003219836	0.111084341
DEFENSE_RESPONSE_TO_VIRUS	-0.896821468	0.003219836	0.111084341
JAK_STAT_CASCADE	-0.806848519	0.007519285	0.163841261
IMMUNE_EFFECTOR_PROCESS	-0.754468827	0.028439388	0.375582856

Labeling similar to a.

Supplement figure 6

a DE Immune genes comparing high risk to low risk MDS

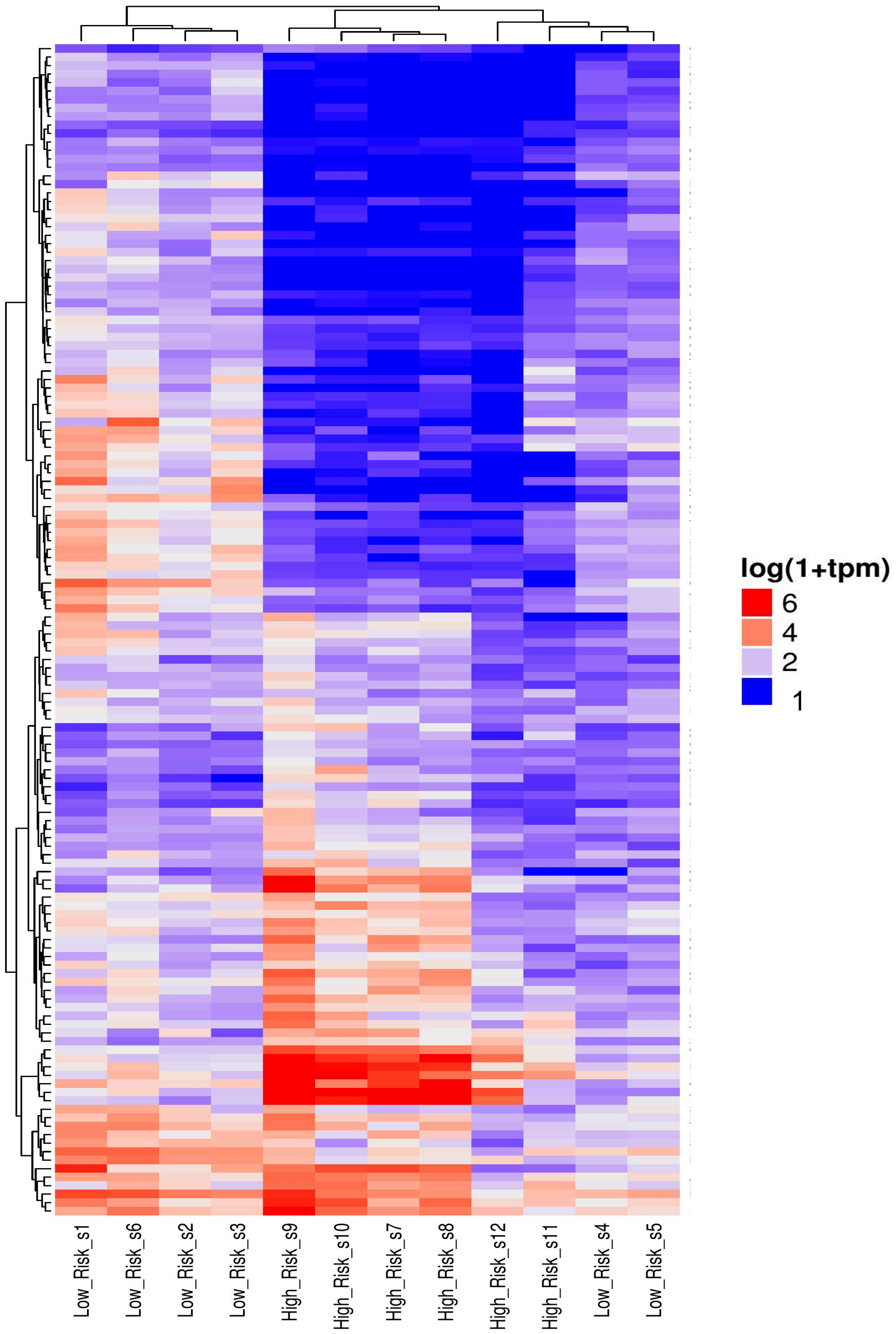

Differentially expressed genes using the merged MSigDB (GO) gene set for the 17 canonical immune related pathways comparing High-Low Risk MDS. This corresponds to Figure 5B. The immune gene set contained 335 unique genes.

b DE Inflammation genes comparing high risk to low risk MDS

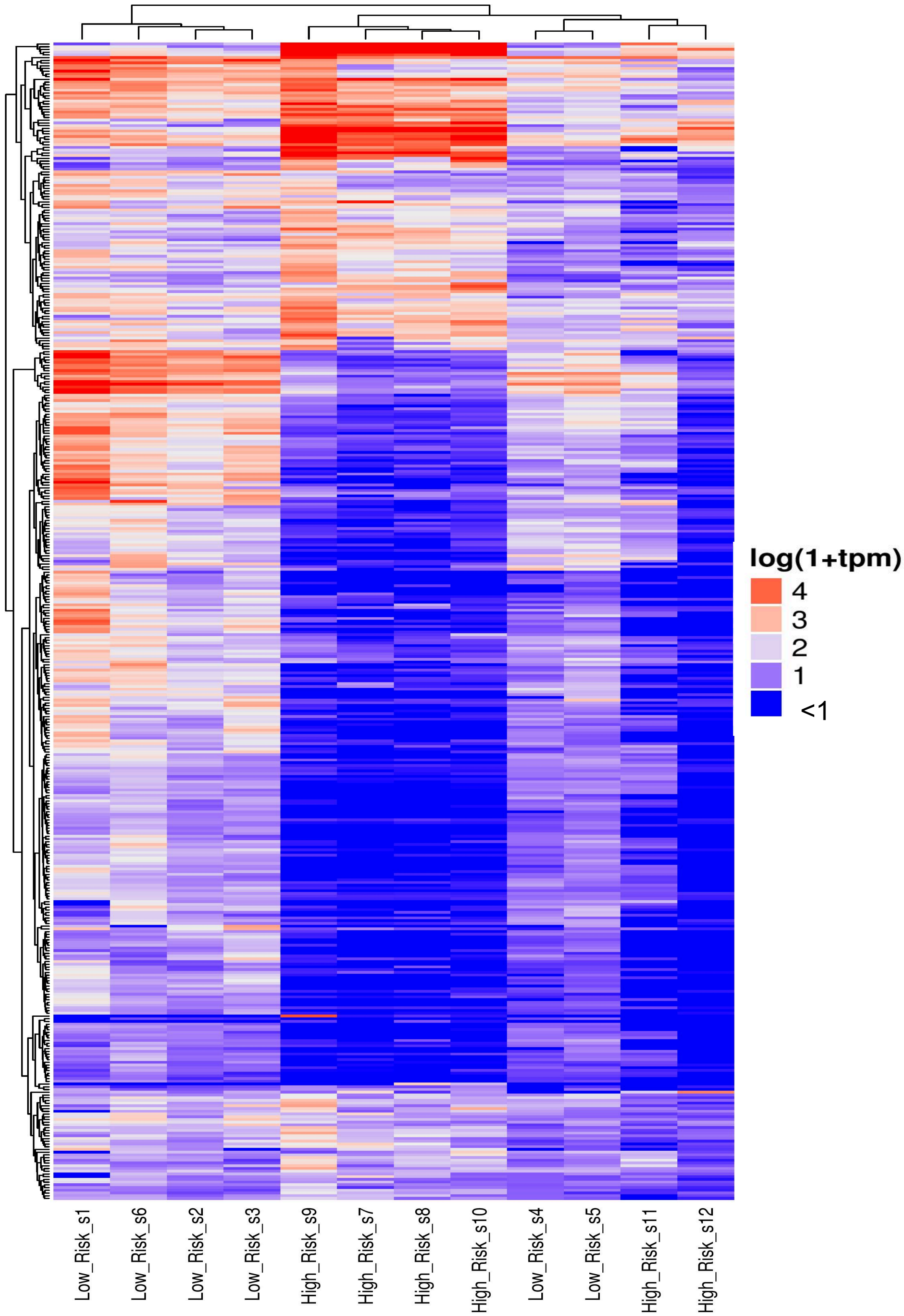

Differentially expressed genes using the merged MSigDB (GO) gene set for the 7 canonical inflammation related pathways comparing High-Low Risk MDS. This corresponds to Figure 5B. The inflammation gene set contained 649 unique genes.

c

High risk – low risk MDS (GO) MSigDB *p.value≤0.05

High Risk vs. Low Risk MDS	log.FC	p.Value	FDR
Regulation_of_immune_response*	-0.826661522	1.50E-06	1.87E-05
Innate_immune_response*	-1.248823055	2.48E-06	1.87E-05
Go_positive_regulation_of_inflammatory_response*	-0.788172612	3.15E-06	1.87E-05
Positive_regulation_of_immune_response*	-0.679649139	3.19E-06	1.87E-05
Regulation_of_immune_system_process*	-0.730952069	3.59E-06	1.87E-05
Go_positive_regulation_of_acute_inflammatory_response*	-1.013981687	4.88E-06	1.98E-05
Go_regulation_of_inflammatory_response*	-0.664575284	5.34E-06	1.98E-05
Acute_inflammatory_response*	-1.300236834	1.04E-05	3.39E-05
Positive_regulation_of_immune_system_process*	-0.607817509	3.07E-05	8.86E-05
Humoral_immune_response*	-0.744725226	5.43E-05	0.000141152
Go_acute_inflammatory_response*	-0.63590795	0.000122822	0.000277876
Activation_of_immune_response*	-0.459257594	0.00012825	0.000277876
Go_inflammatory_response*	-0.608314674	0.000144931	0.000289863
Inflammation (combined Set of 649 Genes)*	-0.609470501	0.00018147	0.000337016
Immune_effector_process*	-0.349297635	0.000316873	0.000549247
Immunological_synapse*	-0.681017181	0.000455943	0.000740907
Inflammatory_response*	-0.497136557	0.002448142	0.003744217
Immune_response*	-0.349610522	0.01394012	0.020135729
Negative_regulation_of_immune_system_process*	-0.578305957	0.018673639	0.025553401
Regulation_of_immune_effector_process*	-0.434351448	0.021906899	0.028478968

All pathways used in the depiction of the immune/inflammation set analysis in Figure 5B. The merged set of 335 genes for 'Immune' was not significant and thus filtered.

Supplement figure 7

a

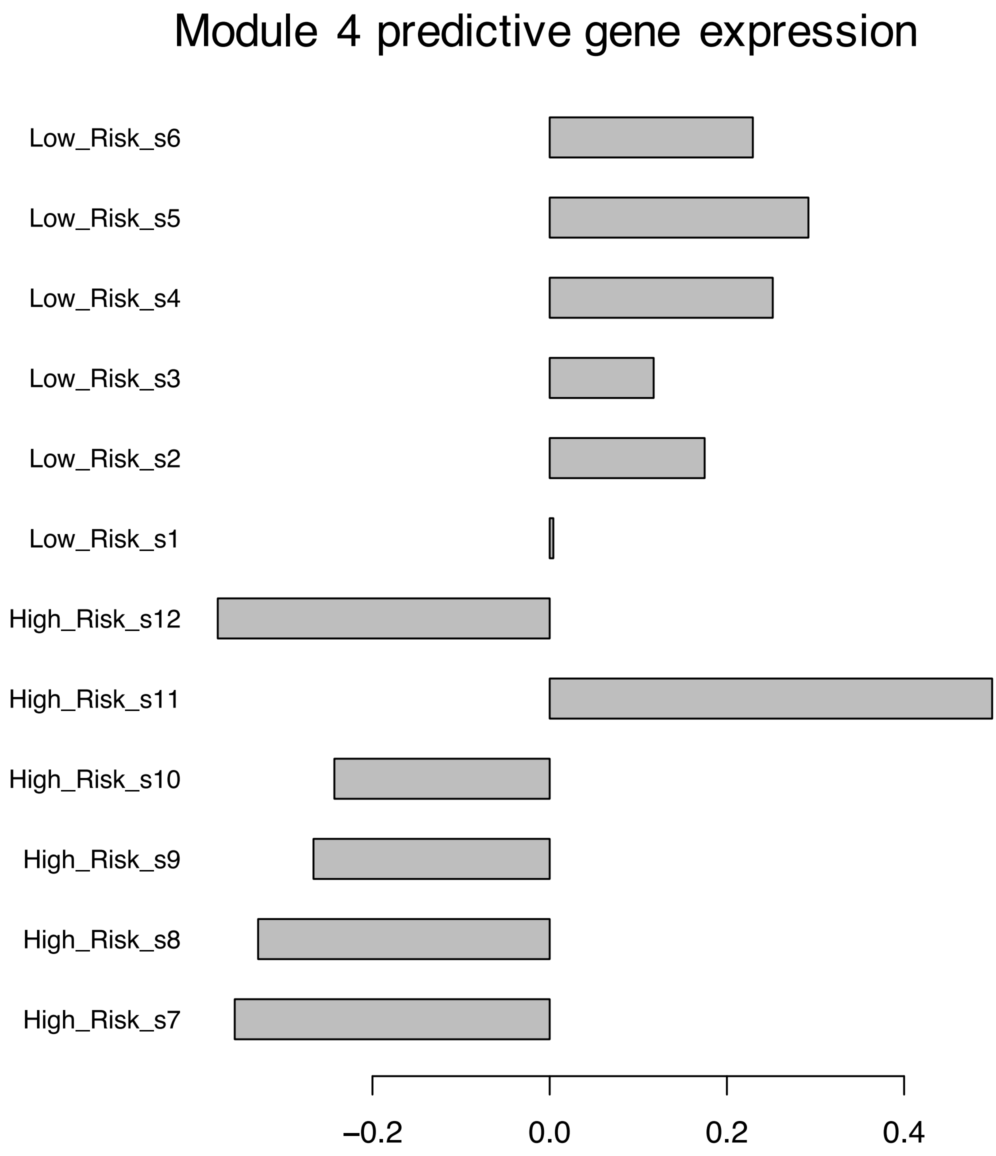

Depicts 'Eigen-Gene' (Module) Number 4 Expression Levels For Each Sample Group, The Module Number is located in the title, which corresponds to the 4th row in the main Figure 5.C. The y-axis denotes the sample. The x-axis denotes the predictive expression levels, where the positive/negative x-axis values denotes as to whether a sample is likely to be high/low expressed in that given module respectively.

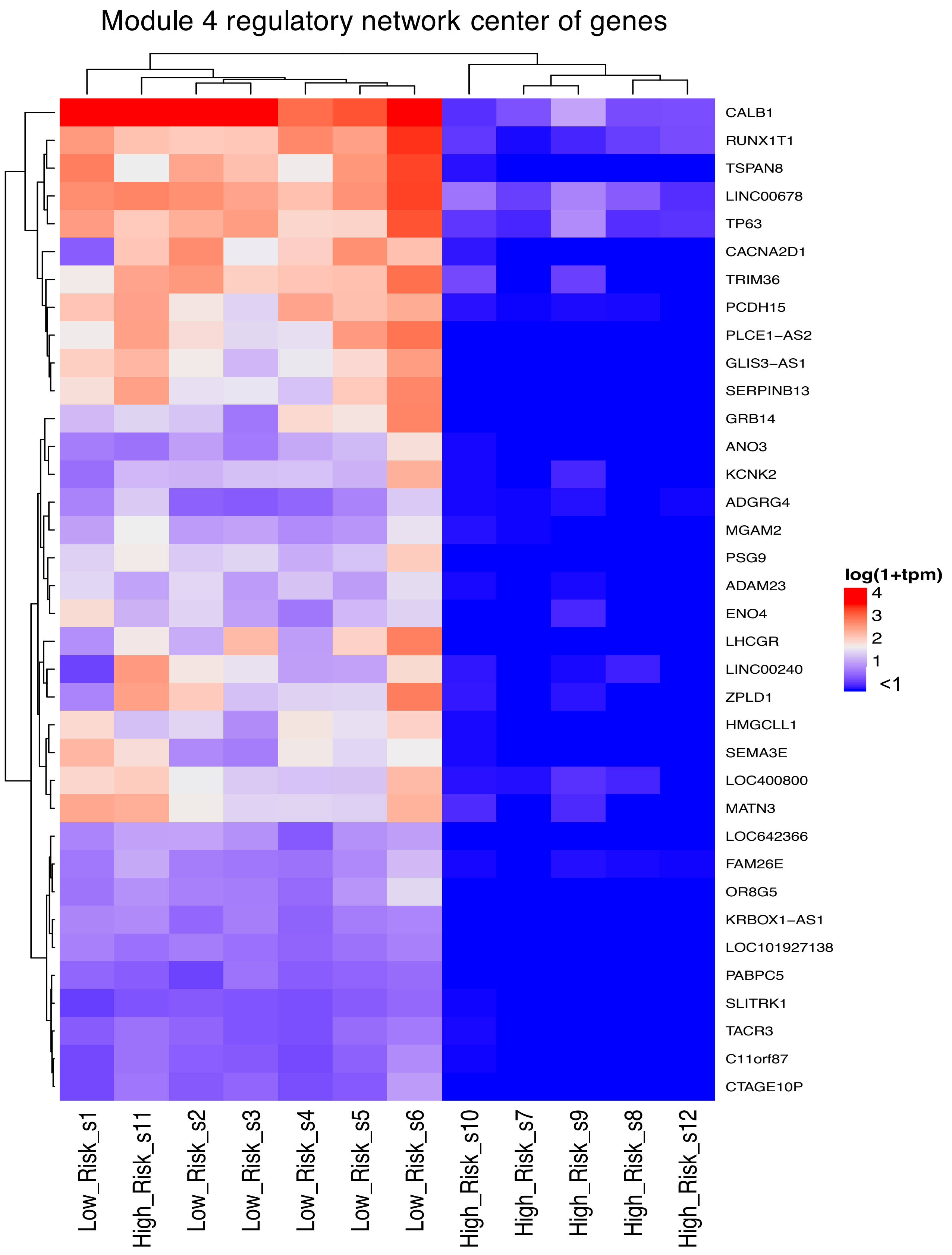

The second depicts the Network-Hub Center Gene Expression respectively. The x-axis denotes each sample. The y-axis denotes the expression of each gene in hub of module 4, where the expression values are in TPM.

Pathway (GO) analysis for module 4 gene network

pathway_name	logFC	pvalue	FDR	contrast	Module Num.
PHAGOCYTOSIS	5.389855348	0.000286861	0.009776094	Low-high	4
HUMORAL_IMMUNE_RESPONSE	3.931008189	0.000524732	0.009776094	Low-high	4
INFLAMMATORY_RESPONSE	3.035627101	0.000702343	0.009776094	Low-high	4
CYTOKINE_ACTIVITY	2.889120267	0.00100394	0.009776094	Low-high	4
APOPTOSIS_GO	1.923844393	0.001005971	0.009776094	Low-high	4
INDUCTION_OF_APOPTOSIS_BY_EXTRACELLULAR_SIGNALS	4.985151094	0.00107445	0.009776094	Low-high	4
RESPONSE_TO_BACTERIUM	3.699946766	0.001308469	0.009776094	Low-high	4
REGULATION_OF_PROGRAMMED_CELL_DEATH	1.910507601	0.001544446	0.010354431	Low-high	4
RESPONSE_TO_WOUNDING	2.59764459	0.001592396	0.010441501	Low-high	4
VIRAL_INFECTIOUS_CYCLE	4.15790174	0.001733976	0.010550159	Low-high	4
INNATE_IMMUNE_RESPONSE	3.817591272	0.002128497	0.011212387	Low-high	4
CHEMOKINE_RECEPTOR_BINDING	2.898455546	0.002154586	0.011212387	Low-high	4
RESPONSE_TO_RADIATION	3.352032368	0.002462188	0.01169237	Low-high	4
RESPONSE_TO_STRESS	1.898782097	0.002473878	0.01169237	Low-high	4
HEMATOPOIETIN_INTERFERON_CLASSD200_DOMAIN_CYTOKINE_RECEPTOR_BINDING	3.717784554	0.002667444	0.012490483	Low-high	4
ANTI_APOPTOSIS	1.89631606	0.002718528	0.012490483	Low-high	4
JAK_STAT_CASCADE	3.555634807	0.002909604	0.012874481	Low-high	4
DEFENSE_RESPONSE	2.254295802	0.003055563	0.013322683	Low-high	4
ACUTE_INFLAMMATORY_RESPONSE	3.954706675	0.003203311	0.013495031	Low-high	4
RESPONSE_TO_UV	2.58555828	0.00397701	0.015004683	Low-high	4
IMMUNE_EFFECTOR_PROCESS	1.725930204	0.004084647	0.015074942	Low-high	4
IMMUNE_RESPONSE	2.127470782	0.006202402	0.018678856	Low-high	4
T_CELL_ACTIVATION	2.725009204	0.006234944	0.018687537	Low-high	4
IMMUNE_SYSTEM_PROCESS	2.01654836	0.007380474	0.020007504	Low-high	4
CELLULAR_RESPONSE_TO_STRESS	4.542675349	0.007913616	0.021086066	Low-high	4
REGULATION_OF_IMMUNE_SYSTEM_PROCESS	2.277713416	0.008120606	0.021363704	Low-high	4
CYTOKINE_BINDING	2.686208055	0.011381039	0.026556455	Low-high	4
REGULATION_OF_IMMUNE_RESPONSE	3.285162322	0.012871031	0.028849939	Low-high	4
APOPTOTIC_PROGRAM	2.14706546	0.012955867	0.028849939	Low-high	4
RESPONSE_TO_VIRUS	3.310530543	0.013448174	0.02978663	Low-high	4
COAGULATION	1.277414856	0.01979725	0.039626278	Low-high	4
WOUND_HEALING	1.555922672	0.025678043	0.047437807	Low-high	4
CYTOKINE_PRODUCTION	1.138161601	0.046635764	0.074941749	Low-high	4
POSITIVE_REGULATION_OF_IMMUNE_RESPONSE	2.67655398	0.048307046	0.077328481	Low-high	4
CELLULAR_DEFENSE_RESPONSE	1.611837945	0.049474204	0.078893008	Low-high	4
ANTIGEN_BINDING	2.146705777	0.053793879	0.084912616	Low-high	4
CELL_STRUCTURE_DISASSEMBLY_DURING_APOPTOSIS	1.719721403	0.080155289	0.118148517	Low-high	4
CYTOCHROME_C_OXIDASE_ACTIVITY	-0.642253506	0.083468834	0.12202302	Low-high	4
REGULATION_OF_I_KAPPAB_KINASE_NF_KAPPAB_CASCADE	1.258710963	0.085184567	0.123349407	Low-high	4
NEURON_APOPTOSIS	2.786502401	0.086711046	0.124011241	Low-high	4
I_KAPPAB_KINASE_NF_KAPPAB_CASCADE	1.204787656	0.087207446	0.124141193	Low-high	4
APOPTOTIC_NUCLEAR_CHANGES	1.5272371	0.090479238	0.127488825	Low-high	4

Includes a table of the enrichments relating to immunogeneic cellular defense of the gene network module 4 that were significantly enriched with comparing to Low Risk MDS to High Risk MDS. The 'pathway names' are the Molecular Signature Canonical GO pathway data base names. The ‘logFC' is the log-fold change of the activation changes with the direction of change with respect to the 'contrast'. The 'pvalue' is the significance value, along the 'FDR' using Bonferroni Hochberg filtering. Note that positive log fold change indicates that the Low-Risk MDS were higher expressed/activated for a given gene set compared to High Risk MDS, and vice versa.

b

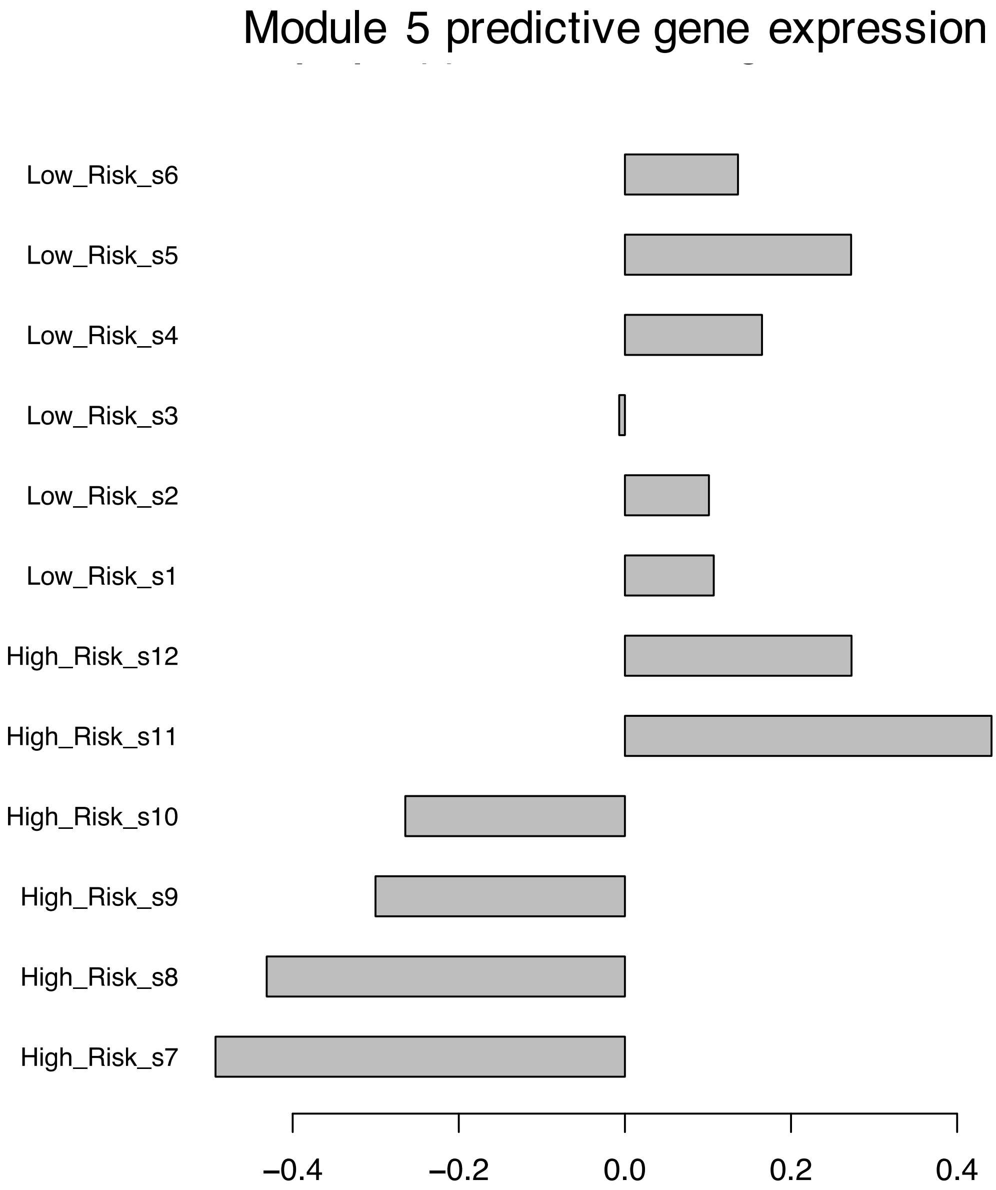

Labeling is similar to A.

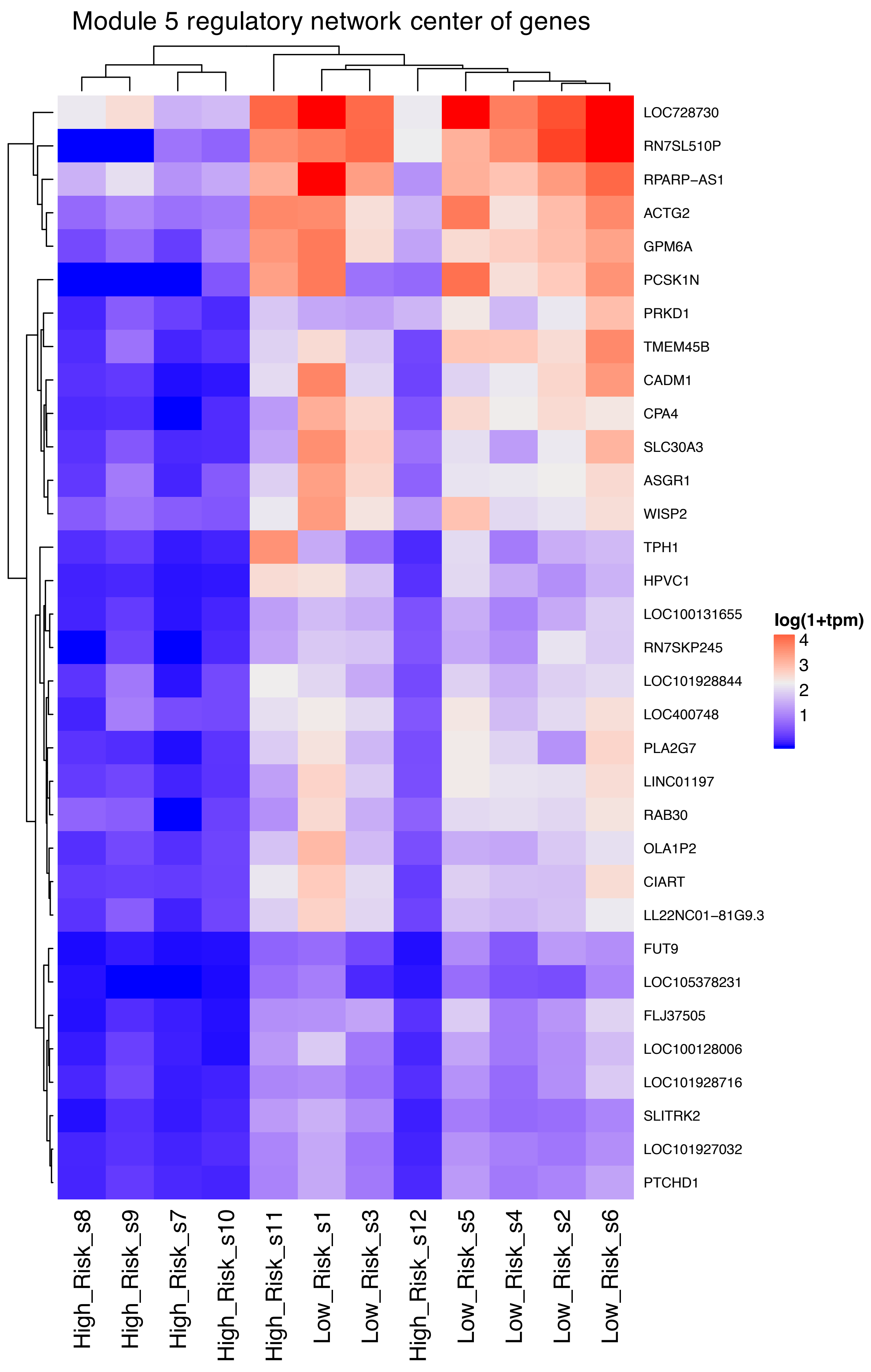
Module 5 regulatory network center of genes
LOC728730
RN7SL510P
RPARP-AS1
ACTG2
GPM6A
PCSK1N
PRKD1
TMEM45B
CADM1
CPA4
SLC30A3
ASGR1
WISP2
TPH1
HPVC1
LOC100131655
RN7SKP245
LOC101928844
LOC400748
PLA2G7
LINC01197
RAB30
OLA1P2
CIART
LL22NC01-81G9.3
FUT9
LOC105378231
FLJ37505
LOC100128006
LOC101928716
SLITRK2
LOC101927032
PTCHD1
log(1+tpm)
4
3
2
1
High_Risk_s8
High_Risk_s9
High_Risk_s7
High_Risk_s10
High_Risk_s11
Low_Risk_s1
Low_Risk_s3
High_Risk_s12
Low_Risk_s5
Low_Risk_s4
Low_Risk_s2
Low_Risk_s6

Pathway (GO) analysis for module 5 gene network

pathway_name	logFC	pvalue	FDR	contrast	Module Num.
APOPTOSIS_GO	1.020321243	0.021061086	0.186309606	Low-high	5
IMMUNE_RESPONSE	1.59347154	0.039988506	0.33444932	Low-high	5
DEFENSE_RESPONSE	1.120250187	0.045690737	0.375316771	Low-high	5
INFLAMMATORY_RESPONSE	1.22017913	0.097388047	0.509114689	Low-high	5

Labeling is similar to a.

c

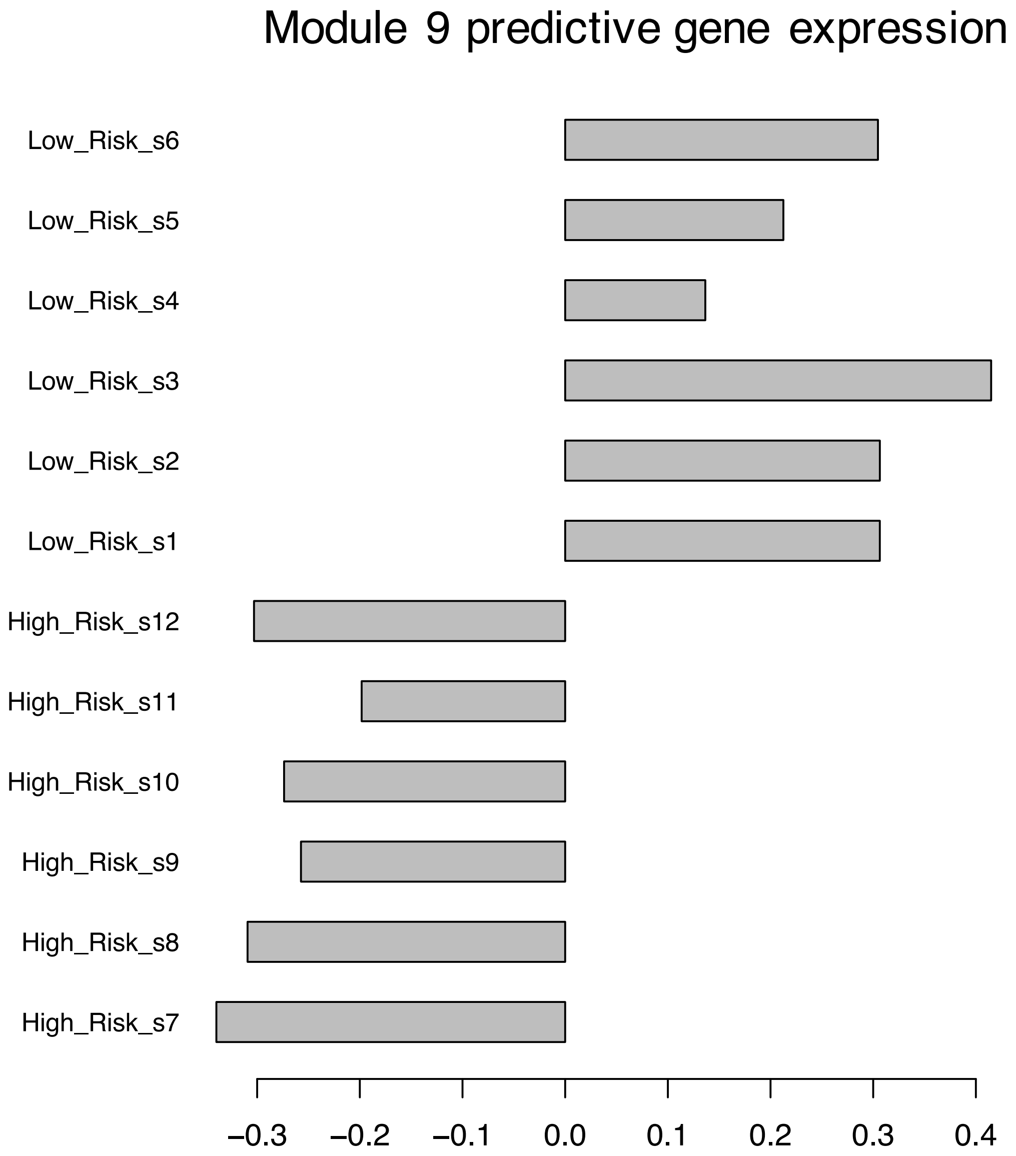

Labeling is similar to a.

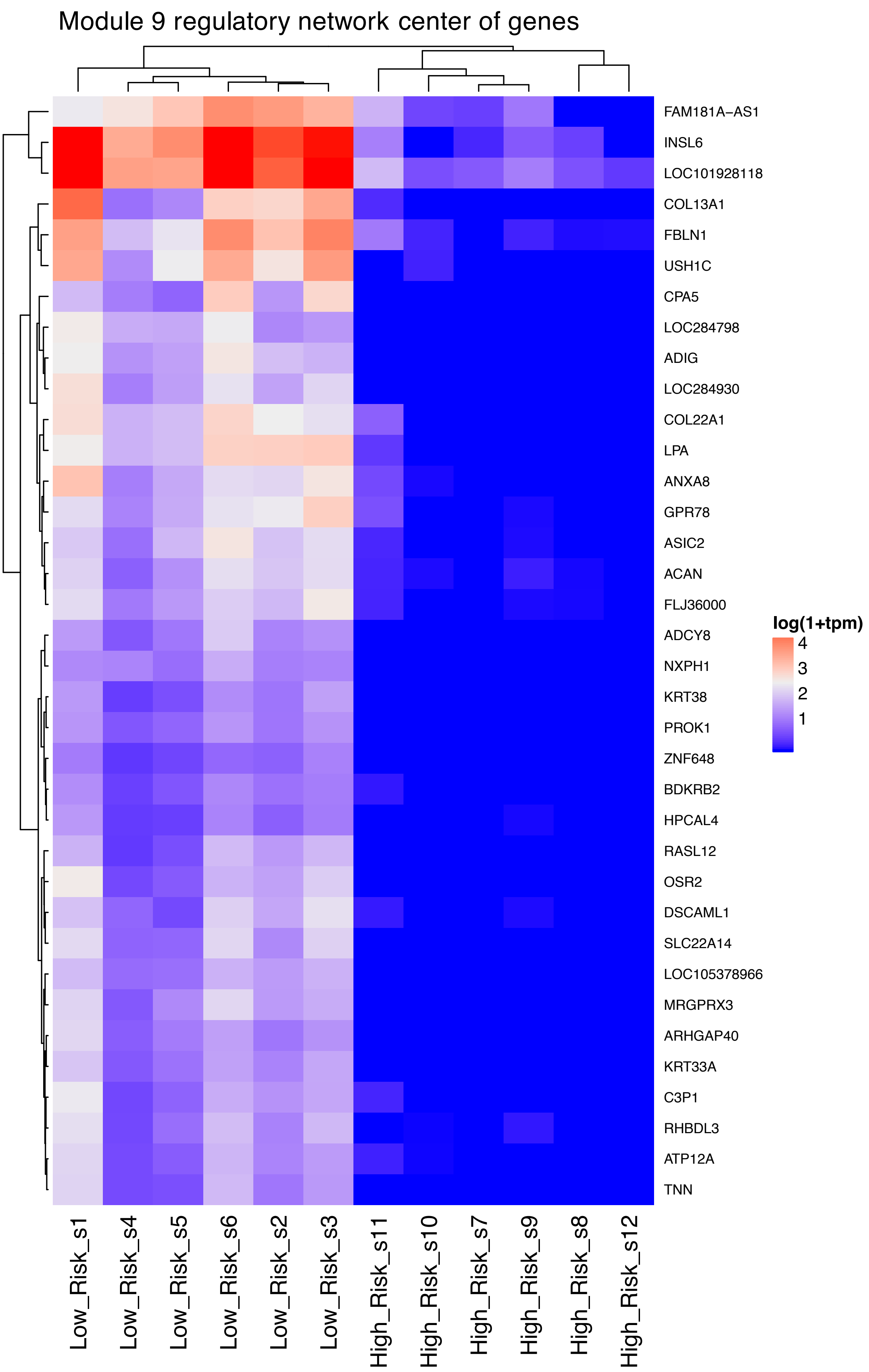

Module 9 regulatory network center of genes
FAM181A−AS1
INSL6
LOC101928118
COL13A1
FBLN1
USH1C
CPA5
LOC284798
ADIG
LOC284930
COL22A1
LPA
ANXA8
GPR78
ASIC2
ACAN
FLJ36000
ADCY8
NXPH1
KRT38
PROK1
ZNF648
BDKRB2
HPCAL4
RASL12
OSR2
DSCAML1
SLC22A14
LOC105378966
MRGPRX3
ARHGAP40
KRT33A
C3P1
RHBDL3
ATP12A
TNN
log(1+tpm)
4
3
2
1
Low_Risk_s1
Low_Risk_s4
Low_Risk_s5
Low_Risk_s6
Low_Risk_s2
Low_Risk_s3
High_Risk_s11
High_Risk_s10
High_Risk_s7
High_Risk_s9
High_Risk_s8
High_Risk_s12

Pathway (GO) analysis for module 9 gene network

pathway_name	logFC	pvalue	FDR	contrast	Module Num.
CYTOKINE_ACTIVITY	3.149111925	1.52E-08	1.52E-06	Low-high	9
APOPTOSIS_GO	2.376391897	8.20E-08	1.76E-06	Low-high	9
IMMUNE_RESPONSE	2.505158622	2.30E-07	2.73E-06	Low-high	9
IMMUNE_SYSTEM_PROCESS	2.493697053	2.31E-07	2.73E-06	Low-high	9
RESPONSE_TO_STRESS	2.516573456	3.24E-07	3.23E-06	Low-high	9
DEFENSE_RESPONSE	2.421098306	3.54E-07	3.46E-06	Low-high	9
RESPONSE_TO_WOUNDING	2.498118094	7.50E-07	5.60E-06	Low-high	9
INFLAMMATORY_RESPONSE	2.32005864	8.81E-07	6.14E-06	Low-high	9
CYTOKINE_PRODUCTION	2.620220089	1.73E-06	9.24E-06	Low-high	9
RESPONSE_TO_BACTERIUM	3.018800782	2.77E-06	1.26E-05	Low-high	9
INDUCTION_OF_APOPTOSIS_BY_INTRACELLULAR_SIGNALS	4.235922519	2.80E-06	1.26E-05	Low-high	9
HUMORAL_IMMUNE_RESPONSE	2.655395616	2.89E-06	1.29E-05	Low-high	9
INNATE_IMMUNE_RESPONSE	2.664409218	6.22E-06	2.26E-05	Low-high	9
RESPONSE_TO_RADIATION	2.554057966	6.47E-06	2.33E-05	Low-high	9
COAGULATION	2.876561145	6.54E-06	2.35E-05	Low-high	9
DEFENSE_RESPONSE_TO_BACTERIUM	3.625395179	6.71E-06	2.39E-05	Low-high	9
VIRAL_REPRODUCTION	1.732878307	7.32E-06	2.55E-05	Low-high	9
CELLULAR_DEFENSE_RESPONSE	2.439907494	8.74E-06	2.90E-05	Low-high	9
IMMUNE_SYSTEM_DEVELOPMENT	2.333044219	1.41E-05	4.23E-05	Low-high	9
APOPTOTIC_PROGRAM	3.052568177	1.49E-05	4.42E-05	Low-high	9
REGULATION_OF_IMMUNE_SYSTEM_PROCESS	2.143797437	1.58E-05	4.68E-05	Low-high	9
WOUND_HEALING	2.967456513	1.84E-05	5.18E-05	Low-high	9
HEMATOPOIETIN_INTERFERON_CLASSD200_DOMAIN_CYTOKINE_RECEPTOR_BINDING	2.876906117	3.19E-05	8.03E-05	Low-high	9
REGULATION_OF_JAK_STAT_CASCADE	3.849428396	3.20E-05	8.03E-05	Low-high	9
RESPONSE_TO_OXIDATIVE_STRESS	2.619973909	3.23E-05	8.11E-05	Low-high	9
RESPONSE_TO_DNA_DAMAGE_STIMULUS	2.476437403	3.26E-05	8.15E-05	Low-high	9
BLOOD_COAGULATION	2.852439192	3.98E-05	9.57E-05	Low-high	9
JAK_STAT_CASCADE	2.704919656	4.08E-05	9.78E-05	Low-high	9
STRESS_ACTIVATED_PROTEIN_KINASE_SIGNALING_PATHWAY	1.800596129	4.93E-05	0.000114434	Low-high	9
RESPONSE_TO_HYPOXIA	2.757428697	5.64E-05	0.000127711	Low-high	9
CYTOCHROME_C_OXIDASE_ACTIVITY	4.605622934	7.44E-05	0.00016092	Low-high	9
RESPONSE_TO_UV	3.509670956	7.91E-05	0.000170216	Low-high	9
REGULATION_OF_INTERFERON_GAMMA_BIOSYNTHETIC_PROCESS	2.868419275	0.000119803	0.000241469	Low-high	9
INTERFERON_GAMMA_PRODUCTION	2.868419275	0.000119803	0.000241469	Low-high	9
ACUTE_INFLAMMATORY_RESPONSE	2.724700541	0.000125692	0.000250999	Low-high	9
IMMUNE_EFFECTOR_PROCESS	1.935258827	0.000142883	0.000277807	Low-high	9
POSITIVE_REGULATION_OF_IMMUNE_RESPONSE	2.297047641	0.0002177	0.000397268	Low-high	9
CELL_STRUCTURE_DISASSEMBLY_DURING_APOPTOSIS	2.977906763	0.00022978	0.000418218	Low-high	9
ANTIGEN_BINDING	3.150384306	0.00025695	0.000462245	Low-high	9
ACTIVATION_OF_IMMUNE_RESPONSE	2.503878601	0.000280742	0.000497987	Low-high	9
RESPONSE_TO_VIRUS	1.824262382	0.000361305	0.000621182	Low-high	9
PHAGOCYTOSIS	3.161327968	0.000361318	0.000621182	Low-high	9
DNA_REPAIR	1.95561919	0.000934908	0.00145662	Low-high	9
APOPTOTIC_NUCLEAR_CHANGES	2.714923416	0.001033464	0.001597693	Low-high	9
CELLULAR_RESPONSE_TO_STRESS	5.388873715	0.001443034	0.002133978	Low-high	9
ADAPTIVE_IMMUNE_RESPONSE_GO_0002460	1.960544764	0.001897165	0.002744499	Low-high	9
IMMUNOLOGICAL_SYNAPSE	3.873343927	0.002494184	0.003545704	Low-high	9
DNA_DAMAGE_RESPONSESIGNAL_TRANSDUCTION_BY_P53_CLASS_MEDIATOR	4.225210373	0.00294576	0.004111756	Low-high	9

Labeling is similar to a.

d

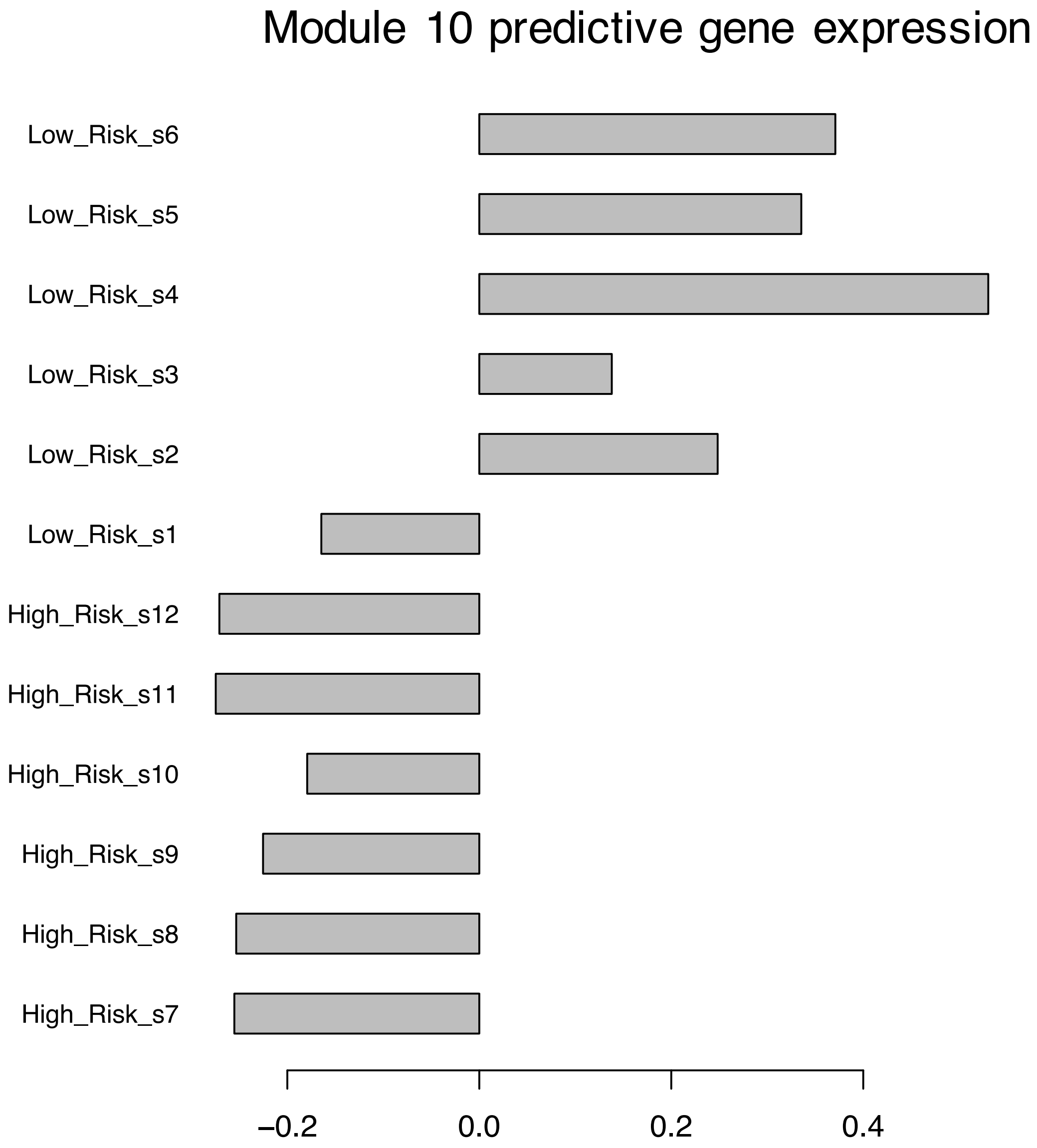

Labeling is similar to a.

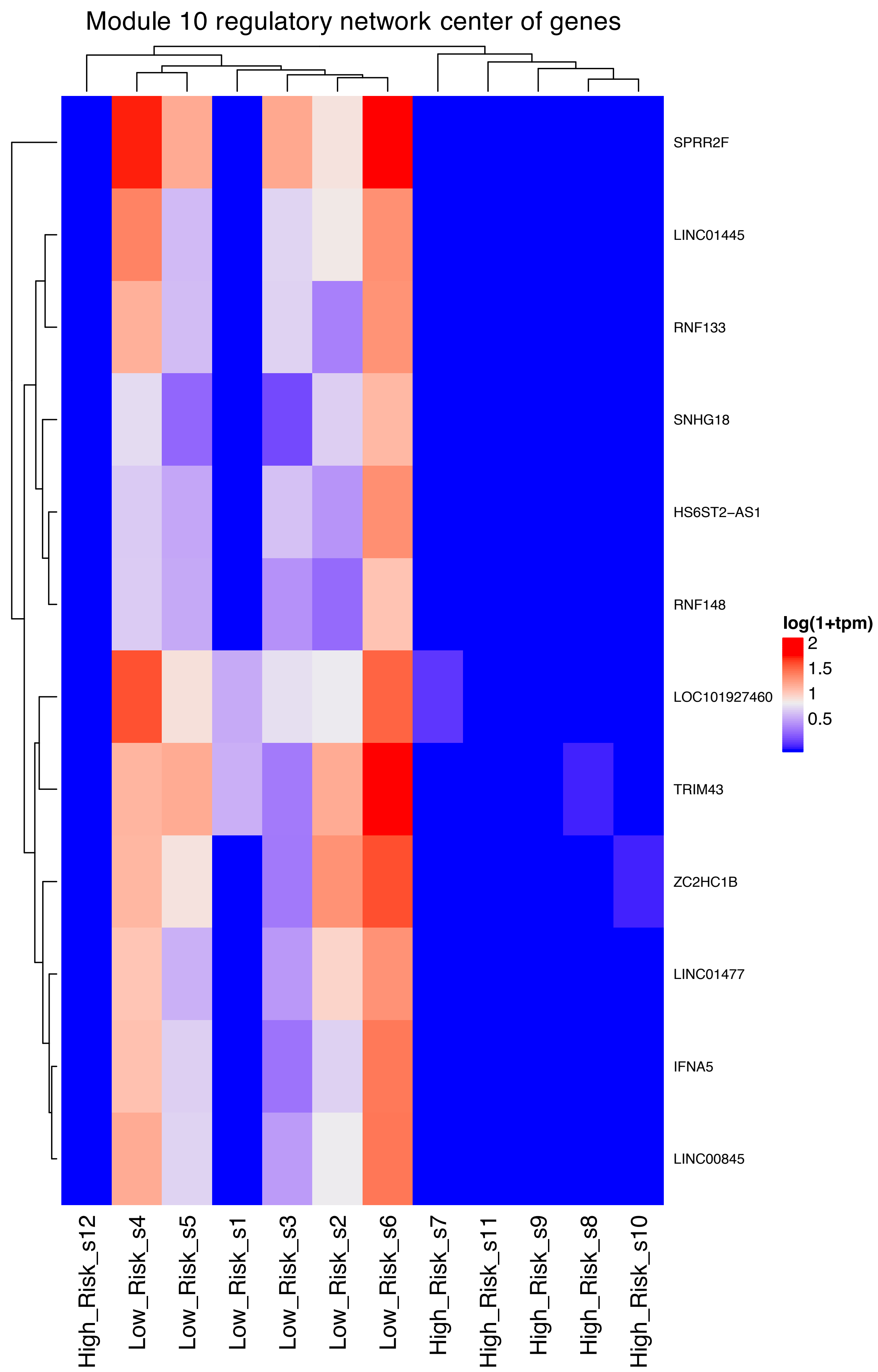

Module 10 regulatory network center of genes
SPRR2F
LINC01445
RNF133
SNHG18
HS6ST2-AS1
RNF148
LOC101927460
TRIM43
ZC2HC1B
LINC01477
IFNA5
LINC00845
log(1+tpm)
2
1.5
1
0.5
High_Risk_s12
Low_Risk_s4
Low_Risk_s5
Low_Risk_s1
Low_Risk_s3
Low_Risk_s2
Low_Risk_s6
High_Risk_s7
High_Risk_s11
High_Risk_s9
High_Risk_s8
High_Risk_s10

Pathway (GO) analysis for module 10 gene network

pathway_name	logFC	pvalue	FDR	contrast	Module Num.
CYTOKINE_ACTIVITY	4.871967009	3.44E-05	0.000616423	Low-high	10
IMMUNE_SYSTEM_PROCESS	4.37768099	0.00010714	0.001171556	Low-high	10
PROGRAMMED_CELL_DEATH	5.925965601	0.00015395	0.00158034	Low-high	10
APOPTOSIS_GO	5.925965601	0.00015395	0.00158034	Low-high	10
CELLULAR_DEFENSE_RESPONSE	4.801160428	0.000386922	0.002659068	Low-high	10
NEGATIVE_REGULATION_OF_DEVELOPMENTAL_PROCESS	7.375178229	0.000449345	0.002659068	Low-high	10
IMMUNE_RESPONSE	4.29588445	0.000700231	0.003957483	Low-high	10
RESPONSE_TO_STRESS	4.020736847	0.001081136	0.005493045	Low-high	10
DEFENSE_RESPONSE	3.919330541	0.001297406	0.006525953	Low-high	10
REGULATION_OF_PROGRAMMED_CELL_DEATH	5.618485929	0.001667465	0.007838645	Low-high	10
RESPONSE_TO_VIRUS	5.252567884	0.002002563	0.008864694	Low-high	10
RESPONSE_TO_OTHER_ORGANISM	5.252567884	0.002002563	0.008864694	Low-high	10
RESPONSE_TO_HYPOXIA	6.186225532	0.004697166	0.01238444	Low-high	10
ENDONUCLEASE_ACTIVITY	6.186225532	0.004697166	0.01238444	Low-high	10
INNATE_IMMUNE_RESPONSE	4.224545978	0.006879002	0.015945336	Low-high	10
CHEMOKINE_ACTIVITY	3.991447947	0.007463805	0.016538739	Low-high	10
RESPONSE_TO_WOUNDING	3.667175937	0.012052157	0.024643232	Low-high	10
WOUND_HEALING	4.249417598	0.013170871	0.026082473	Low-high	10
INFLAMMATORY_RESPONSE	3.473095383	0.025626583	0.046384795	Low-high	10
ANTIGEN_BINDING	4.165113928	0.034277227	0.055797558	Low-high	10
RNA_HELICASE_ACTIVITY	-1.95909558	0.045255131	0.070041019	Low-high	10
HELICASE_ACTIVITY	-1.95909558	0.045255131	0.070041019	Low-high	10
REGULATION_OF_DEFENSE_RESPONSE	3.861793629	0.048929332	0.070722569	Low-high	10
IMMUNE_SYSTEM_DEVELOPMENT	4.317882266	0.062490194	0.086353208	Low-high	10
NF_KAPPAB_BINDING	-0.980726207	0.072005886	0.09616955	Low-high	10
VIRAL_INFECTIOUS_CYCLE	3.959576008	0.082790264	0.103074881	Low-high	10

Labeling is similar to A.

e

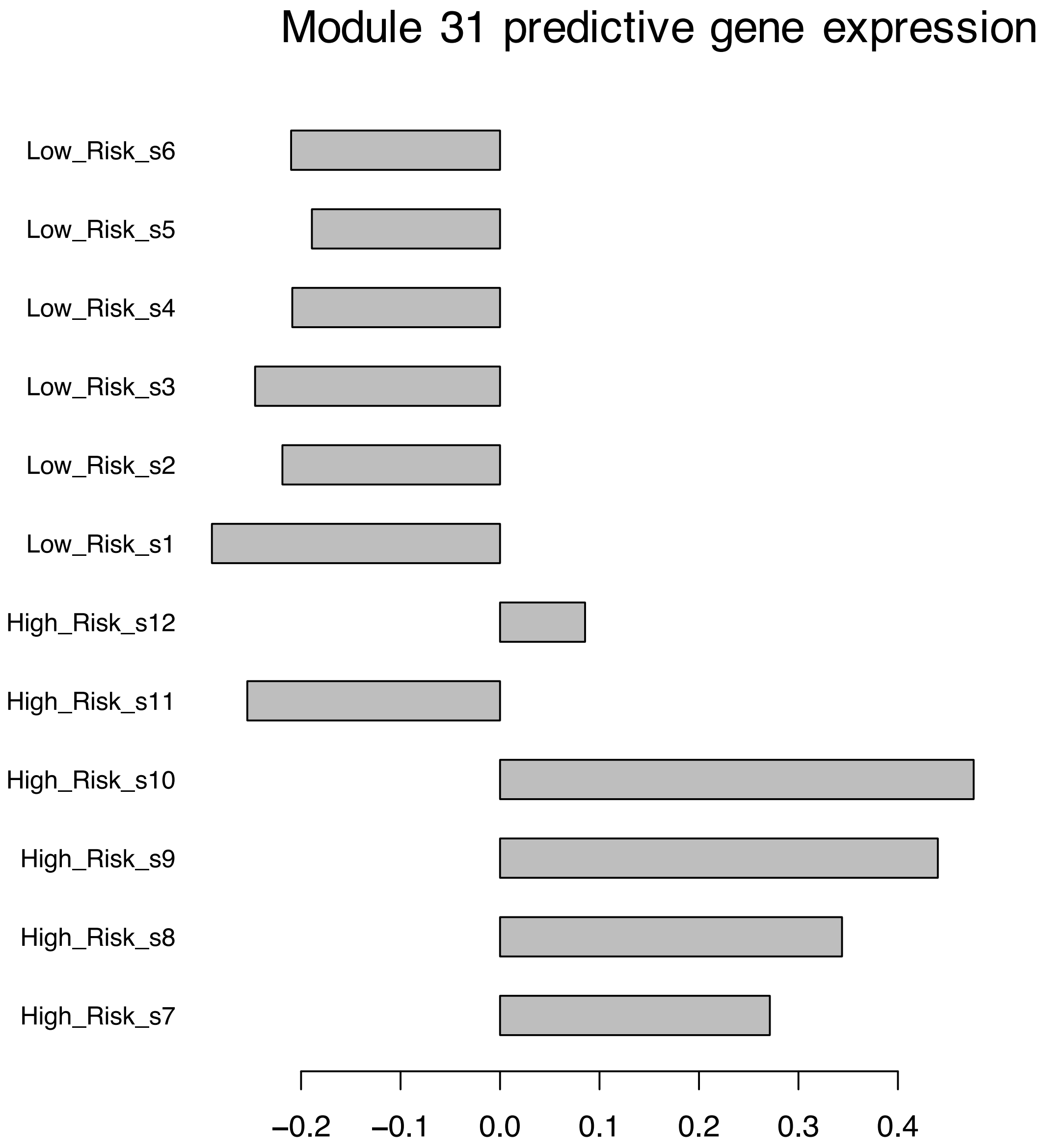

Labeling is similar to a.

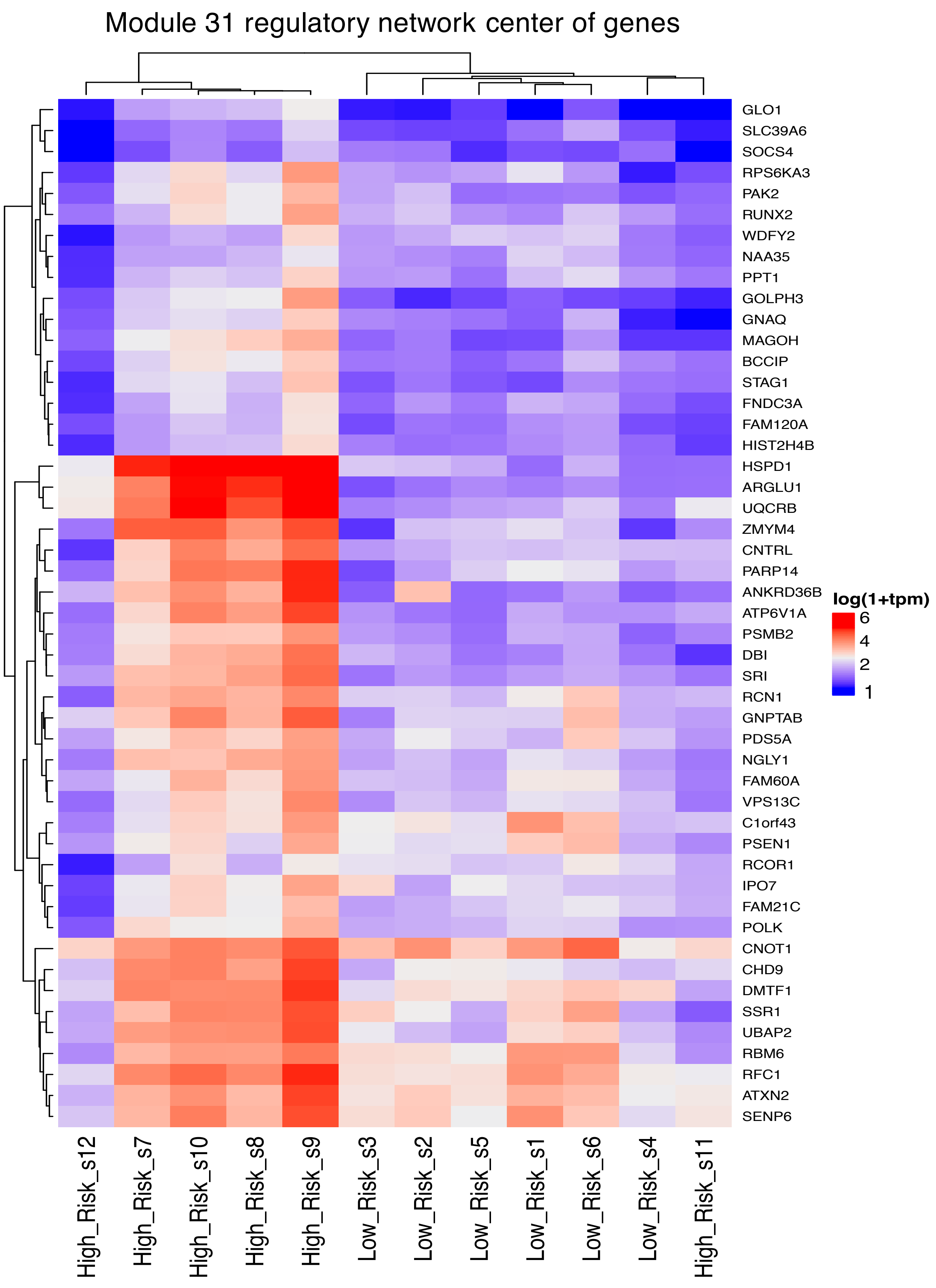

The regulatory network center of genes for module 31 had signed connectivity scores (Horvath, et.al) greater than or equal to 0.9862.

Module 31 regulation gene members

ENSEMBL Gene ID	Gene Name	Signed Connectivity Score
ENSG00000135829	DHX9	0.974568774
ENSG00000005893	LAMP2	0.972069149
ENSG00000067248	DHX29	0.964858505
ENSG00000108406	DHX40	0.962452119
ENSG00000174953	DHX36	0.959186239
ENSG00000100697	DICER1	0.957078805
ENSG00000166783	KIAA0430	0.948546451
ENSG00000080824	HSP90AA1	0.929781232
ENSG00000109606	DHX15	0.929101217
ENSG00000277140	KIAA0430	0.866507564
ENSG00000140829	DHX38	0.865619762
ENSG00000132153	DHX30	0.8422372
ENSG00000067596	DHX8	0.840604699
ENSG00000089876	DHX32	0.840010029
ENSG00000057663	ATG5	0.83750447
ENSG00000163214	DHX57	0.606158448

Regulation genes in Figure 7 held high values for the signed eigen-based connectivity score (Horvath et.al) which indicates that the regulating genes in Figure 7 held membership within the same module (31).
Note many of these are missing from the previous central-hub network heatmap because the center hub genes had higher more competitive signed connectivity scores.

Pathway (GO) analysis for module 31 gene network

Pathways Activated in Module 31 Comparing Blast to LSC	logFC	pvalue	FDR
AGING	-2.259053278	4.40E-08	1.65E-05
DEFENSE_RESPONSE_TO_VIRUS	-0.970655966	1.77E-06	0.000105709
DOUBLE_STRANDED_RNA_BINDING	-1.005865775	4.29E-06	0.000172675
RNA_POLYMERASE_II_TRANSCRIPTION_FACTOR_ACTIVITY	-0.804627105	8.74E-06	0.000230038
ATP_DEPENDENT_DNA_HELICASE_ACTIVITY	-1.583058938	9.43E-06	0.000239518
HELICASE_ACTIVITY	-0.6725089	1.61E-05	0.000295869
RESPONSE_TO_HYPOXIA	-1.022390426	7.77E-05	0.000715813
RESPONSE_TO_VIRUS	-0.685949777	7.85E-05	0.000715813
IMMUNE_EFFECTOR_PROCESS	-0.477567852	0.000111877	0.000913866
T_CELL_PROLIFERATION	-0.727621916	0.000127639	0.001008346
RESPONSE_TO_IONIZING_RADIATION	1.004579964	0.00012913	0.00101449
NF_KAPPAB_BINDING	-1.330958805	0.000268586	0.001682506
VIRAL_REPRODUCTION	-0.544314726	0.000347636	0.002025976
RESPONSE_TO_BACTERIUM	0.997368983	0.000620268	0.002982644
DEFENSE_RESPONSE_TO_BACTERIUM	0.997368983	0.000620268	0.002982644
DNA_HELICASE_ACTIVITY	-0.630402928	0.000719008	0.003341273
PROGRAMMED_CELL_DEATH	-0.463947952	0.00215101	0.007666005
APOPTOSIS_GO	-0.463947952	0.00215101	0.007666005
REGULATION_OF_APOPTOSIS	-0.464765726	0.004440405	0.013637702
POSITIVE_REGULATION_OF_IMMUNE_SYSTEM_PROCESS	0.258560404	0.010857385	0.027133921
INNATE_IMMUNE_RESPONSE	0.675039146	0.01314124	0.031565613
REGULATION_OF_CYTOKINE_PRODUCTION	-0.579974571	0.02218741	0.0473885
ATP_DEPENDENT_RNA_HELICASE_ACTIVITY	-0.330678424	0.038473282	0.075149734
RESPONSE_TO_UV	0.366311047	0.044378622	0.084706577
REGULATION_OF_IMMUNE_SYSTEM_PROCESS	0.405387385	8.59E-05	0.001454398
IMMUNE_EFFECTOR_PROCESS	-0.477567852	0.000111877	0.001454398
POSITIVE_REGULATION_OF_IMMUNE_SYSTEM_PROCESS	0.258560404	0.010857385	0.085418059
INNATE_IMMUNE_RESPONSE	0.675039146	0.01314124	0.085418059
PRODUCTION_OF_MOLECULAR_MEDIATOR_OF_IMMUNE_RESPONSE	-0.412408496	0.019600274	0.101921423
GO_INFLAMMATORY_RESPONSE	-0.326191809	0.042640909	0.16663155
Inflammation (combined gene set of 649 genes)	-0.322024251	0.046462256	0.16663155

Labeling is similar to a.

Supplement figure 8

a

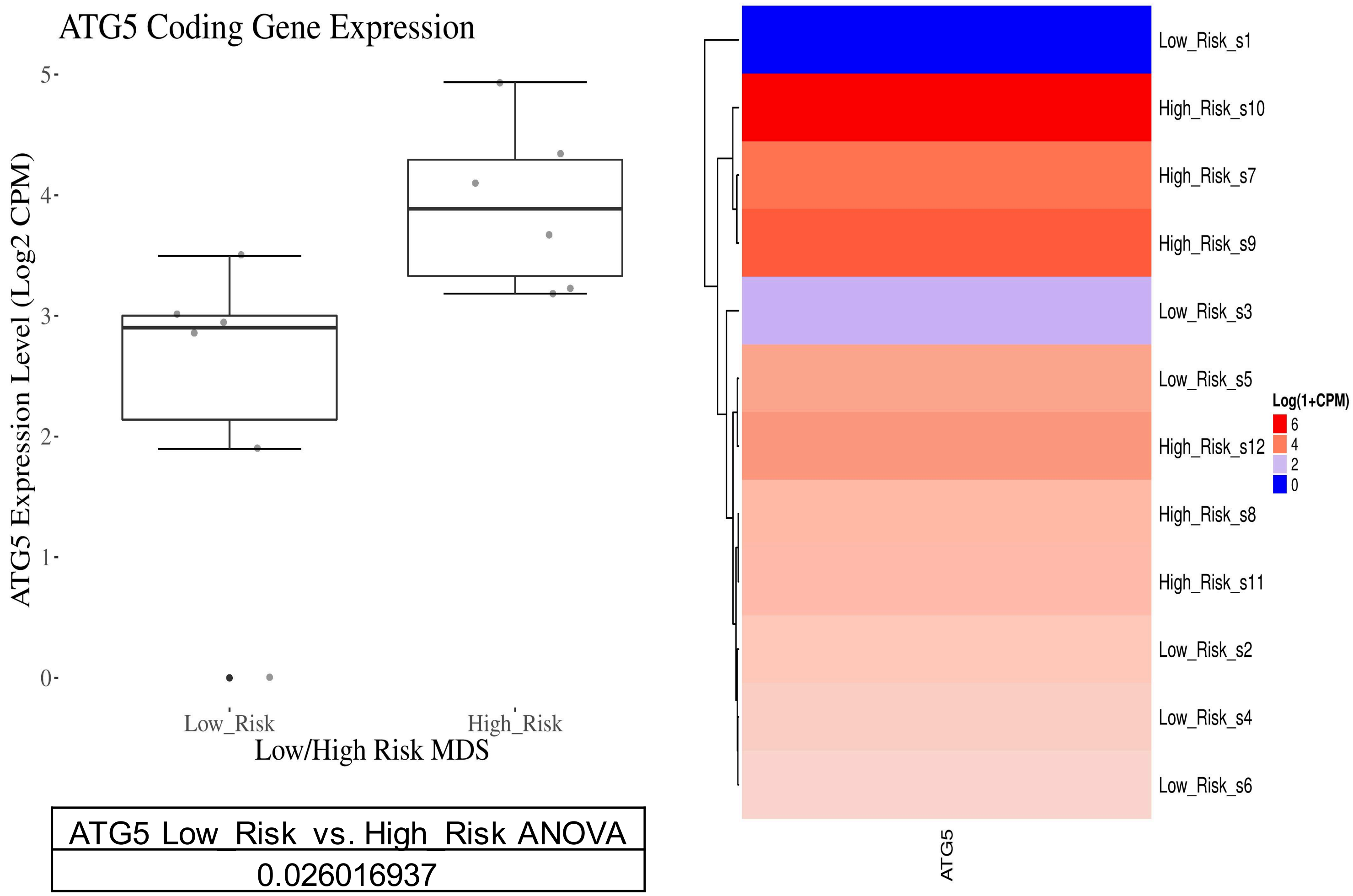

ATG5 Low_Risk vs. High_Risk ANOVA
0.026016937

ANOVA of ATG5 in AML

ANOVA p.values	pHSC-Blast	Blast-LSC	pHSC-LSC
0.001961336	0.348911789	0.0012036	0.039891377

The box plot and corresponding heatmap analyze the expression of ATG5 In units of log base 2 CPM which includes an ANOVA comparing means of sample groups Low-Risk compared to High-Risk expression of ATG5. The ANOVA corresponds to the ATG5 expression levels in each individual patient across stages of AML. An ANOVA is conducted testing the mean expression of ATG5 with paired patient data. The p-values are reported using Bonferroni adjustments for multiple testing.

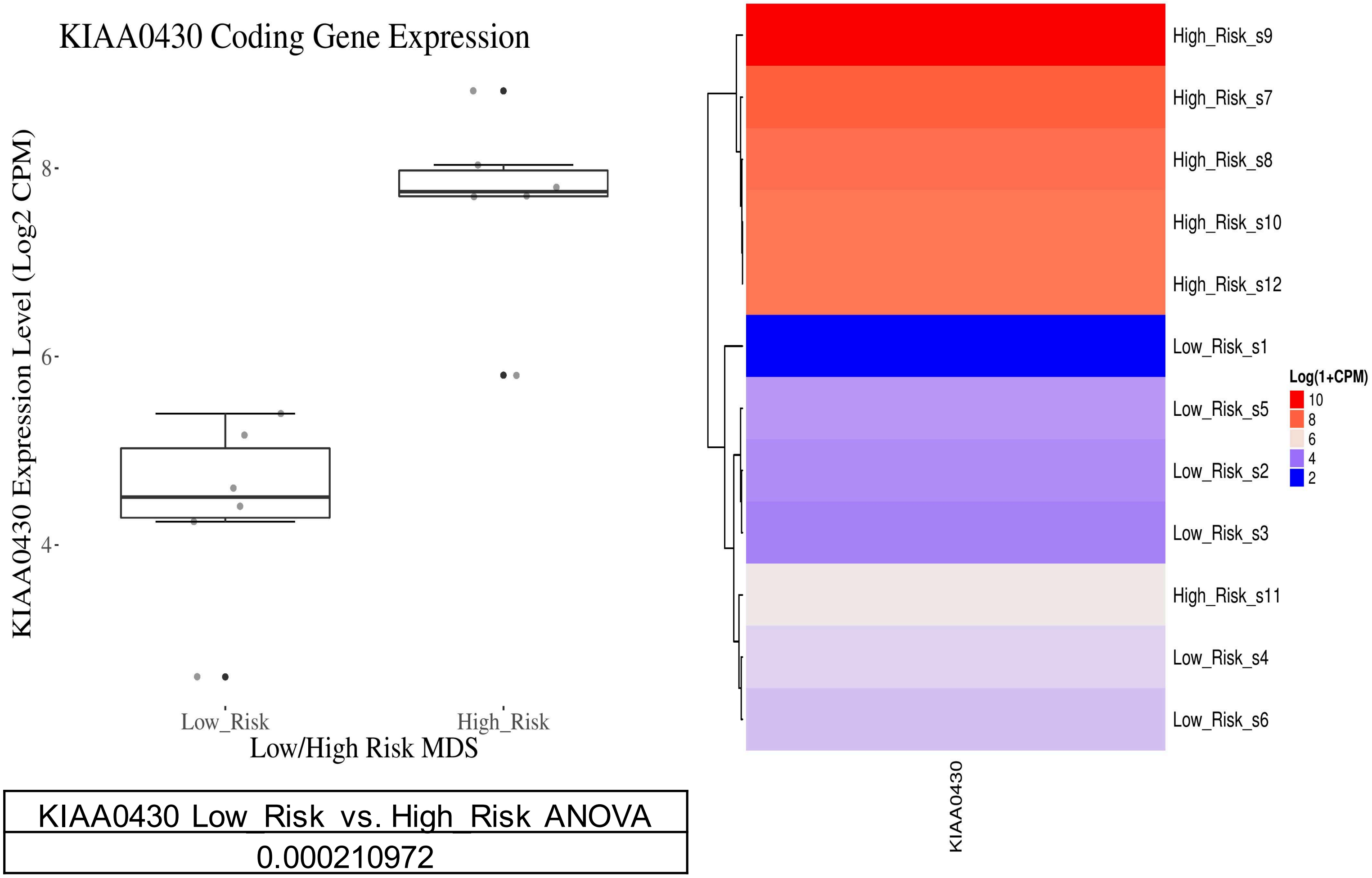

KIAA0430 Low_Risk vs. High_Risk ANOVA
0.000210972

ANOVA of KIAA0430 in AML

ANOVA	pHSC-Blast	Blast-LSC	pHSC-LSC
0.020774852	1	0.023088174	0.104627133

Similar to a, except with respect to KIAA0430 gene.

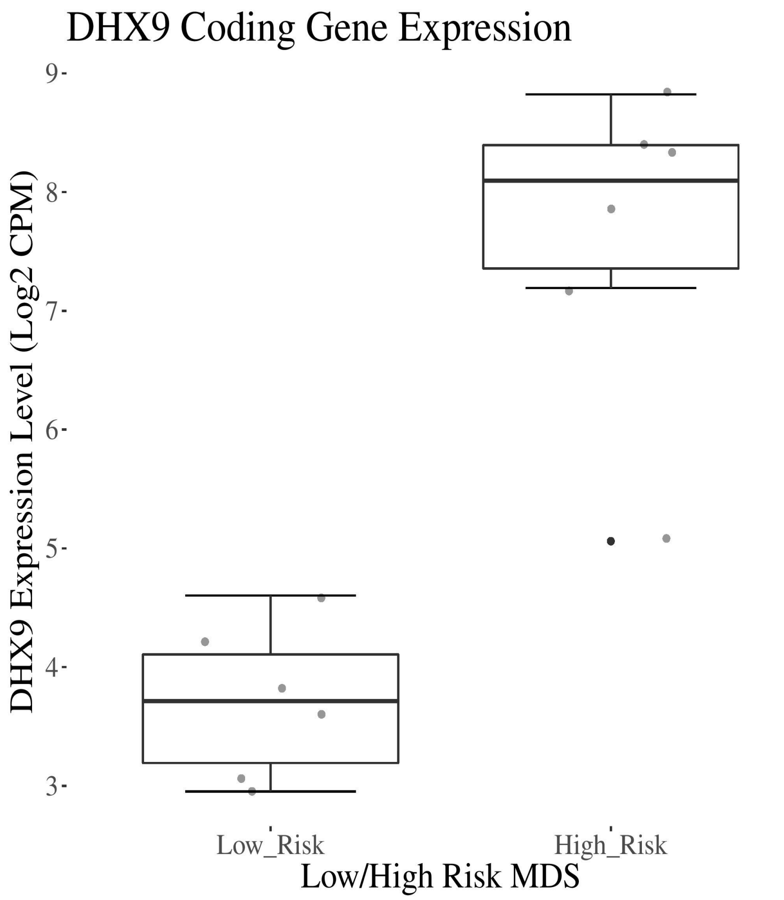

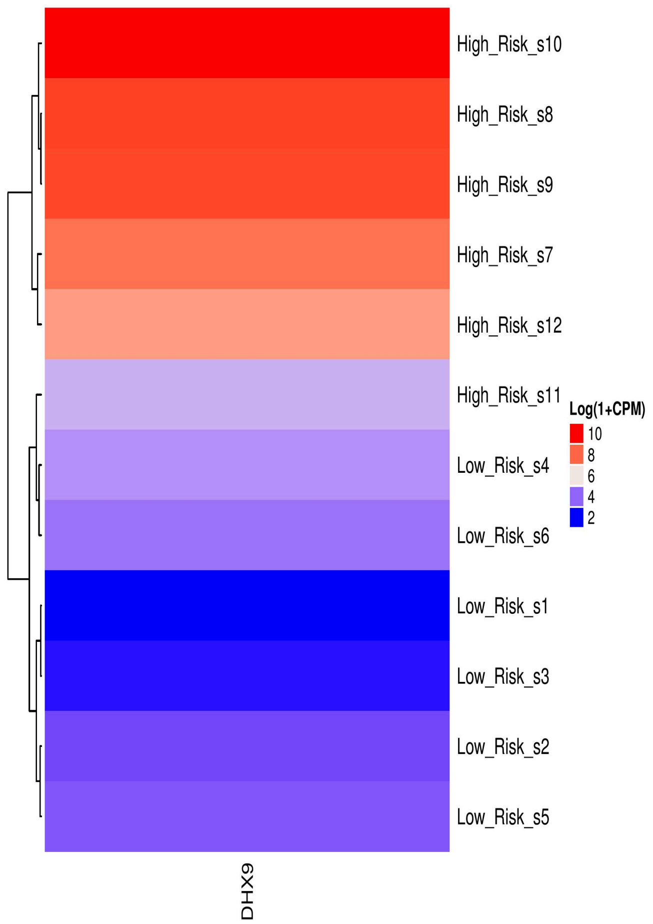

DHX9 Low_Risk vs High_Risk ANOVA
8.64E-05

ANOVA of DHX9 in AML

ANOVA	pHSC-Blast	Blast-LSC	pHSC-LSC
0.009166885	0.461839918	0.008193754	0.303978086

Similar to a, except with respect to DHX9 gene.

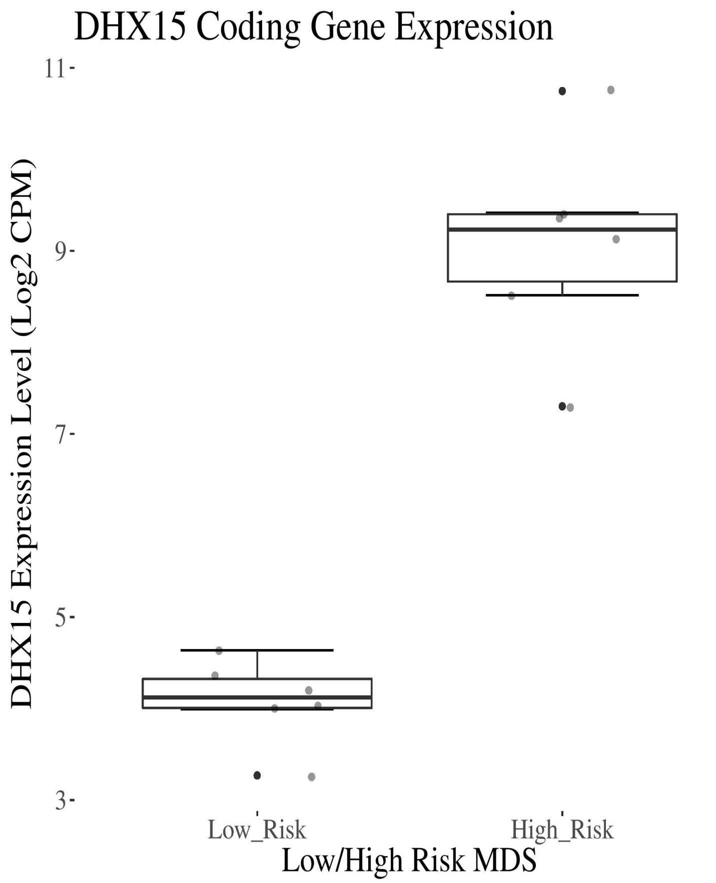

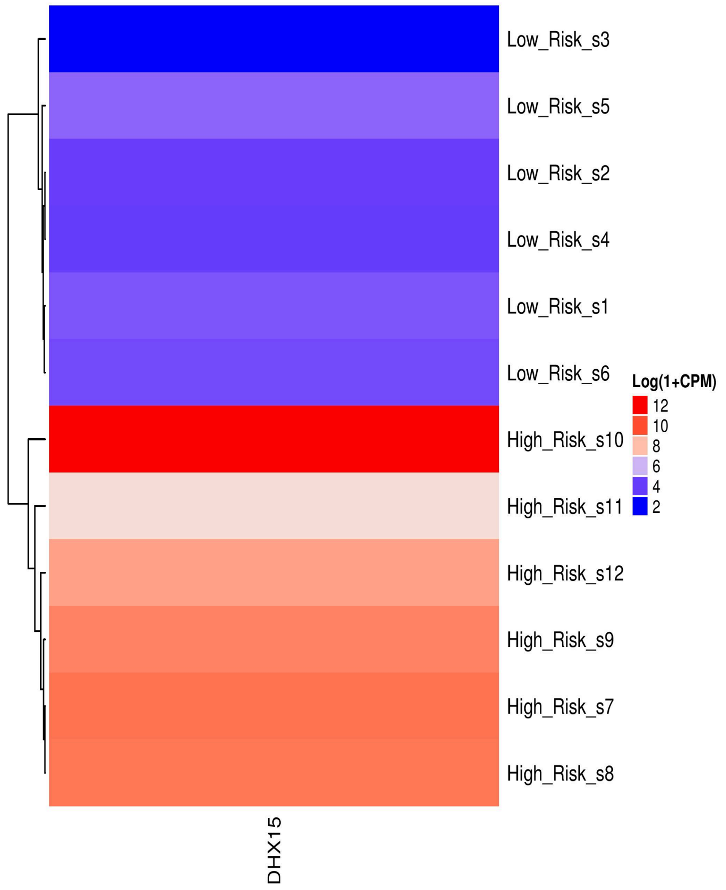

DHX15_Low_Risk_vs. High_Risk_ANOVA
1.64E-06

ANOVA of DHX15 in AML

ANOVA	pHSC-Blast	Blast-LSC	pHSC-LSC
0.000192815	0.824152896	0.000147431	0.025271945

Similar to a, except with respect to DHX15 gene.

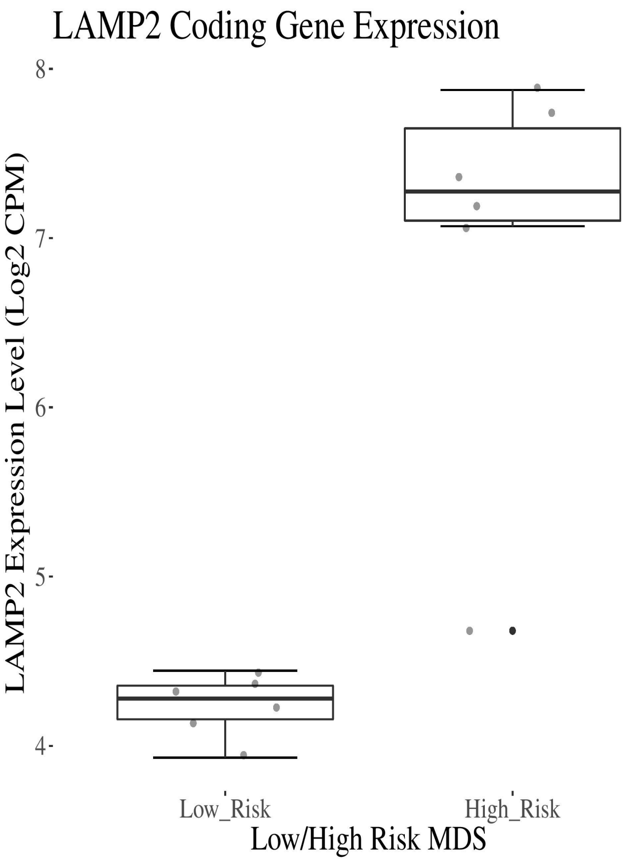

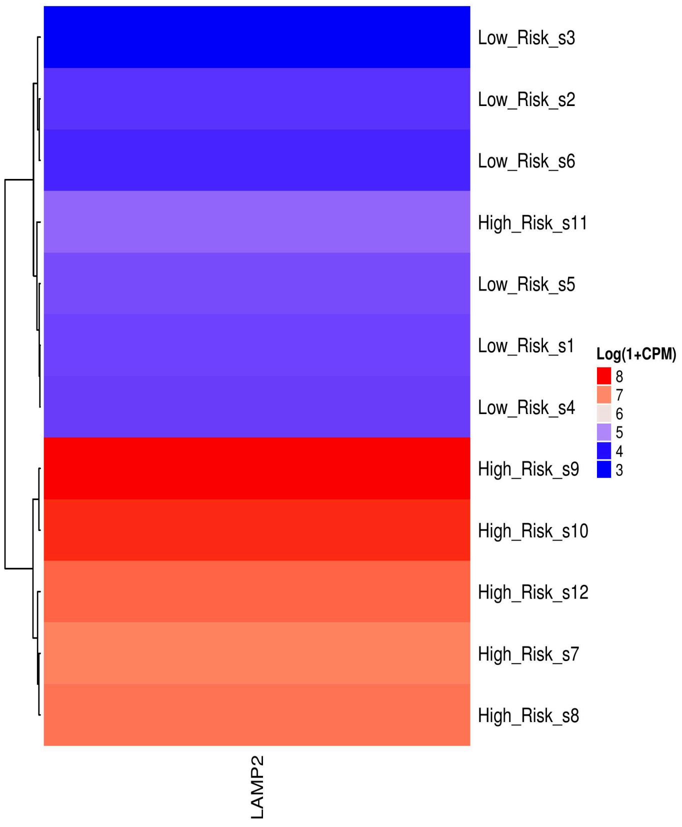

LAMP2 Low_Risk vs. High_Risk ANOVA
0.000205484

ANOVA of LAMP2 in AML

ANOVA	pHSC-Blast	Blast-LSC	pHSC-LSC
2.21E-07	0.002955097	0.009213716	8.13E-06

Similar to a, except with respect to LAMP2 gene.

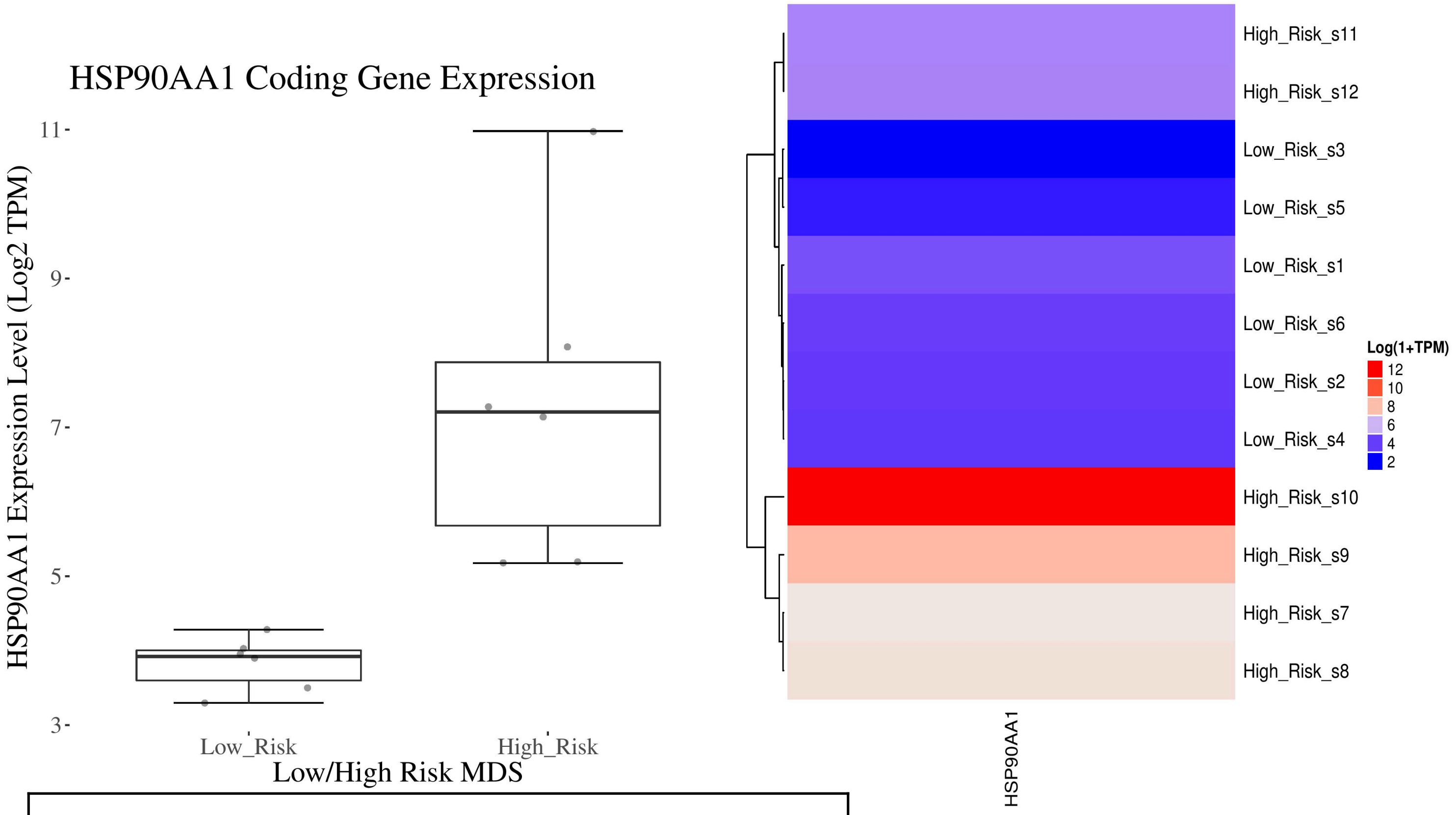

HSP90AA1_Low_Risk_vs_High_Risk_ANOVA
0.002906061

ANOVA of HSP90AA1 in AML

ANOVA	pHSC-Blast	Blast-LSC	pHSC-LSC
0.000847985	1	0.013914731	0.000420838

Similar to a, except with respect to HSP90AA1 gene.

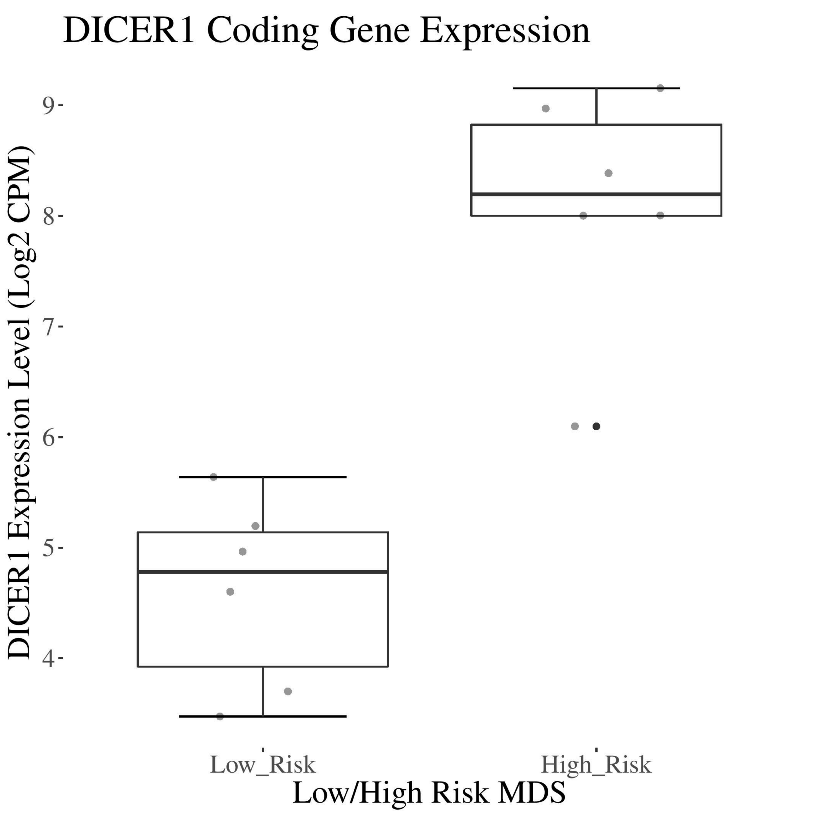

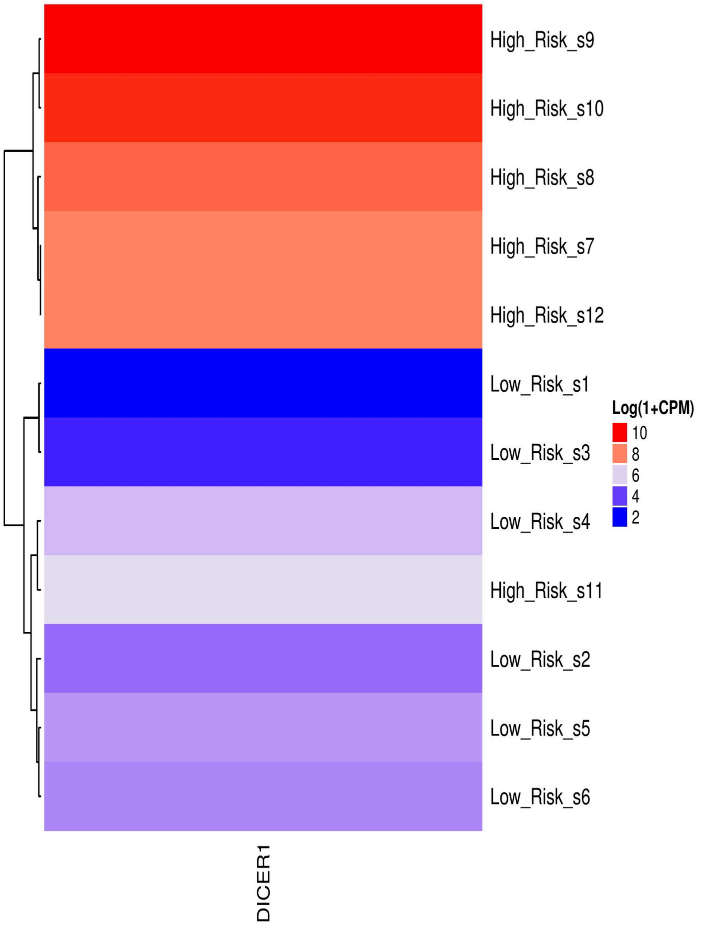

DICER1 Low Risk vs. High Risk ANOVA
0.000102798

ANOVA of DICER1 in AML

ANOVA	pHSC-Blast	Blast-LSC	pHSC-LSC
0.01261824	0.043955414	0.723642012	0.063328119

Similar to a, except with respect to DICER1 gene.

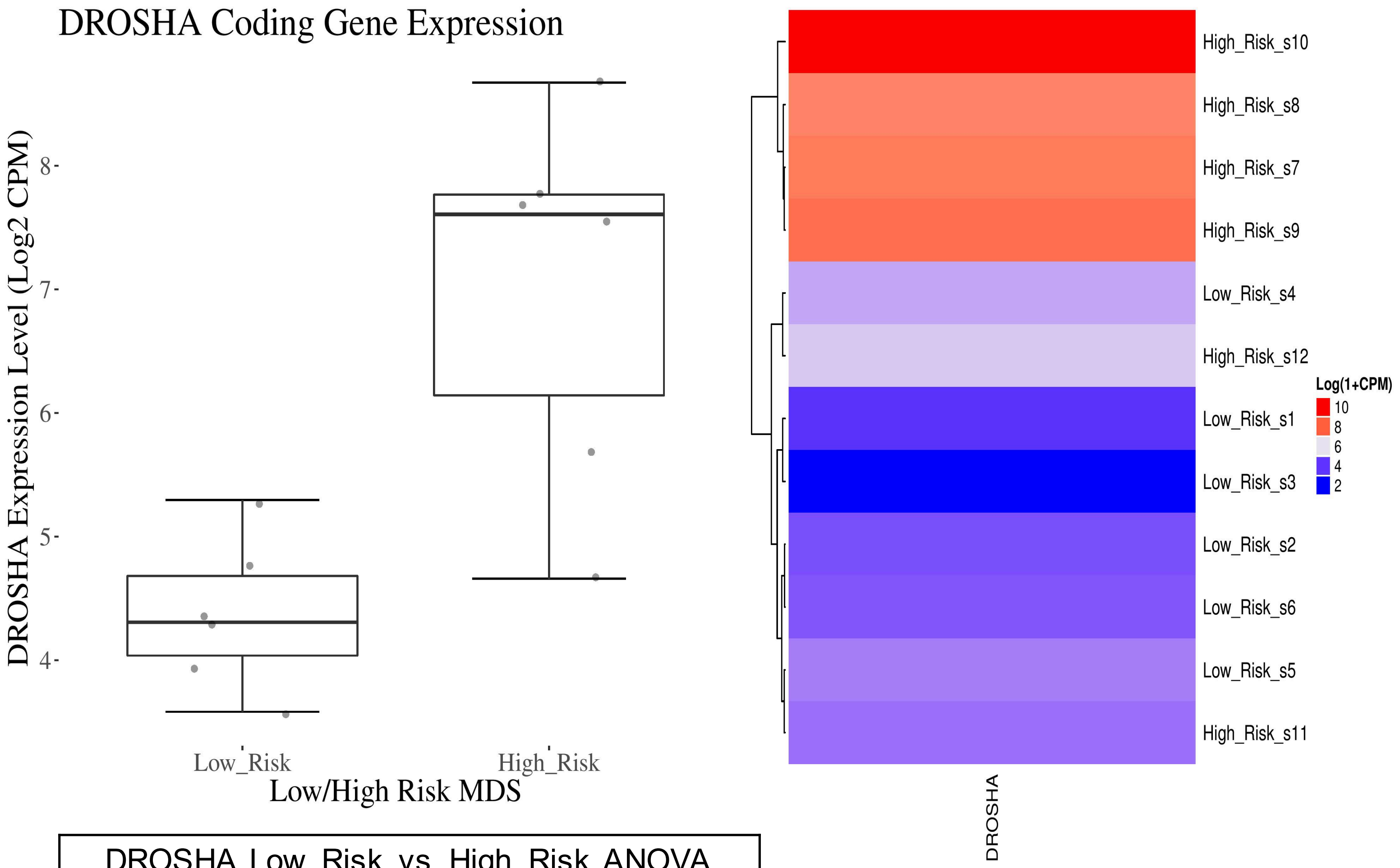

DROSHA Low Risk vs. High Risk ANOVA
0.002701452

ANOVA of DROSHA in AML

ANOVA	pHSC-Blast	Blast-LSC	pHSC-LSC
0.426773345	1	0.985510139	1

Similar to a, except with respect to DROSHA gene.

b

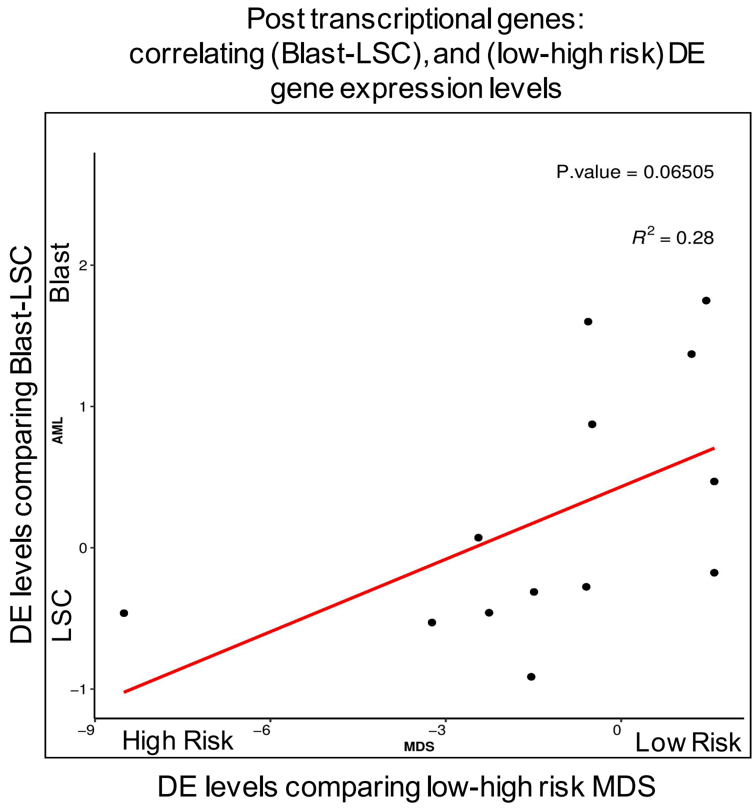
Post transcriptional genes:
correlating (Blast-LSC), and (low-high risk) DE
gene expression levels
P.value = 0.06505
R^2 = 0.28
DE levels comparing Blast-LSC
Blast
LSC
AML
2
1
0
−1
−9
−6
−3
0
High Risk
MDS
Low Risk
DE levels comparing low-high risk MDS

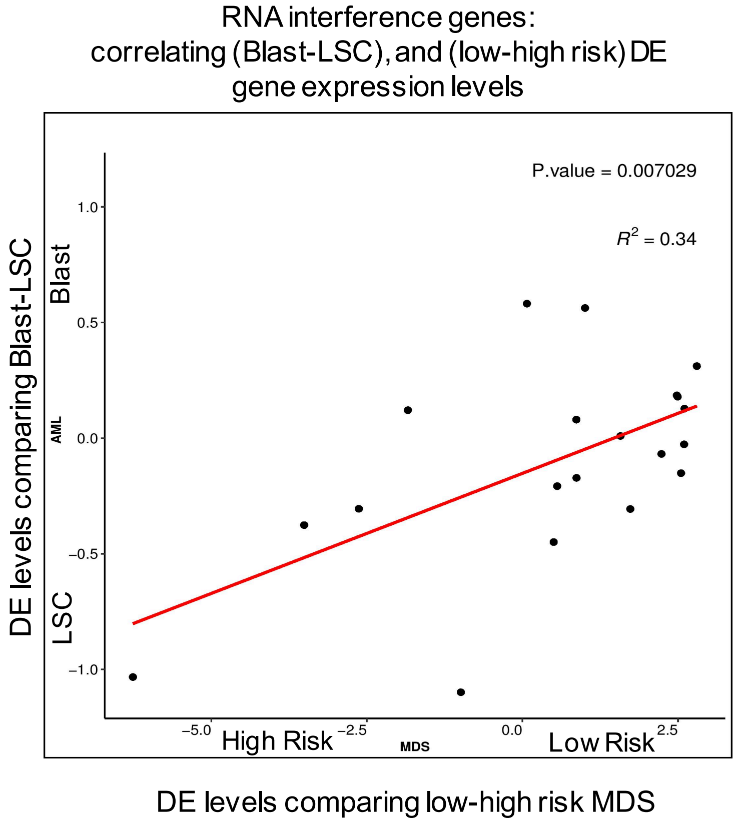
RNA interference genes:
correlating (Blast-LSC), and (low-high risk) DE
gene expression levels
P.value = 0.007029
R^2 = 0.34
DE levels comparing Blast-LSC
Blast
LSC
AML
1.0
0.5
0.0
−0.5
−1.0
−5.0
−2.5
0.0
2.5
High Risk
MDS
Low Risk
DE levels comparing low-high risk MDS
