# Supplementary material for: Suppression of Transposable Elements in Leukemic Stem Cells
Source: Sci Rep. 2017 Aug 1;7:7029. doi: 10.1038/s41598-017-07356-9 (PMC5539300; doi:10.1038/s41598-017-07356-9)
Supplement: Supplementary file 9 — Supplemental Methods [file 41598_2017_7356_MOESM9_ESM.pdf]

Supplemental Methods

Acquisition of RNA sequencing fastq files for AML and MDS

AML RNA sequencing fastq files were analyzed from Corces et al [1] . The MDS fastq files were analyzed from Wang et al [2] . The MDS raw fastq files did not pass basic read quality (Phred) scores of a minimum of 30. We hence used UC Davis Sickle (https://github.com/najoshi/sickle) quality trimmer with paired end trimming, requiring a quality of 27 in a given window. The default length threshold was set to 20. The quality type used was integer quality type score of 1.9, reserved for CASAVA sequencer instruments. We picked 5 MDS samples at random and performed a quality analysis using Babraham Bioinformatics tool FastQC (http://www.bioinformatics.babraham.ac.uk/projects/fastqc/); all 5 samples had quality greater than 30 for both reads. All of the AML RNA sequencing data had read quality scores (Phred) of at least 30.

Differential Expression of Transposable Elements

Transcript abundances were calculated using Kallisto [3] , which quantified the transcript abundances against a merged transcriptome consisting of ERCC (Ambion) Spike-Ins version 97, ENSEMBL Gene Annotations build 84, the repeat transcriptome annotations derived from RepBase [4] release 2103 for Homo sapiens. In each transcript expression analysis (Figure 1A, Figure 5A and Supplemental Figure 1) we fit the annotated repeat expression data to a linear model using limma/voom [5] and between sample normalisation using edgeR [6]. The top differentially expressed (DE) transposable elements (TE) were filtered using a minimum log-fold-change threshold of 1, a significance threshold of 0.05, and minimum read cutoff of 1. The DE TE were then plotted in units of transcript per million (TPM), however the top DE TE were calculated using units in CPM. The row-legends, which identified annotation type and class, used the software Arkas (Colombo, et.al, http://biorxiv.org/content/early/2016/05/20/031435) which tabulated the annotation data into a figure.

We measured absolute TE dysregulation by summarising the absolute fold-changes of the DE TE (Figure 1A) to describe the absolute dysregulation between the samples (Figure 1B).

The analysis of dysregulation of TE in individual AML patient samples, which tracked the stages of AML, utilized an ANOVA with pairwise t-tests. The ANOVA across TE types calculated in units of TPM, as the between sample normalisation, for each target TE type, and calculated an one-way ANOVA of clonal stages as the group factor, and used a pairwise t-test with Bonferroni adjusted p-values.

The comparisons of the AML stages used no FDR filtering for calling DE TE. The comparisons of MDS used no FDR filtering for DE TE elements.

Differential Expression of Coding Genes

The coding gene differential expression was quantified similarly as the TE. The coding gene DE for AML samples used FDR filtering of 'Benjamini Hochberg'. For the analysis of MDS, the FDR filtering used 'Holm' p-value adjustment method.

Gene-Set Analysis Pathway Selection For Comparing Stages of AML

For the gene-set enrichment activity measurements we used curated gene-sets from MsigDB available at GSEA [7] ; we combined 17 canonical immune gene-sets from GSEA MsigDB 'all GO sets version 5' known as "Immunological Synapse", "Positive Regulation of Immune System Process", "Immune System Development", "Negative Regulation of Immune System Process", "Production of Molecular Mediator of Immune Response", "Positive Regulation of Immune Response", "Immune Effector Process", "Adaptive Immune Response GO 0002460", "Activation of Immune Response", "Immune Response", "Adaptive Immune Response", "Immune System Process", "Innate Immune Response", "Humoral Immune Response", "Regulation of Immune System Process", "Regulation of Immune Effector Process", "Regulation of Immune Response". These used all of the positive and/or general immune related pathways from the general and biological processes GO data sets. We then combined all 17 sets into one gene-set titled 'Immune" totaling 335 genes (Supplement table 3) after removing duplicates. This large gene list was created in an unsupervised fashion, which increases the

conservatism.

For the 'Interferon' gene-set enrichment analysis we pooled 29 interferon pathways available at GSEA[7] using MSigDB all the pathways for GO version 5. The pathways used were "GO Cellular Response to Interferon Beta", "GO Cellular Response to Interferon Gamma", "GO Interferon Gamma Mediated Signaling Pathway", "GO Interferon Gamma Production", "GO Negative Regulation of Interferon Gamma Production", "GO Negative Regulation of Interferon Alpha Production", "GO Positive Regulation of Interferon Alpha Production", "GO Positive Regulation of Interferon Beta Production", "GO Positive Regulation of Interferon Gamma Biosynthetic Process" "GO Positive Regulation of Interferon Gamma Production", "GO Positive Regulation of Type 1 Interferon Production", "GO Regulation of Interferon Alpha Production", "GO Regulation of Interferon Beta Production", "GO Regulation of Interferon Gamma Biosynthetic Process", "GO Regulation of Interferon Gamma Production", "GO Regulation of Interferon Gamma Secretion", "GO Regulation of Response to Interferon Gamma", "GO Regulation of Type 1 Interferon Mediated Signaling Pathway", "GO Regulation of Type 1 Interferon Production", "GO Response to Interferon Alpha", "GO Response to Interferon Beta", "GO Response to Interferon Gamma", "GO Response to Type 1 Interferon", "GO Type 1 Interferon Receptor Binding", "Hematopoietin Interferon Class D200 Domain Cytokine Receptor Activity", "Hematopoietin Interferon Class D200 Domain Cytokine Receptor Binding", "Interferon Gamma Biosynthetic Process", "Interferon Gamma Production", "Regulation of Interferon Gamma Biosynthetic Process". The combined 'Interferon' sets contained 433 unique genes merged from the list 29 pathways after removing duplicates.

The inflammation gene list used both (only 2) of the inflammation sets from MsigDB 'all GO sets version 5' defined as “Acute Inflammatory Response”, and “Inflammatory Response”. We also used 5 sets from the biological processes GO data base which were “GO Positive Regulation of Acute Inflammatory Response”, “GO Acute Inflammatory Response”, “GO Inflammatory Response”, “GO Regulation of Inflammatory Response”, “GO Positive Regulation of Inflammatory Response”. We uniquely combined these 7 sets to create a combined gene-set titled 'Inflammation' containing 649 genes (no duplicates) (Supplement table 3). adj.p.value of 0.025 to account for pairwise comparisons of Blast-LSC, and pHSC-LSC individually. For comparing low-risk to high-risk MDS we used an p.value of 0.05.

When investigating NF-κB activity , we similarly pooled gene-sets. We used “GO Regulation of NIK NF Kappa-Beta Signaling”, “GO Positive Regulation of NIK NF Kappa-Beta Signaling”, “GO Activation of NF Kappa-Beta Inducing Kinase Activity”, “GO NIK NF Kappa-Beta Signaling”, “GO Regulation of NF Kappa-Beta Import Into Nucleus”, “GO Positive Regulation of NF Kappa-Beta Import Into Nucleus”, “GO Positive Regulation of NF Kappa-Beta Transcription Factor Activity”, and pooled these 7 sets into a combined set titled “NFκB Combined Gene-Set” with 247 unique genes (Supplement table 3). We did not consider negative regulation of NF-kB. p.values that were used to determine significance used an adjusted p-value threshold of 0.0167, which accounted for the 3 pairwise comparisons of Blast, LSC and pHSC groups. The read cutoff for all pathway analyses was set to a minimum of 2 reads per gene, and the patient identifiers were used to conduct a paired, repeated measure, analysis.

We aimed to test large data sets to increase conservatism and reduce false positives in an unsupervised pathway analysis.

The global enrichment activation significance level threshold used in testing immune, inflammation, and interferon related sets used an adj.p.value of 0.025 to account for pairwise comparisons of Blast-LSC, and pHSC-LSC individually. For comparing low-risk to high-risk MDS we used an p.value of 0.05. The read cutoff for all pathway analyses was set to a minimum of 2 reads per gene, and the AML patient identifiers were used to conduct a paired, repeated measure, analysis in AML experiments only.

Pathway Analysis Using Combined Gene-Sets

The pathway analysis used QuSage[8] to construct the gene-set distribution activity and the gene expression level confidence intervals in a given gene-set. QuSage reduces the false positive rate by adjusting the variation inflation factors, which naturally arise upon comparing related genes.

Using the previously mentioned combined sets, we tested paired measurements using the patient identifiers for each pairwise comparison (LSC-Blast, LSC-pHSC, and Blast-pHSC). For the analysis of MDS we compared high-risk to low-risk MDS. In each pairwise test we used Bonferroni-Adjusted significance thresholds to adjust for multiple testing. A read minimum cutoff of 2 was used to measure library normalised CPM counts using edgeR in units of log base 2. The pathway analyses were always conducted in units of CPM for all data sets and all pairwise comparisons.

Differential Expression was called on the merged 'Immune' and 'Inflammation' gene-sets comparing LSC (Comparison) to Blast (Reference) (Supplemental Table 5) and plotted in units of TPM (Supplemental Figure3, and Supplemental Figure 6). Similarly differential expression was determined comparing high-low risk MDS.

Individual Gene Testing Using ANOVA

We tested the expression of individual genes across the stages of AML, and in MDS, some of those genes included EVI-1, ATG5, HSP90AA1, and LAMP2. The individuals gene expression levels were tested using ANOVA in units of TPM or CPM.

For the ANOVA conducted on units in CPM, between samples normalisation was calculating using EdgeR to normalise library size with a minimum read cutoff of 1. The ANOVA was fit using a linear model and the pairwise comparisons used Bonferroni adjust p.values to adjust for multiple comparisons for testing genes in AML which had paired measurements; MDS did not have paired measurements and used a significance threshold of 0.05 (non-adjusted).

Unsupervised Clusters of Patient Samples Based on Coding Gene Expression Correspondence to TE Type Expression in AML

Unsupervised clustering of patient samples used an unsupervised clustering of average Euclidean distance of the gene expression of all samples. The hierarchical clustering for each sample clustered average distance of expression using the unique ENSEMBL gene identifier.

The clustering of coding gene expression corresponding TE type expression used units of CPM and library normalisation edgeR. We performed hierarchical clustering of aggregated CPM counts of gene-ID/gene name and calculated the corresponding TE expression in terms of CPM.

The hierarchical clustering performed used WGCNA [9,10]. The gene expression used in the Euclidean cluster was log base 2 (1+CPM), the minimum read counts per gene considered was a minimum of 2 read CPM. We required filtering criteria of the clustering branch with a minimum branch of 2.

Coding Gene Networking Co-Relationship To Specific Transposable Element Types

The gene network construction used a soft-power threshold of 6 in WGNCA [9,10], which is the default parameter; further WGCNA publication suggests that the network module construction is robust per the soft thresholding power parameter value. Langfelder et. al suggest that the soft thresholding power level 6 suffices to achieve a scale-free-topology. In the construction of AML and MDS coding gene networks, we verified that the scale independence for both experiments was greater than 0.80 indicating a scale-free topology. Additionally, the gene network construction used a block construction and a signed network, setting the minimum gene module size to 30, and a maximum gene module size of 4000. For the construction of the AML gene network, we considered Blast and LSC gene expression, excluding pHSC. The gene network was formed using ENSEMBL gene identifiers, edgeR library normalisation, and a $\log_2$ transformation of units of CPM. MDS network construction was formed identically.

For both MDS and AML, each gene network was then correlated to the expression of the TE types selected as MULE, Pseudogene SAT, snRNA, TcMAR, DNA Transposon, PiggyBac, ERV1, L1, satellite, Endogenous Retrovirus, Repetetive Element, MIR, tel, Mariner/Tc1, HSFAU, SVA, CR1, SINE/tRNA, hAT, L2, Transposable Element, ERV3, ERVL, Merlin, ERVK, centromeric, Alu, and LTR Retrotransposon. The association of gene 'modules' to TE types was calculated by correlating each module's Eigen-value (PC1) to the sample's expression of individual TE type [9,10] (Supplement figure 5 and 7). In order to determine statistically significant associations between the samples' gene

expression in a given network to a specific TE type, a Pearson-correlation test was conducted on the samples' gene expression relatability of a given network to the samples' expression of a given TE type. A significance threshold of 0.05 was used.

Each gene module was tested for significant gene-set activation of the GO gene-sets version 5 (Supplement Figure 5, and 7). AML activation levels of the merged 'Interferon', 'Inflammation', and 'Immune' were plotted along the left-hand side the modules; note only the significantly activated gene-sets (p.value less than or equal to 0.05) were depicted (Figure 4, Figure 5D). We used units of CPM with library size normalisation in edgeR with a minimum read count of 2 for gene-set activation testing. For gene-set analysis, we used ENSEMBL gene names because MsigDB gene-sets were in terms of their naming symbols. The gene-set enrichment for MDS used 'Immune' and 'Inflammation' gene-sets only (Figure 5D).

Network construction of the AML and MDS experiments were done identically. The unconnected module defined as 'Grey' was discarded from all analyses.

Analyzing Gene Modules: Predictive Gene Expression of Network Modules, and Signed Connectivity of Regulatory Hub Genes

The supplements figures (Supplement figure 5 and 7) depict the relatability of each samples' gene expression to the network by a bar plot which indicates the level of covariance to the 1st principal component of the module Eigen-value; thus if a given sample has a high covariance to a given network module's Eigen-value (PC1), then this sample is likely to have high expression in this network (positively co-varies) with the adjacency matrix and vice versa. The Gene Module Eigen-values depicts which samples are predicted to covary with the network module and therefore have higher expression in this weighted network collection.

Most modules have more than 100 genes, so their names were not included in the supplementary figures 5 and 7. Most modules had genes, which were differentially expressed, and the DE genes' TPM expressions were included; if a module had less than 5 DE genes, the DE expression plot was omitted. The DE analysis was performed using previously described methods.

A power adjacency matrix is formed by calculating weighted pairwise correlations [9,10]. The regulatory intramodular connected elements is calculated by summing rows in a given adjacency matrix defined as Generalised Connectivity [9,10]. The top 50-65 connected genes in a given module with the most competitive intramodular connectivity scores of higher than 0.95 were plotted in units of TPM (supplemental figures 5 and 7).

Chi-Square Test and Fisher Test of Correlation and Activation Levels

After creating the gene module network and identifying the associations between the gene co-expression network modules to the expression of TE types, and testing for module set activations of the 'Interferon', 'Immune'/'Inflammation' gene-sets using previously described methods, we then tested the association between the correlation of TE types to the activation levels. For AML, we used the association table and created a contingency table by counting each TE type's positive/negative correlations and the corresponding positive/negative activity level for 'Interferon', 'Immune' and 'Inflammation' gene-set. Using two factors (+/-) correlations and (+/-) activity levels of the three merged gene-sets; we conducted a chi-square test by totaling every TE types' respective factors from the contingency table (ensuring large enough values). For individual TE types association between the two factors, a Fisher-Exact test was conducted using FDR controlling for multiple testing method titled 'Benjamani Hochberg' which adjusted for the 29 multiple Fisher-Exact tests on each of the 29 TE types (Supplement table 6). A significance level for the chi-square test measuring significance of association between correlations and enrichment activity of 3 gene-sets, a significance level used was 0.05. For individual pairwise Fisher Exact Test a significance level of 0.09 was used.

Pathways Potentially Mediating Suppression of Transposable Elements

Goodier et. al [11] described lines of defense against LINES and transposable elements and comprised a list of post-transcriptional, and RNA interference genes. There were 21 post-

transcriptional genes reviewed by Goodier et. al [11] which were AICDA, APOBEC1, APOBEC3, APOBEC, ATG5, ATG6, BECN1, CALCOCO2, HNRNPL, KIAA0430, LKAP, MOV10, MTRN1A, MT1, PABPC1, RNASEL, SAMHD1, SQSTM1, TEX19, TREX1, and ZC3HAV1. There were 24 genes in the RNA interfering gene set which included ASZ1, DDX4, DGCR8, DICER1, DCR1, DROSHA, EXD1, FKBP6, GTSF1, HENMT1, HSP90AA1, MAEL, MIR128-1, MOV10L1, PIWIL1, PIWIL2, PIWIL4, PLD6, TDRD1, TDRD5, TDRD9, TDRD12, TDRKH, TDRD2. For both MDS and AML, we tested these lists of genes in the identical method described for analyzing gene-set activity for coding gene-sets.

RNA Helicase genes were reported and reviewed by Umate et al [12] , and we expanded our analysis to the entire RNA DEAH-Box Helicases and analyzed the family using coding gene DE methods previously described.

In order to investigate the correlation of the differential expression of the post-transcriptional genes and RNA interference genes reviewed by Goodier, we fit the differential means to a linear model for each experiment (MDS and AML). Thus an R^2 value and a p-value were derived which measured the correlation of the differential means for each gene indicating a significant correlation of differentially expressed genes across experiments. This analysis was performed with a read cutoff of 2 in units of CPM normalised by edgeR.

ATACseq data analysis

ATACseq fastq reads were analyzed from Corces et al [1] . Patient trios were selected which had ATACseq data from pHSC, LSC and Blast samples. ATACseq reads were aligned against the hg19 reference genome using the bwa mem algorithm (https://arxiv.org/abs/1303.3997). Following the alignment, QC was performed for the samples. Only reads aligned to autosomes and sex chromosomes were considered. Mitochondrial reads were discarded.

All samples showed enrichment for transcription start sites (TSSs) sites. Enrichment was computed by comparing total reads falling into a window of 2kb just upstream of promoters to reads in a 5kb window distant from the TSS. An important visualisation tool for ATACseq data is the distribution of fragment sizes. A typical fragment size distribution showed a characteristic wiggle indicating large fraction of short nucleosome-free fragments and a lower fraction of fragments from regions protected by nucleosomes [13,14] . We used the getPESizes function from the *csaw* [15] library to interrogate the distribution of fragment sizes.

Our goal in this analysis was to compare changes in chromosome accessibility across cell-types. This falls within the framework of differential accessibility testing. Here we favored adoption of a window-based method [16] for detecting differentially accessible regions. Such approaches have been previously been implemented for ChIPseq data, notably in the package *csaw* [15] .

Reads were counted along the chromosomes in sliding windows of size 200bps. Windows with less than 10 reads were discarded. Further filtering was done by first computing a global background of reads distribution by counting reads in contiguous window size of 1000bps.

Composition bias across libraries was alleviated by normalising the libraries using the trimmed mean of M-vaues (TMM) method [17] . Read counts in contiguous windows of 1000bps were again considered for this purpose.

Post normalisation and filtering, the libraries were used for calling differentially accessible windows across cell-types. A paired design (Y ~ patient + cell type) was employed to perform a negative binomial regression using functions from the edgeR [18].. Differentially accessible windows for LSC versus Blast samples and LSC versus pHSC samples were tested for. Post accessibility testing, neighbouring windows were combined to define a region and significance level of the region computed from the p-values of the window-level tests. Multiple testing correction was then done at the region-level using a False Discovery Rate (FDR) cut-off of 0.01. For interpretability of the regions, the number of log-fold change increase (logFC UP) and log-fold change decrease (logFC DOWN) windows it contained were also computed (Supplement table 4). In addition, the p-value of the best window and the direction of change were also reported. Finally, all regions were annotated to report their distance from neighbouring genes.

Data Availability

The pHSC, Blast, and LSC RNA sequencing data is deposited in an SRA BioProject SRP065216.
The pHSC, Blast, and LSC ATAC sequencing data is deposited in an SRA BioProject SRP066100.

The analysis software for the study of TE and coding genes: Arkas RNA-Seq
The annotation software of coding genes: TxDbLite.

The scripts used in the networking analysis are available here:
The RNA-seq R data sets are provided in the "SummarizedExperiment" BioConductor format; the expression sets for both AML and MDS can be accessed using docker pull arcolombo/scientificreports:v1.

References:

1. Corces MR, Buenrostro JD, Wu B, et al. Lineage-specific and single-cell chromatin accessibility charts human hematopoiesis and leukemia evolution. *Nat Genet*. 2016;48(10):1193-1203. doi: 10.1038/ng.3646 [doi].

2. Wang H, Wen J, Chang CC, Zhou X. Discovering transcription and splicing networks in myelodysplastic syndromes. *PLoS One*. 2013;8(11):e79118. doi: 10.1371/journal.pone.0079118 [doi].

3. Bray NL, Pimentel H, Melsted P, Pachter L. Near-optimal probabilistic RNA-seq quantification. *Nat Biotechnol*. 2016;34(5):525-527. doi: 10.1038/nbt.3519 [doi].

4. Bao W, Kojima KK, Kohany O. Repbase update, a database of repetitive elements in eukaryotic genomes. *Mob DNA*. 2015;6:11-015-0041-9. eCollection 2015. doi: 10.1186/s13100-015-0041-9 [doi].

5. Ritchie ME, Phipson B, Wu D, et al. Limma powers differential expression analyses for RNA-sequencing and microarray studies. *Nucleic Acids Res*. 2015;43(7):e47. doi: 10.1093/nar/gkv007 [doi].

6. Robinson MD, McCarthy DJ, Smyth GK. edgeR: A bioconductor package for differential expression analysis of digital gene expression data. *Bioinformatics*. 2010;26(1):139-140. doi: 10.1093/bioinformatics/btp616 [doi].

7. Subramanian A, Tamayo P, Mootha VK, et al. Gene set enrichment analysis: A knowledge-based approach for interpreting genome-wide expression profiles. *Proc Natl Acad Sci U S A*. 2005;102(43):15545-15550. doi: 0506580102 [pii].

8. Yaari G, Bolen CR, Thakar J, Kleinstein SH. Quantitative set analysis for gene expression: A method to quantify gene set differential expression including gene-gene correlations. *Nucleic Acids Res*. 2013;41(18):e170. doi: 10.1093/nar/gkt660 [doi].

9. Langfelder P, Horvath S. WGCNA: An R package for weighted correlation network analysis. *BMC Bioinformatics*. 2008;9:559-2105-9-559. doi: 10.1186/1471-2105-9-559 [doi].

10. Langfelder P, Horvath S. Fast R functions for robust correlations and hierarchical clustering. *J Stat Softw*. 2012;46(11):i11. doi: i11 [pii].

11. Goodier JL. Restricting retrotransposons: A review. *Mob DNA*. 2016;7:16-016-0070-z. eCollection 2016. doi: 10.1186/s13100-016-0070-z [doi].

12. Umate P, Tuteja N, Tuteja R. Genome-wide comprehensive analysis of human helicases. *Commun Integr Biol*. 2011;4(1):118-137. doi: 10.4161/cib.4.1.13844 [doi].

13. Buenrostro JD, Giresi PG, Zaba LC, Chang HY, Greenleaf WJ. Transposition of native chromatin for fast and sensitive epigenomic profiling of open chromatin, DNA-binding proteins and nucleosome position. *Nat Methods*. 2013;10(12):1213-1218. doi: 10.1038/nmeth.2688 [doi].

14. Buenrostro JD, Wu B, Chang HY, Greenleaf WJ. ATAC-seq: A method for assaying chromatin accessibility genome-wide. *Curr Protoc Mol Biol*. 2015;109:21.29.1-9. doi: 10.1002/0471142727.mb2129s109 [doi].

15. Lun AT, Smyth GK. Csaw: A bioconductor package for differential binding analysis of ChIP-seq data using sliding windows. *Nucleic Acids Res*. 2016;44(5):e45. doi: 10.1093/nar/gkv1191 [doi].

16. Lun AT, Smyth GK. De novo detection of differentially bound regions for ChIP-seq data using peaks and windows: Controlling error rates correctly. *Nucleic Acids Res*. 2014;42(11):e95. doi: 10.1093/nar/gku351 [doi].

17. Robinson MD, Oshlack A. A scaling normalization method for differential expression analysis of RNA-seq data. *Genome Biol*. 2010;11(3):R25-2010-11-3-r25. Epub 2010 Mar 2. doi: 10.1186/gb-2010-11-3-r25 [doi].

18. Lun AT, Chen Y, Smyth GK. It's DE-licious: A recipe for differential expression analyses of RNA-seq experiments using quasi-likelihood methods in edgeR. *Methods Mol Biol*. 2016;1418:391-416. doi: 10.1007/978-1-4939-3578-9_19 [doi].
